# QSAR and Molecular Docking Studies of the Inhibitory Activity of Novel Heterocyclic GABA Analogues over GABA-AT

**DOI:** 10.3390/molecules23112984

**Published:** 2018-11-15

**Authors:** Josué Rodríguez-Lozada, Erika Tovar-Gudiño, Juan Alberto Guevara-Salazar, Rodrigo Said Razo-Hernández, Ángel Santiago, Nina Pastor, Mario Fernández-Zertuche

**Affiliations:** 1 Instituto de Investigación en Ciencias Básicas y Aplicadas, Centro de Investigaciones Químicas, Universidad Autónoma del Estado de Morelos, 62209 Cuernavaca, Morelos, Mexico; jrl@uaem.mx (J.R.-L.); egt@uaem.mx (E.T.-G.); 2 Departmento de Bioquímica, Escuela Superior de Medicina, Instituto Politécnico Nacional, 11340 Cd México, Mexico; jguevaras@ipn.mx; 3 Instituto de Investigación en Ciencias Básicas y Aplicadas, Centro de Investigación en Dinámica Celular, Universidad Autónoma del Estado de Morelos, 62209 Cuernavaca, Morelos, Mexico; rodrigo.razo@uaem.mx (R.S.R.-H.); jast@uaem.mx (Á.S.); nina@uaem.mx (N.P.)

**Keywords:** heterocyclic GABA analogues, GABA-AT enzyme, QSAR, GABA-AT docking, inhibitors

## Abstract

We have previously reported the synthesis, in vitro and in silico activities of new GABA analogues as inhibitors of the GABA-AT enzyme from *Pseudomonas fluorescens*, where the nitrogen atom at the γ-position is embedded in heterocyclic scaffolds. With the goal of finding more potent inhibitors, we now report the synthesis of a new set of GABA analogues with a broader variation of heterocyclic scaffolds at the γ-position such as thiazolidines, methyl-substituted piperidines, morpholine and thiomorpholine and determined their inhibitory potential over the GABA-AT enzyme from *Pseudomonas fluorescens*. These structural modifications led to compound **9b** which showed a 73% inhibition against this enzyme. In vivo studies with PTZ-induced seizures on male CD1 mice show that compound **9b** has a neuroprotective effect at a 0.50 mmole/kg dose. A QSAR study was carried out to find the molecular descriptors associated with the structural changes in the GABA scaffold to explain their inhibitory activity against GABA-AT. Employing 3D molecular descriptors allowed us to propose the GABA analogues enantiomeric active form. To evaluate the interaction with *Pseudomonas fluorescens* and human GABA-AT by molecular docking, the constructions of homology models was carried out. From these calculations, **9b** showed a strong interaction with both GABA-AT enzymes in agreement with experimental results and the QSAR model, which indicates that bulky ligands tend to be the better inhibitors especially those with a sulfur atom on their structure.

## 1. Introduction

There are two major neurotransmitters in the regulation of neuronal activity in the mammalian central nervous system, γ-aminobutyric acid (GABA) and L-glutamic acid. The former one is an inhibitory neurotransmitter and the latter an excitatory neurotransmitter [1,2]. GABA is synthesized via decarboxylation of L-glutamic acid by a process catalyzed by the enzyme glutamic acid decarboxylase (GAD) and metabolized by a transamination reaction catalyzed by the enzyme GABA-aminotransferase (GABA-AT) [3,4].

A decrease in GABA levels has been associated with a large group of psychiatric and neurological disorders such as epilepsy [5], Parkinson’s [6], Alzheimer’s [7] and Huntington [8] diseases among others.

GABA levels cannot be increased by direct administration of this compound due to its low lipophilic character, a condition that diminishes its capacity to cross the blood-brain barrier (BBB) [9]. As a result, there has been an intensive research activity oriented toward the design and synthesis of new GABA analogues with increased lipophilic character, capable of crossing the blood-brain barrier, for the treatment of the neurological disorders listed above.

In this context, two representative GABA analogues used in the clinic are (*S*)-pregabalin and (*R*)-baclofen. (*S*)-Pregabalin is an anticonvulsant, anxiolytic and analgesic drug believed to bind to the α2–δ protein, a voltage dependent calcium channel, reducing the release of excitatory neurotransmitters and peptide modulators that may be responsible for these effects [10,11,12,13,14]. (*R*)-Baclofen is a GABA_B_ agonist used on the treatment of spasticity, motility improvement of patients with multiple sclerosis, spinal cord injuries and muscle stiffness [15,16]. Due to their clinical importance, the synthesis and biological evaluation of analogues of these compounds, have been reported in the literature where several research groups have reported an impressive variety of modifications to their structures [17,18,19,20,21,22,23,24].

Another alternative to raise GABA concentration in the brain has been the design of GABA analogues with the potential to inactivate the GABA-AT enzyme, a pyridoxal 5’-phosphate (PLP)-dependent enzyme responsible for the degradation of this inhibitory neurotransmitter [25]. In this regard, vigabatrin (VGB) is the first GABA-AT inhibitor drug approved by the FDA for the treatment of epilepsy. VGB is a very selective and irreversible GABA-AT inhibitor raising the concentration of GABA on the brain [26,27,28].

Silverman has designed and evaluated a variety of GABA analogues as GABA-AT inhibitors [29,30,31,32], including CP-115, a compound 186 times more efficient in inactivating GABA-AT than VGB and with a high therapeutic potential for the treatment of epilepsies and cocaine addiction [33]. More recently, the Silverman group has reported a cyclopnet-1-ene derivative 10 times more efficient than VGB as a GABA-AT inactivator. In vivo studies show that this compound suppresses the release of dopamine after a cocaine or nicotine dose in rats [34].

The synthesis of bicyclic analogues of GABA has been reported by Conti [35]. There are also reports in the literature of GABA-AT inhibitors not related to the GABA framework such as sodium valproate (VPNa), a well-known antiepileptic compound whose mode of action has been attributed to an inhibition of the GABA-AT enzyme [36]. More recently, Siddiqui has reported the synthesis, in vitro GABA-AT screening and anticonvulsant activity of some dihydropyrimidine carbothioamides [37].

In the design of other type of GABA-AT inhibitors via computational methods, Bansal has designed novel GABA-AT inhibitors based on a molecular field analysis *k*NN-MFA 3DQSAR model for phenyl-substituted β-phenylethylidene hydrazine analogues [38,39]. Davood, identified β-phenylethylidene hydrazide GABA analogues with a variety of substituents at the phenyl ring by QSAR techniques [40]. On the other hand, Abdulfatai has reported QSAR models for the prediction of biological activities of quinoxaline and thiadiazoles derivatives as GABA-AT inhibitors [41,42] and Jain studied the effect of halogen substitution on the docking and 3DQSAR properties of a series of aryl-substituted thiosemicarbazones [43].

**Figure 1:**
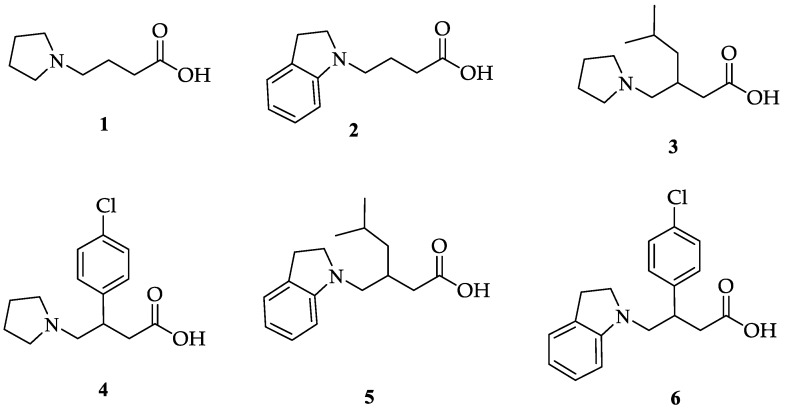
Pyrrolidine and indoline GABA analogues.

Our research group has previously reported the synthesis and biological activity of new GABA analogues in which the nitrogen atom is part of a heterocyclic system. Specifically, the inclusion of the nitrogen atom in a pyrrolidine or indoline ring system lead to GABA analogues such as **1**–**6** (Figure 1). In this work, **3** and **4** had inhibitory activity over the GABA-AT enzyme [44].

**Figure 2:**
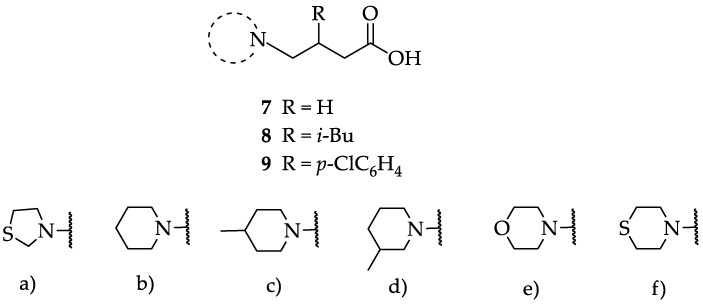
Structures of GABA analogues **7**, **8** and **9**.

We have evaluated the effect of other structural modifications, we present in this work the synthesis, in vitro and in silico evaluation of a new set of heterocyclic GABA analogues, **7**, **8** and **9** (Figure 2). These analogues maintain the GABA backbone where the nitrogen atom at the γ-position forms part of heterocyclic ring system. The thiazolidine ring systems were designed to evaluate the effect of a second heteroatom in a five-membered ring, piperidine and methyl substituted piperidines to evaluate ring size and the morpholine and thiomorpholine systems to evaluate the effect of heteroatoms in these six-membered ring systems. Piperidine-derived GABA analogues with a phenyl group at C-3 of the GABA backbone with vasoconstrictor properties have been reported in the literature [45].

Analogues **7** contain the GABA framework with-no substituents at the α- or β-positions. However, analogues **8** and **9** include an *iso*-butyl or *p*-Cl-phenyl substituents at the β-position in analogy to (*S*)-pregabalin and (*R*)-baclofen in order to improve their lipophilic character. Contrary to what happens in the case of GABA analogues with primary amine groups that allow them to react with the PLP cofactor of the GABA-AT enzyme, the inclusion of the nitrogen atom in these non-aromatic heterocyclic system makes them tertiary amines with higher basicity (pK_b_ ~ 4.2). This increased basicity favors the transfer of the acidic carboxylic proton to the nitrogen atoms generating zwitterionic species which may facilitate their interaction in a non-covalent manner, with the GABA-AT enzyme. We also report the in vitro inhibitory potential over the GABA-AT enzyme from *Pseudomonas fluorescens* as well as docking and Quantitative Structure-Activity Relationship (QSAR) studies to explain their GABA-AT inhibitory activity. QSAR analysis was used to propose which enantiomer has the greatest probability to be the biological active form, for this reason 3D molecular descriptors were used. Docking calculations help us to corroborate the QSAR hypothesis and study the interaction form of GABA analogues with GABA-AT. In addition, we also carried out a molecular docking study over a homology modeled human GABA-AT enzyme to identify compounds as potential candidates for future in vivo studies. All compounds comply with Lipinski’s rule of five thus predicting their probability to become an oral drug [46].

## 2. Results

### 2.1. Chemistry

The synthesis of the GABA analogues **7** is shown in Table 1. Although compounds **7b**, **7d**, **7e** and **7f** are commercially available and their use in synthesis has been reported in the literature [47,48,49], they were prepared by our previously reported *N*-alkylation and a hydrolysis sequence [44]. The *N*-alkylation reaction of **10a**–**f** with methyl-4-bromobutanoate (**11**) under solvent free conditions, was carried out at temperatures ranging between 65–70 °C affording esters **12** with moderate yields (58–67%). In a subsequent step, esters **12** were subjected to a basic hydrolysis with lithium hydroxide in aqueous methanol to afford analogues **7** with excellent yields

For the synthesis of the β-substituted analogues **8** and **9**, it was required to prepare the α,β-unsaturated esters **14**. As described previously, this was accomplished by the *N*-alkylation of heterocycles **10a**–**f** with commercially available **13** (Table 2).

The synthesis of the β-substituted analogues **8a**–**f** and **9a**–**f** are shown in Table 3 and Table 4 respectively, and involve the conjugate addition of cuprates **15** or **17**. As can be seen on Table 3, the addition of cuprate **7**, generated from *i*-butyl magnesium bromide and anhydrous CuI in ether at 0 °C, to the conjugated systems **14a**–**f** at 0 °C (entries a–f), affords the corresponding *i*-butyl substituted esters **8** in yields ranging from 13 to 75%. The low yields reported in entries a, c and f are attributed to decomposition of the corresponding heterocycles. Basic hydrolysis of esters **16**, to afford the corresponding β-substituted *i*-butyl analogues **8** was achieved with lithium hydroxide in aqueous methanol; the yields in this step were from moderate to very good.

Table 4 describes the synthesis of the β-aryl-substitutued analogues **9**. Addition of cuprate **17**, generated from bromochlorobenzene under the same reaction conditions, to the α,β-unsaturated esters **14a**–**f** afforded the β-aryl-substituted esters **18** in moderate yields (entries b–f). In the case of the thiazolidine ring system (entry a), it was not possible to obtain the corresponding ester, as decomposition of **14b** was observed under different reaction conditions. Once again, basic hydrolysis of esters **18**, carried out with lithium hydroxide in aqueous methanol led to the β-substituted analogues **9a**–**f** from moderate to very good yields. The β-substituted analogues in Table 3 and Table 4 were synthesized in racemic form in order to quickly identify inhibitory activity over the GABA-AT enzyme.

### 2.2. Enzymatic Inhibition

**Figure 3:**
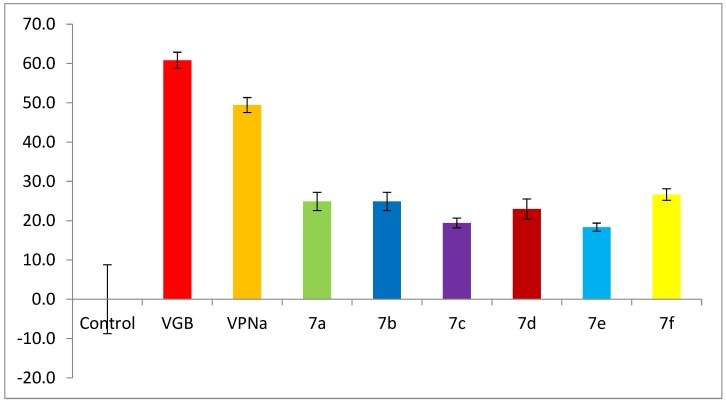
GABA-AT % enzymatic activity of compounds **7a**–**f** as compared to VGB and VPNa.

The enzymatic assay for GABA-AT inhibition is based on the role of this enzyme in the degradation of GABA to succinic semialdehyde, and its subsequent transformation to succinate. This involves the concomitant conversion of β-NADP^+^ to β-NADPH by a succinic-semialdehyde dehydrogenase-coupled reaction [26,50,51]. This is evidenced by the absorbance change at 340 nm, corresponding to the formation of β-NADPH from β-NADP^+^, which is proportional to GABA-AT activity. Figure 3 shows the inhibition of enzymatic catalytic activity of compounds **7a**–**f** over the GABA-AT enzyme from *Pseudomonas fluorescens*. The activity of these analogues was measured against the positive controls VGB and VPNa at a 0.8 mM concentration. As can be seen in Figure 3, compounds **7a**–**f** were inactive, as their catalytic inhibitory activity is lower than that of the positive controls.

**Figure 4:**
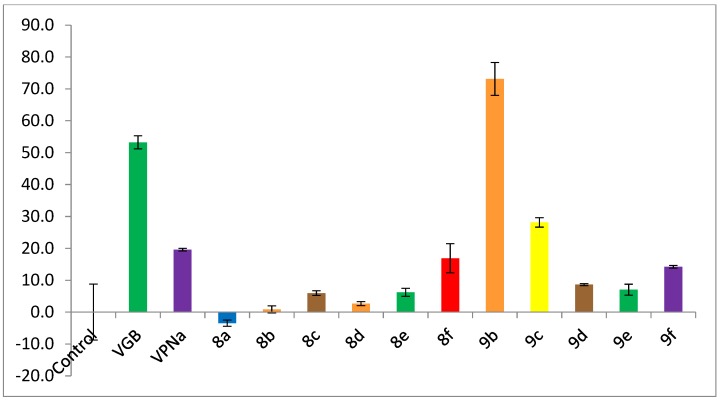
GABA-AT % enzymatic inhibition of compounds **8a**–**f** and **9a**–**f** as compared to VGB and VPNa.

Figure 4 shows the inhibition of enzymatic catalytic activity of compounds **8a**–**f** and **9a**–**f** over the GABA-AT enzyme from *Pseudomonas fluorescens*; all compounds were tested as racemic mixtures. Once again, the inhibitory activity of these analogues was measured against the positive controls VGB and VPNa at a 0.8 mM concentration. As can be seen in Figure 4, analogues **8a**–**e** have an enzymatic inhibitory activity much lower than the positive controls VGB and VPNa. Regarding analogues **9a**–**f**, the best compound is **9b**; compound **9b** displays a 73% inhibition over the enzyme, an inhibitory power greater than that of the control drugs VGB and VPNa.

### 2.3. In Vivo Studies

**Figure 5:**
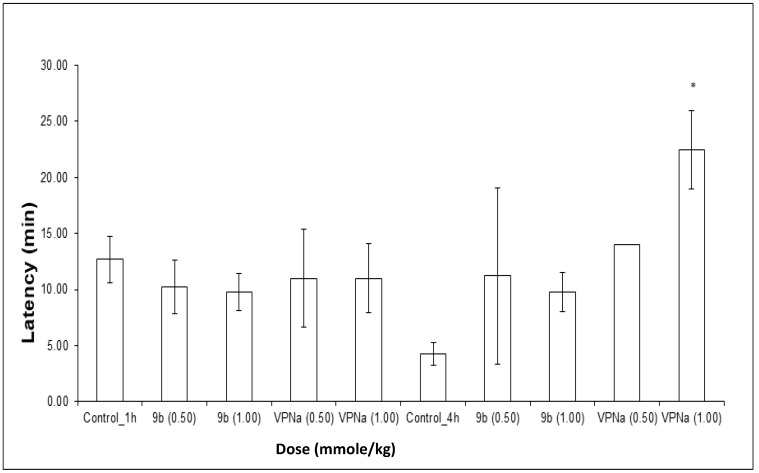
Latency of compound **9b** compared to VPNa at different doses (0.50 and 1.00 mmole/kg) at 1 h and 4 h of pretreatment. * *p* < 0.05: Significant difference comparing control group with **9b** and VPNa as a positive control group. Comparisons were made by the one-way ANOVA test Duncan’s means analysis test.

The experiments were carried out in two different times of pretreatment with **9b** and VPNa; 1 h and 4 h before administering PTZ. The results obtained in these tests consist of the analysis of the latency at the first seizure, the number of seizures generated in the observation period and the protection against death. In relation to the experiments carried out at 1 h, the latency of compound **9b** and VPNa with respect to the control, do not show significant difference, that is, at least at the doses tested there is no change in the generation time of the first seizure (Figure 5).

In the same way, the same type of results was analyzed for the test carried out with 4 h of pretreatment, in which regarding to latency, it can be observed that there is a slight tendency to increase the trend with compound **12b**, despite that there is no statistically significant difference. Only at a 1.00 mmole/kg dose of VPNa **4**, there was a clear increase in latency (Figure 5).

**Figure 6:**
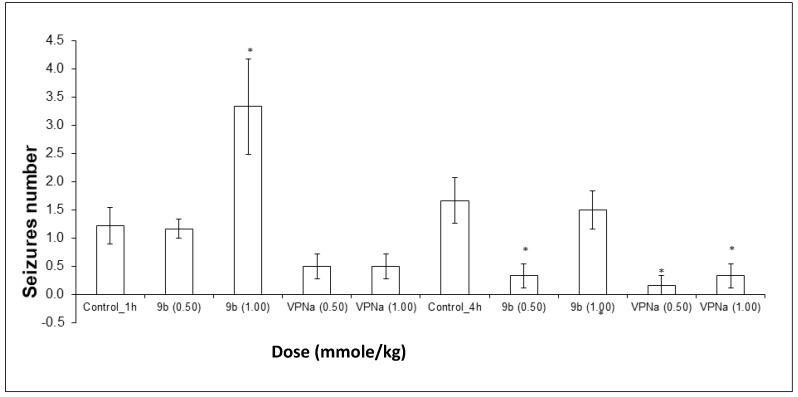
Tonic-clonic seizures number elicited by compound **9b** compared to VPNa at different doses (0.5 and 1.00 mmole/kg) at 1 h and 4h of pretreatment. * *p* < 0.05: Significant difference comparing control group with **9b** and VPNa as positive control group. Comparisons were made by the one-way ANOVA test and Duncan’s means analysis test [52,53,54].

On the other hand, the number of convulsions that each of the mice presented for each experimental group were counted, with the purpose of observing the anticonvulsant activity through the decrease in the number of them. It could be observed that at a 1.00 mmole/kg dose of **9b**, the number of seizures did not decrease, they rather increased significantly. At a 0.50 mmole/kg dose, there was no significant difference. In the case of the doses tested with VPNa, there was no significant difference; however, the results show a tendency towards a decrease in the number of seizures (Figure 6). In the case of the number of seizures generated during the observation time, the results obtained are quite interesting. First of all, the expected decreased in the number of seizures with VPNa was observed. However, in the case of compound **9b**, there is a very clear decrease in the number of seizures at a 0.5 mmole/kg dose. On the other hand, at a 1.00 mmole/kg dose, there is no difference with the control group, that is, compound **16b** does not have a protecting effect, at least at the highest dose against the number of seizures (Figure 6). Table 5 shows some parameters of anticonvulsive activity of compounds **9b** and VPNa in this model.

**Figure 7:**
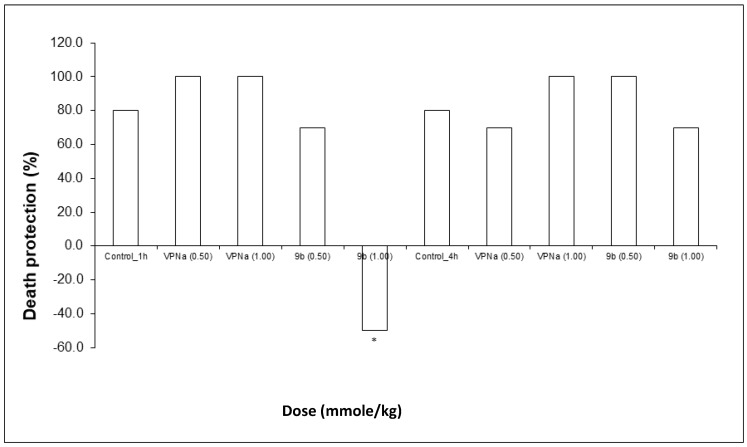
Percentage of death protection of compound **9b** compared to VPNa at different doses (0.50 and 1.00 mmole/kg) at 1 h and 4 h of pretreatment. * *p* < 0.05: Significant difference comparing control group with **9b** and VPNa as positive control group. Comparisons were made by the Fisher Exact test.

Finally, regarding death protection, VPNa, at both doses does protect against the lethal action of PTZ, whereas in the case of compound **9b**, there is protection at a 0.5 mmole/kg dose; at a 1.00 mmole/kg dose, the lethal action significantly increases (Figure 7). Finally, neither VPNa nor **9b**, have a significant difference, that is, practically no animals show a lethal effect from the statistical point of view (Table 5).

It is very important to mention that compound **9b** presents an atypical or non-dose-dependent behavior, and this occurs especially at the highest dose (1.00 mmole/kg) for the experiments at 1 h and 4 h of pretreatment before the administration of PTZ. Regarding the number of seizures, **9b** rather increases them, instead of decreasing, when the pretreatment is carried out during 1 h. When the pretreatment is carried out during 4 h there is no significant difference with the control group. However, at a dose of 0.50 mmole/kg there is a clear tendency to decrease the number of seizures, this phenomenon is probably due to a biphasic response or hormesis that has been reported in many substances with biological activity [55,56,57,58,59].

### 2.4. Computational Studies

#### 2.4.1. Conformational and Optimization Geometry

The optimized structures of the most stable conformers of all GABA analogues, including the *S* and *R* enantiomer of **8** and **9** series, are shown in Figure S45 (Supplementary Material). All vibrational frequencies of these were positive ensuring that all the structures are minima in the potential energy surface. 

#### 2.4.2. QSAR Analysis

Since one of our objectives is to understand the biological mechanism of our compounds, knowledge of which enantiomer will display the greatest probability to be the biologically active form was crucial for this study. Therefore, we employed geometrical and charge molecular descriptors for the construction of the QSAR models; these descriptors are sensitive to the spatial position of the atoms in the molecule and allowed us to study both enantiomers of each compound. Therefore, the inclusion of at least one of these descriptors in the final QSAR model was a requirement. The best mathematical model that correlates the inhibition percent (Y) of GABA analogues with their molecular descriptors, was validated by the QUIK, $Q_{ASYM}^{2}$, redundancy and overfitting rules. This expression, obtained with the (*S*)-enantiomers, is the following:

(1)  Y=1624.02548[RCI]−394.09487[HOMT]+0.4927[T(O..S)]+34.48134[TI2]−99.99144 

$R^{2}$ = 86.96 $Q_{LOO}^{2}$ = 73.03 s = 4.54 F = 21.3

ΔK = 0.003 (0.0) ΔQ = 0.075 (−0.005) $R^{P}=0.356\;$(0.1) $R^{N}=-$0.217 (−0.24)

Therefore, the GABA analogues molecular descriptors that are related to GABA-AT inhibition are: the Jug RC Index and the Harmonic Oscillator Model of Aromaticity index (RCI and HOMT respectively), which belong to the geometrical descriptors family. Additionally, in this mathematical model, the sum of the topological distance between the oxygen and sulfur atoms (*O..S*) and the second Mohar index (T(O..S) and TI2 respectively) from the topological descriptors family are included (Table 6). In addition, the experimental GABA-AT inhibition % ( Y) and predicted by the QSAR model ($Y_{pred}$) values of GABA derivatives are displayed.

In Equation (1), T(O..S) possess a positive coefficient indicating that if its value increases, the GABA-AT inhibition will also increase. T(O..S) indicates that the presence of a sulfur atom will increase the inhibitory activity of the GABA analogues, that is, increasing the value of T(O..S) is directly correlated to the molecule size. Therefore, increasing the distance between these two atoms in a compound, will increase its interactions with the GABA-AT enzyme.

As for *TI*2, its coefficient in Equation (1) is positive, indicating that increasing the size and spherical shape of the molecule will increase its inhibitory activity, *TI*2 is calculated from the eigenvalues of the Laplacian matrix as shown in Equation (2):

(2) $$\;TI2=\;\frac{4}{nSK\cdot \;\lambda_{nSK-1}}\;$$

where the *nSK* is the number of non-H atoms and $\lambda_{nSK-1}$ is the first non-zero eigenvalue.

For the RCI molecular descriptor, its coefficient in Equation (1) indicates that if the value of SPP descriptor increases, GABA-AT inhibition by the GABA analogues will be also enhanced. RCI is defined as an aromaticity index based on the idea of ring current, whose magnitude is determined by its weakest link in the ring [60]. The weakest link is calculated as the maximum bond distance of aromatic bonds (inversely proportional to the minimum total bond order).

HOMAT is derived from the HOMA index [61] and is calculated as follows:

(3) $$\;HOMT=B_{\pi }-\underset{k}{\sum }\alpha_{k}.\overset{B_{\pi k}}{\underset{b=1}{\sum }}{\left(r_{k}^{opt}-r_{b}\right)}^{2}\;$$

where $B_{\pi }$ is the total number of conjugated bonds, the sum runs over each conjugated bond type, $B_{\pi k}$ is the number of considered *π*-bond contributions of the *k*th conjugated bond type, *r_b_* is the actual bond length, *α_k_* and $r_{k}^{opt}$ are a numerical constant and the typical aromatic bond length referring to the *k*th aromatic bond type (see values in the table above). This descriptor depends on the conjugation degree of a molecule as well as on the total number of *π* bonds. According to the coefficient sign in equation 1 that corresponds to this descriptor, if the molecular aromaticity of the GABA derivatives increases its inhibitory activity will be enhanced.

The experimental GABA-AT inhibition % values (Y), calculated and predicted by the QSAR model ($Y_{cal}$ and $Y_{pred}$) are presented in Table 7. In addition, the absolute value of the differences between each $Y_{cal}$ and $Y_{pred}$ and Y, represented by the $residual_{cal}$ and $residual_{pred}$ terms respectively, are also shown.

**Figure 8:**
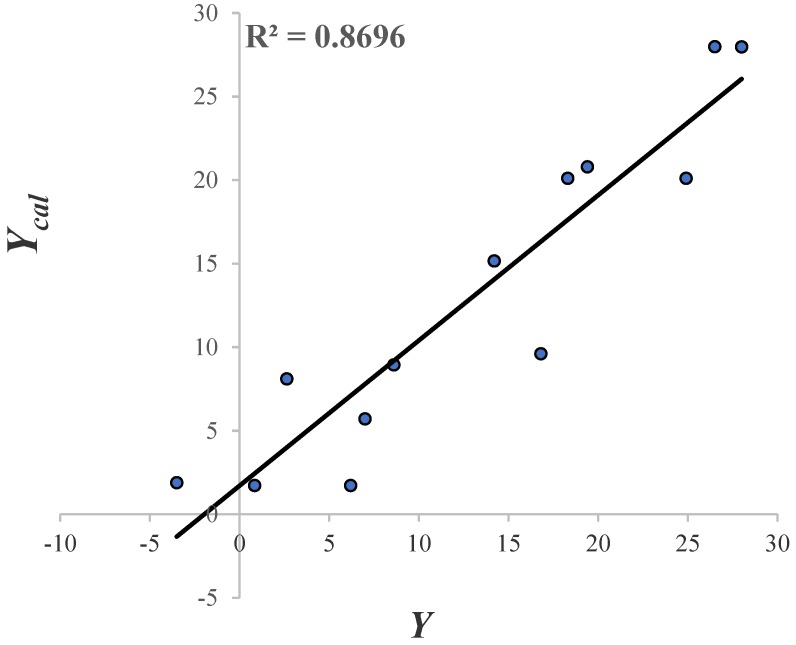
The linear correlation of $Y_{cal}\;\mathrm{vs}.Y$.

The molecules that form part of the test set are: **7a**, **7d** and **7c**. From Table 7. molecules **8a** and **8f** presented greater values of $residual_{pred}$. Perhaps, this may be related to the fact that they are the only two molecules of that series (pregabalin analogue type) that possess a sulfur atom in their structure. Due to the residual value of **9b** and VPNa, these compounds were considered as outliers; as their value is more than three times the value of the standard deviation [62]. The linear correlation of $Y_{cal}$ vs. and its $R^{2}$ = 0.87 is shown in Figure 8.

**Figure 9:**
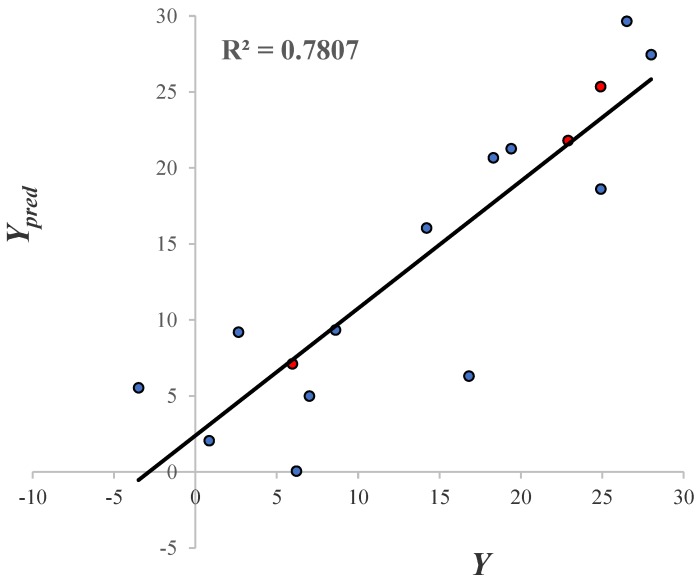
The linear correlation of $Y_{pred}\;\mathrm{vs}.\;Y$. Blue and red colored circles represent the training and test molecules, respectively.

The predictive ability of the mathematical model is shown in Figure 9. Since all the inhibition values of the GABA derivatives obtained with the leave one out technique (LOO) and the molecules that belong to the test set (color red circles) are displayed. The $R^{2}$ = 0.78 value indicates that the model has a good predictive power. The predictive ability evaluation of the QSAR model is displayed in Table S3 of the Supplementary Material.

The two graphs in Figure 8 and Figure 9 show the descriptive and predictive ability of the QSAR model. In general terms, the model possesses a respectable descriptive and predictive power (based on its $R^{2}$ and $Q^{2}$ values). Furthermore, the molecular descriptors in this model can help to explain the inhibitory activity displayed by the GABA analogues, based on the properties that can be important to the binding to GABA-AT. Since all the molecular descriptors included in the QSAR model can be related with the interaction with GABA-AT (molecular shape, size and aromaticity character), the docking results are important to confirm the descriptive power of our QSAR mathematical model.

#### 2.4.3. Molecular Docking for *Pseudomonas fluorescens* GABA-AT Model

**Figure 10:**
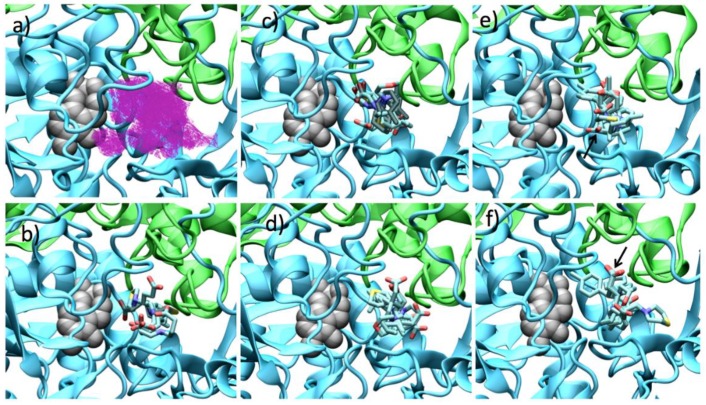
Docking results over *Pseudomonas fluorescens* GABA-AT catalytic site. (**a**) Cavity form (magenta color mesh representation) of the GABA-AT catalytic site. (**b**) GABA analogues **7**. (**c**) (*S*)-Pregabalin analogues **8**. (**d**) (*R*)-Pregabalin analogues **8**. (**e**) (*S*)-Baclofen analogues **9**. The best compound (**9b**) is indicated by an arrow. (**f**) (*R*)-Baclofen analogues **9**. The best compound (**9b**) is indicated by an arrow. PLP prosthetic group is showed as gray Van der Waals spheres and each protein chain is colored in green and cyan. The compounds where the carboxylic acid moiety is oriented to PLP are colored in obscure.

In order to explain the GABA-AT inhibition displayed by the GABA analogues, we carried out a molecular docking study over the *Pseudomonas fluorescens* homology model. We analyzed the molecular docking calculations by means of the scaffold structure (GABA, pregabalin and baclofen). Figure 10 shows the molecular docking of all GABA analogues over the *Pseudomonas* GABA-AT model. Figure 10 also shows the cavity shape where all the docking calculations were performed. It can be observed that this cavity has a “Y” like shape; this shape explains why analogues **7** were less potent. As was mentioned above, the T(O..S) and TI2 molecular descriptor, related to the molecular size that according to our QSAR model increases the GABA-AT inhibitory activity of all analogues, has the lower values for analogues **7** with respect to the **8** and **9** analogues. The specific molecular interactions of each molecule with *Pseudomonas* GABA-AT are shown in the Supplementary Material (Figures S46–S48).

The linear form of GABA analogues **7**, does not allow them to strongly interact with the cavity of GABA-AT enzyme (Figure 10b). In fact, analogues **7** display the lowest interaction energies (Table S1). All these molecules bind in a similar form with their carboxylic moiety oriented to the PLP molecule, except for **7**. This difference in the interaction form is maybe related to the presence of the sulfur atom and larger ring as compared to **7a**, like **8f** and **8f** (which also possess a sulfur atom in their structure) also interact in a different manner than that of the other analogues **8a**–**e** and **9a**–**e** molecules (pregabalin and baclofen analogues, respectively).

For the pregabalin analogues **8a**–**f**, it can be shown that the (*R*)-enantiomers bind in similar way to that of the (*S*)-enantiomers (Figure 10c,d), where the amine group of the molecule is oriented towards the PLP and the carboxylic group toward the outer part of the catalytic site, with the exception of **8f**. The (*S*)-pregabalin analogues possess a lower interaction energy compared to their (*R*)-enantiomers (Table S1).

**Figure 11:**
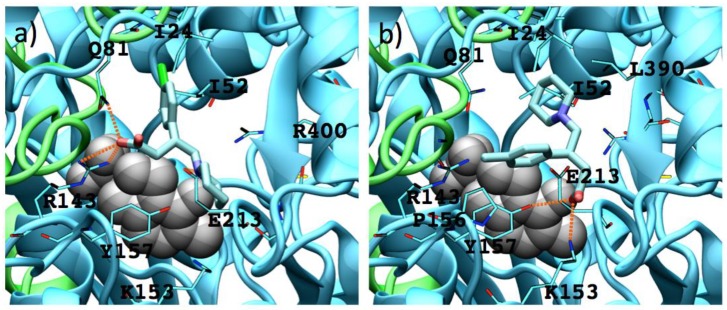
Interactions between **9b** enantiomers with *P. fluorescens* GABA-AT. (**a**) (*S*)-**9b** and (**b**) (*R*)-**9b**. PLP prosthetic group is showed as Van der Waals spheres and each protein chain is colored in green and cyan. Residues at 4 Å of each analogue are indicated. Hydrogen bonds are showed as orange dashed lines.

For analogues **9** as for analogues **8**, the (*S*)-enantiomers possess a better binding mode to GABA-AT (supported by their interaction energy values) than that of the (*R*)-enantiomers (Table S1). Due to their size and shape these molecules interact in the outer part of the catalytic site, so none of their functional groups is close to the PLP molecule. On the other hand, **9b**–**9d** interact in a similar way, their carboxylic group interacting with Arg 143, their amino group interacting with Glu 213 and Tyr 157, and their *p*-Cl-phenyl substituent located in the hydrophobic zone of the catalytic site formed by Ile 24, Ile 52, Val 82 and Cys 79. Figure 11 shows the interaction of (*S*) and (*R*) **9b** with the enzyme.

The (*S*)-**9b** molecule binds in a better and stronger way than (*R*)-**9b**, the appropriate orientation of their charged groups (amino and carboxylate moieties) favors hydrogen bond interactions with the GABA-AT residues (Tyr 157 for the amine group and Arg 143 and Gln 81 for the carboxylate group).

#### 2.4.4. Molecular Docking for Human GABA-AT Model

Since the main purpose of this work is to develop an alternative drug for clinical use that in the future may be used as a drug for humans, we also carried out a molecular docking study over a human GABA-AT model.

**Figure 12:**
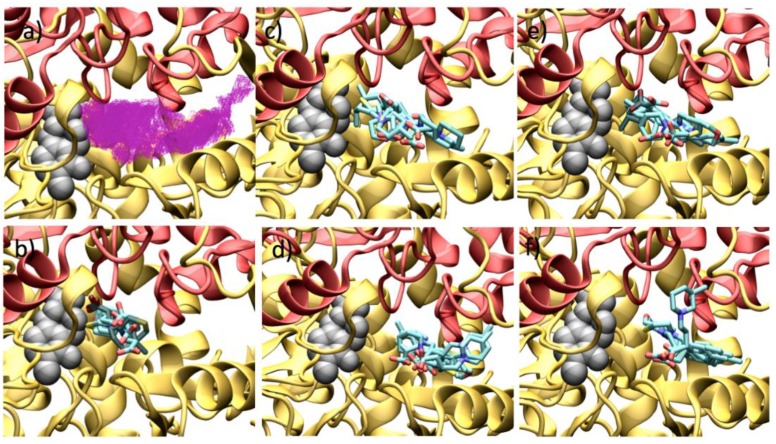
Docking results over *human* GABA-AT catalytic site. (**a**) Cavity form (magenta color mesh representation) of the GABA-AT catalytic site. (**b**) GABA analogues **7**. (**c**) (*S*)-Pregabalin analogues **8**. (**d**) (*R*)-Pregabalin analogues **8**. (**e**) (*S*)-Baclofen analogues **9**. (**f**) (*R*)-Baclofen analogues **9**. PLP prosthetic group is showed as gray Van der Waals spheres and each protein chain is colored in yellow and red. The compounds where the carboxylic moiety is oriented to PLP are colored in obscure.

Figure 12 shows the binding mode of all the GABA analogues over the human GABA-AT, and the cavity shape where all the docking calculations were done is also shown. It can be observed that this cavity possesses a greater volume that the *P. fluorescens* model and a different shape. Despite these differences, there is a recurring observation: molecules with a bigger size will possess better interaction energy values. The interactions of each molecule with human GABA-AT are shown in the Supplementary Material (Figures S49–S51).

In Figure 12b, the interaction of the GABA analogues **7a**–**f** is shown. The carboxylic acid group of compounds **7a**, **7b**, **7d** and **7e** is oriented to the outer part of the cavity (toward the solvent), and their amino group is close the PLP molecule. On the other hand, **7c** and **7f** show an opposite orientation, where the amino group is now oriented toward the solvent and the carboxylic acid group is close to the PLP molecule.

The enantiomeric forms of analogues **8a**–**f** interact in a similar way where their carboxylic acid groups are oriented to a high electrostatic positive zone of the cavity formed by Lys 193, Lys 429, Arg 412, His 196 and Arg 182. On the other hand, the amine group is oriented to the PLP molecule interacting with the His 34 residue. The interaction energy of (*S*)-analogues **8** is stronger than that of the (*R*)-enantiomers. (*R*)-analogues **8a**, **8c** and **8d** interact in a different form that the other analogues **8b**, **8e** and **8f**, orienting their amino group to the His 34 rather than to the PLP molecule.

For analogues **9**, their interaction with human GABA-AT was very similar with their carboxylic acid groups oriented to the positive zone of the cavity catalytic site. In the (*S*)-analogues, almost all the compounds oriented their amine group to Ile 416, and only **9b** and **9c** oriented their amine function to the PLP molecule. On the other hand, all the (*R*)-analogues bind in a similar way, where their *p*-Cl-phenyl substituent interacts with the Ile 416 residue, while their amine group is oriented to the PLP molecule.

According to this approximation and based on its interaction energy value, the most potent possible inhibitor will be (*S*)-**8f** (Table S2, Supplementary Material). All analogues **8** and **9** display very good interaction energies with *human* GABA-AT. In addition, (*S*)-**9c** has an interaction energy value that is one of the lowest. These results are related with the one obtained with the *Pseudomonas* GABA-AT, since the ligands with greater volume and with a sulfur atom, possess the lowest interaction energy (better inhibitors). This fact gives us a higher probability of success to transfer our results to a human model.

## 3. Materials and Methods

### 3.1. General Information

Oxygen and/or moisture sensitive reactions were carried out in oven or flame-dried glassware under nitrogen atmosphere. All reagents and solvents were purchased and used as received from commercial sources or synthesized according to cited procedures. THF, Ether and toluene were dried by distillation over sodium benzophenone ketyl. All other solvents were used after distillation at normal pressure. Yields refer to chromatographically and spectroscopically pure compounds, unless otherwise stated. Analytical thin layer chromatography (TLC) was performed on 0.25 mm silica gel 60-F plates and visualized by UV light (254 nm) and/or iodine vapor.

^1^H-NMR (400 MHz) and ^13^C-NMR (100 MHz) spectra were recorded on a Innova 400 MHz (9.4T) or Mercury 200 or 400 MHz (9.4T) spectrometer (Varian, Palo Alto, CA, USA; chemical shifts (δ) are reported relative to the signal of Me_4_Si. NMR data are reported as follows: chemical shift (δ ppm), multiplicity (s = singlet, br s = broad singlet, d = doublet, t = triplet, q = quartet, sext = sextet, dd = doublet of doublets, m = multiplet or overlapping), coupling constant (Hz), integration. Mass spectra (FAB+) were measured with a JMS700 mass spectrometer (JEOL, Peabody, MA, USA).

### 3.2. Chemistry

#### 3.2.1. Methyl 4-Bromobutanoate (**11**)

4-Bromobutanoic acid (7.5 g, 45.2 mmole) was dissolved in methanol (50 mL), and then CH_3_SiCl (11.4 mL, 90.4 mmole) was added at 0 °C; the mixture was stirred at this temperature for 30 min and for 72 h at 25 °C. The solvent was removed in vacuuo. The resultant residue was purified by column chromatography (silica-gel) eluting with hexanes:ethyl acetate 90:10 to afford the title compound as a colorless oil (6.78 g, 83.4%). ^1^H-NMR (CDCl_3_): δ 3.68 (s, 3H), 3.46 (t, *J* = 6.4 Hz, 2H), 2.50 (t, *J* = 7.2 Hz, 2H), 2.22–2.13 (m, 2H). ^13^C-NMR (CDCl_3_): δ 173.11, 51.85, 32.80, 32.34, 27.87. HRMS (FAB+): *m*/*z* [M]^+^ calcd for C_5_H_9_BrO_2_: 179.9786, found (M + 1): 180.9786.

#### 3.2.2. General Methodology for the Synthesis of Esters **12a**–**f**

The corresponding amine **10a**–**f** (1.0 equiv) was added dropwise to methyl 4-bromobutanoate (**2**, 1.0 equiv) at 25 °C, and the mixture was allowed to warm to 65–70 °C for 2 h. When the reaction was complete, aqueous NaHCO_3_ was added, and the mixture was extracted with EtOAc (3 × 10 mL), dried over Na_2_SO_4_, filtered and removed in vacuuo. The crude product was purified by column chromatography (silica-gel), eluting with hexanes: ethyl acetate 90:10.

*Methyl 4-(thiazolidin-3-yl)butanoate* (**12a**). 0.445 g (37%) as a yellow oil. ^1^H-NMR (200 MHz, CDCl_3_): δ 4.00 (s, 2H), 3.63 (s, 1H), 3.02 (dd, *J* = 9.5, 3.3 Hz, 2H), 2.82 (dd, *J* = 9.3, 3.6 Hz, 1H), 2.37 (t, *J* = 7.4, 2H), 2.35 (t, *J* = 7 Hz, 2H), 1.77 (q, *J* = 7.2 Hz, 1H, H-4). ^13^C-NMR (50 MHz, CDCl_3_) δ 173.94, 60.44, 58.01, 52.07, 51.62, 31.71, 29.6, 24.28. HRMS (FAB+): *m*/*z* [M]+ calcd for C_8_H_15_NO_2_S: 189.0823, found (M + 1): 190.0904.

*Methyl 4-(piperidin-1-yl) butanoate* (**12b**). 0.37 g (67%) as a yellow oil. ^1^H-NMR (CDCl_3_) δ 3.62 (s, 3H), 2.33 (sa, 4H), 2.28 (t, *J* = 8 Hz, 2H), 2.27 (t, *J* = 8 Hz, 2H), 1.77 (q, *J* = 8 Hz, 2H), 1.55–1.49 (m, 4H), 1.4–1.37 (m, 2H). ^13^C-NMR (CDCl_3_) δ 174.13, 58.49, 54.54, 51.56, 32.23, 25.98, 24.48, 22.24. HRMS (FAB+): *m*/*z* calcd for C_10_H_19_NO_2_: 185.1416, found (M + 1): 186.1532.

*Methyl 4-(3-methylpiperidin-1-yl) butanoate* (**12c**). 0.537 g (79%) as a yellow oil. ^1^H-NMR (CDCl_3_): δ 3.67 (s, 3H), 2.84–2.78 (m, 2H), 2.35–2.29 (m, 4H), 1.87–1.77 (m, 3H), 1.71–1.49 (m, 6H), 0.85 (d, *J* = 6.4 Hz, 3H). ^13^C-NMR (CDCl_3_) δ 174.13, 62.08, 58.25, 54.02, 51.54, 33.14, 32.22, 31.18, 25.62, 22.30, 19.83. HRMS (FAB+): *m*/*z* calcd for C_11_H_21_NO_2_: 199.1572, found (M + 1): 200.1636.

*Methyl 4-(4-methylpiperidin-1-yl)butanoate* (**12d**). 0.45 g (67%) as a yellow oil. ^1^H-NMR (CDCl_3_): δ 3.67 (s, 3H), 2.87–2.84 (m, 2H), 2.35–2.3 (m, 4H), 1.92–1.86 (m, 2H), 1.85–1.77 (m, 2H), 1.62–1.58 (m, 2H), 1.40–1.28 (m, 1H), 1.28 (m, 2H), 0.91 (d, *J* = 6.4 Hz, 3H). ^13^C-NMR (CDCl_3_) δ 174.18, 58.21, 54.05, 51.61, 34.43, 32.30, 30.93, 22.47, 22.02. HRMS (FAB+): *m*/*z* calcd for C_11_H_21_NO_2_, 199.1572, found (M + 1): 200.1656.

*Methyl 4-morpholinobutanoate* (**12e**). 0.445 g (52%) as a yellow oil. ^1^H-NMR (CDCl_3_): δ 3.7 (dd, *J* = 4, 4 Hz, 4H), 3.68 (s, 3H), 2.43 (dd, *J* = 4, 4 Hz, 4H), 2.36 (t, *J* = 8 Hz, 4H), 1.82 (q, *J* = 8 Hz, 1H). ^13^C-NMR (CDCl_3_) δ 173.92, 66.92, 57.97, 53.55, 51.48, 31.87, 21.73. HRMS (FAB+): *m*/*z* calcd for C_9_H_17_NO_3_: 187.1208, found (M + 1): 188.1308.

*Methyl 4-thiomorpholinobutanoate* (**12f**). 0.47 g (58%) as a yellow oil. ^1^H-NMR (CDCl_3_): δ 3.60 (s, 3H), 2.64–2.62 (m, 4H), 2.6–2.58 (m, 4H), 2.31 (t, *J* = 8 Hz), 2.26 (t, *J* = 8Hz), 1.73 (q, *J* = 8 Hz). ^13^C-NMR (CDCl_3_) δ 173.93, 58.24, 54.92, 51.47, 31.88, 27.93 (2C), 21.78. HRMS (FAB+): *m*/*z* calcd for C_9_H_17_NO_2_S: 203.098, found (M + 1): 204.1057.

#### 3.2.3. General Methodology for Hydrolysis of Methyl Esters **12a**–**f**

The corresponding methyl ester **12a**–**f** (1.0 equiv) was dissolved in MeOH (6 mL) and NaOH (1.1 equiv) dissolved in water (2 mL) was added dropwise. The reaction was monitored until the consumption of the starting material. At the end of the reaction, the MeOH was evaporated. Then HCl was added to pH = 5, the mixture was extracted with EtOAc (3 × 5 mL), dried over Na_2_SO_4_, filtered and the solvent removed in vacuo.

*4-(Thiazolidin-3-yl)butanoic acid* (**7a**). 0.165 g (81%) as a yellow oil. ^1^H-NMR (200 MHz, CD_3_OD) δ 4.02 (s, 2H), 3.08 (t, *J*= 5.9 Hz, 2H), 2.88 (t, *J* = 6.2 Hz, 2H), 2.54–2.41 (m, 2H), 2.29 (t, *J*= 7.3 Hz, 2H), 1.90–1.68 (m, 2H). ^13^C-NMR (50 MHz, CD_3_OD) δ 180.16, 60.29, 58.68, 53.73, 35.05, 29.94, 26.13. HRMS (FAB+): *m*/*z* calcd for C7H13NO2S, 175.0667, experimental (M + 1): 176.0769.

*4-(Piperidin-1-yl)butanoic acid* (**7b**). 0.22 g (95%) as a yellow oil. ^1^H-NMR (CD_3_OD): δ 3.3–3.12 (sa, 2H), 3.08 (t, *J* = 7.2 Hz, 2H), 2.74 (ddd, *J* = 15.5, 12, 12 Hz, 2H), 2.41 (t, *J* = 6.6 Hz, 2H), 2.01–1.92 (m, 2H), 1.86–1.84 (m, 4H), 1.66 (sa, 2H). ^13^C-NMR (CD_3_OD) δ 181.22, 59.77, 54.39, 37.50, 24.73, 23.15, 21.54HRMS (FAB+): *m*/*z* calcd for C_9_H_17_NO_2_: 171.1259, found (M + 1): 172.1324.

*4-(3-Methylpiperidin-1-yl)butanoic acid* (**7c**). 0.21 g (93%) as a colorless oil. ^1^H-NMR (D_2_O): δ 2.92 (m, 2H), 2.46–2.41 (m, 2H), 2.09 (td, *J* = 12.4, 2.6 Hz, 1H), 2.02 (t, *J* = 7.3 Hz, 2H), 1.81 (t, *J* = 11.4 Hz, 1H), 1.68–1.47 (m, 5H), 1.46–1.34 (m, 1H), 0.86–0.75 (m, 1H), 0.71 (d, *J* = 6.5 Hz, 3H). ^13^C-NMR (D_2_O) δ 182.34, 59.72, 57.37, 52.70, 35.20, 31.10, 29.65, 23.62, 21.53, 18.45. HRMS (FAB+): *m*/*z* calcd for C_10_H_19_NO_2_: 185.1416, found (M + 1): 186.1532.

*4-(4-Methylpiperidin-1-yl)butanoic acid* (**7d**). 0.14 g (80%) as a colorless oil. ^1^H-NMR (D_2_O): δ 2.81 (d, *J* = 11.6 Hz, 2H), 2.30–2.22 (m, 2H), 2.02–1.96 (m, 4H), 1.63–1.46 (m, 4H), 1.28–1.21 (m, 1H), 1.05–0.95 (m, 2H), 0.71 (d, *J* = 6.5 Hz). ^13^C-NMR (D_2_O) δ 182.67, 57.36, 52.78, 35.41, 32.57, 29.41, 22.13, 20.84. HRMS (FAB+): *m*/*z* calcd for C_10_H_19_NO_2_: 185.1416, found (M + 1): 186.1532.

*4-Morpholinobutanoic acid* (**7e**). 0.22 g (83%) of a yellow solid, m.p = 73–73.5 °C. ^1^H-NMR (CD_3_OD): δ 3.73–3.66 (m, 4H), 2.46 (s, 4H), 2.36 (m, 2H), 2.17 (t, *J* = 7.5 Hz), 1.79 (q, *J* = 7.5 Hz). ^13^C-NMR (CD_3_OD) δ 180.87, 66.28 (2C), 58.7, 53.41 (2C), 35.61, 22.89. HRMS (FAB+): *m*/*z* calcd for C_8_H_15_NO_3_: 173.11, found (M + 1): 174.1171.

*4-Thiomorpholinobutanoic acid* (**7f**). 0.22 g (83%) of a yellow solid, m.p = 82–83 °C. ^1^H-NMR (CDCl_3_): δ 2.77–2.70 (m, 4H), 2.66–2.65 (m, 4H), 2.41–2.37 (m, 2H), 2.14 (t, *J* = 7.5 Hz, 2H), 1.78 (q, *J* = 7.6 Hz). ^13^C-NMR (CDCl_3_) δ 182.32, 60.37, 56.20 (2C), 36.95, 28.37 (2C), 24.14. HRMS (FAB+): *m*/*z* calcd for C_8_H_15_NO_2_S: 189.08, found (M + 1): 190.1053.

#### 3.2.4. General Procedure for the Synthesis of Esters **14a**–**f**

Amines **10a**–**f** (1.0 equiv), *N,N*-diisopropylethylamine (1.5 equiv), ethyl (*E*)-4-bromobut-2-enoate (**5**, 1.3 equiv) and CH_2_Cl_2_ (25 mL) were placed in a round bottom flask and stirred at −20 °C for 1 h, under nitrogen. After completion of the reaction (TLC) the reaction mixture was extracted with NaHCO_3_ (3 × 30 mL) and water (1 × 30 mL). The organic phase was dried over Na_2_SO_4_ and the solvent removed under reduced pressure to yield the crude product. The crude product was purified by silica gel column chromatography (9:1 hexanes:EtOAc).

*Ethyl (E)-4-(thiazolidin-3-yl)but-2-enoate* (**14a**). 1.21 g (63 %) as a yellow oil. ^1^H-NMR (CDCl_3_) δ 6.93 (dt, *J* = 16, 8 Hz, 1H), 6.01 (dt, *J* = 16, 1.8 Hz, 1H), 4.18 (q, *J* = 7 Hz, 2H), 4.03 (s, 2H), 3.15 (dd, *J* = 8, 1.8 Hz, 2H), 3.07 (t, *J* = 6.4 Hz, 2H), 2.87 (t, *J* = 6.4 Hz, 2H), 1.27 (t, *J* = 7 Hz, 3H). ^13^C-NMR (CDCl_3_) δ 166.24, 144.94, 123.42, 60.59 (2C), 57.92, 53.90, 29.62, 14.34. HRMS (FAB+): *m*/*z* calcd for C_8_H_15_NO_2_S: 201.284, found (M + 1): 202.0912.

*Ethyl (E)-4-(piperidin-1-yl)but-2-enoate* (**14b**). 0.9 g (90 %) as a yellow oil. ^1^H-NMR (CDCl_3_): δ 6.95 (dt, *J* = 15.7, 6.3 Hz, 1H), 5.92 (dt, *J* = 15.7, 1.6 Hz, 1H), 4.16 (q, *J* = 7.1 Hz, 2H), 3.07 (dd, *J* = 6.3, 1.6 Hz, 2H), 2.37 (sa, 4H), 1.56 (q, *J* = 5.6 Hz, 4H), 1.44–1.38 (m, 2H), 1.25 (t, *J* = 7.1 Hz, 3H). ^13^C-NMR (CDCl_3_) δ 166.39, 145.69, 123.34, 60.45, 60.25, 54.84 (2C), 26.05 (2C), 24.24, 14.39. HRMS (FAB+): *m*/*z* calcd for C_11_H_19_NO_2_: 197.1416, found (M + 1): 198.1506.

*Ethyl (E)-4-(3-methylpiperidin-1-yl)but-2-enoate* (**14c**). 0.83 g (77%) as a yellow oil. ^1^H-NMR (CDCl_3_): δ 6.96 (dt, *J* = 15.7, 6.3 Hz, 1H), 5.94 (dt, *J* = 15.7, 1.6 Hz, 1H), 4.17 (q, *J* = 7.2 Hz, 2H), 3.09 (dd, *J* = 6.3, 1.6 Hz, 2H), 2.85–2.74 (m, 2H), 1.87 (td, *J* = 11.3, 3.2 Hz), 1.71–1.54 (m, 5H), 1.27 (t, *J* = 7.2 Hz, 3H), 0.84 (d, *J* = 6.4 Hz, 4H). ^13^C-NMR (CDCl_3_) δ 166.44, 145.63, 123.43, 62.22, 60.50, 60.01, 54.32, 32.90, 31.31, 25.65, 19.83, 14.42. HRMS (FAB+): *m*/*z* calcd for C_12_H_21_NO_2_: 211.1572, found (M + 1): 212.1651.

*Ethyl (E)-4-(4-methylpiperidin-1-yl)but-2-enoate* (**14d**). 0.85 g (80%) as a yellow oil. ^1^H-NMR (CDCl_3_) δ 6.94 (dt, *J* = 15.6, 6.4 Hz, 1H), 5.92 (dt, *J* = 15.5, 1.6 Hz, 1H), 4.15 (q, *J* = 7.1 Hz, 2H), 3.08 (dd, *J* = 6.4, 1.6 Hz, 2H), 2.82 (d, *J* = 11.8 Hz, 4H), 2.02–1.80 (m, 2H), 1.65–1.55 (m, 2H), 1.38–1.31 (m, 1H), 1.25 (t, *J* = 7.1 Hz, 3H), 0.89 (d, *J* = 5.7 Hz, 3H). ^13^C-NMR (CDCl_3_) δ 166.46, 145.77, 123.42, 60.53, 59.96, 54.35 (2C), 34.45 (2C), 30.73, 22.06, 14.46. HRMS (FAB+): *m*/*z* calcd for C_12_H_21_NO_2_: 211.1572, found (M + 1): 212.1651.

*Ethyl (E)-4-morpholinobut-2-enoate* (**14e**). 1.02 g (89%) as a yellow oil. ^1^H-NMR (200 MHz, CDCl_3_) δ 6.91 (dt, *J* = 15.7, 6.2 Hz, 1H), 5.96 (dt, *J* = 15.7, 1.6 Hz, 1H), 4.17 (q, *J* = 7.1 Hz, 2H), 3.72–3.67 (m, 4H), 3.1 (dd, *J* = 6.2, 1.6 Hz, 2H), 2.49–2.39 (m, 4H), 1.26 (t, *J* = 7.1 Hz, 3H). ^13^C-NMR (50 MHz, CDCl_3_) δ 166.05, 144.38, 123.65, 66.83 (2C), 60.38, 59.55, 53.64 (2C), 14.20. HRMS (FAB+): *m*/*z* calcd for C_10_H_17_NO_3_: 199.1208, found (M + 1): 200.1291.

*Ethyl (E)-4-thiomorpholinobut-2-enoate* (**14f**). 0.85 g (80%) as a yellow oil. ^1^H-NMR (CDCl_3_) δ 6.88 (dt, *J* = 15.7, 6.1 Hz, 1H), 5.94 (dt, *J* = 15.7, 1.7 Hz, 1H), 4.16 (q, *J* = 7.1 Hz, 2H), 3.11 (dd, *J* = 6.1, 1.7 Hz, 2H), 2.67 (m, 8H), 1.25 (t, *J* = 7.1 Hz, 3H). ^13^C-NMR (CDCl_3_) δ 166.00, 144.72, 123.43, 60.30, 59.91, 54.97 (2C), 27.88 (2C), 14.15. HRMS (FAB+): *m*/*z* calcd for C_12_H_21_NO_2_: 215.098, found (M + 1): 216.1044.

#### 3.2.5. General Methodology for the Conjugate Additions of **15**, **17**

Most of the reactions were carried out using the following procedure: CuI (2.00 equiv) was placed in a one-neck 50 mL round-bottom flask equipped with a stirring bar and sealed with a septum. The flask was evacuated with a vacuum pump and then purged with nitrogen, this process was repeated three times to assure a complete nitrogen atmosphere. Anhydrous ethyl ether (25.00 mL) was injected and the mixture stirred for 15 min and then cooled to 0 °C. Simultaneously, a Grignard solution was prepared mixing finely divided Mg turnings (4.1 equiv) and catalytic amounts of I_2_, with 1-bromo-2-methylpropane (4.0 equiv) or 1-bromo-4-chlorobenzene (4.0 equiv) at room temperature. The mixture was stirred for 30 min and then transferred via syringe dropwise to the copper solution at 0 °C, and stirred at this temperature for another 30 min period. Then, the unsaturated substrate **14a**–**f** (1.0 equiv) were added dropwise. The reaction was allowed to proceed for 24 h before being quenched with a saturated aqueous solution of NH_4_Cl. The mixture was extracted with ether (3 × 25 mL) dryed over Na_2_SO_4_ and the solvent removed in vacuo to afford the crude products which were purified by flash chromatography eluting with hexane:EtOAc (95:5). Some modifications done to this procedure are mentioned in the specific examples below.

*Ethyl 5-methyl-3-(thiazolidin-3-ylmethyl)hexanoate* (**16a**). 0.023 g (5%) as a yellow oil. ^1^H-NMR (CDCl_3_): δ 4.16–4.07 (m, 2H), 4.02 (q, *J* = 9.2 Hz, 2H), 3.04–3.0 (m, 2H), 2.90–2.82 (m, 2H), 2.44–2.36 (m, 2H), 2.27 (dd, *J* = 15.1, 5.6 Hz, 1H), 2.12–2.06 (m, 2H), 1.64 (m, 1H), 1.25 (t, *J* = 7.1 Hz, 3H), 1.22–1.08 (m, 2H), 0.89 (t, *J* = 6.4 Hz, 6H). ^13^C-NMR (CDCl_3_) δ 173.54, 61.44, 60.25, 58.65, 58.14, 42.40, 38.58, 33.28, 29.88, 25.40, 23.02, 22.77, 14.42. HRMS (FAB+): *m*/*z* calcd for C_13_H_25_NO_2_S: 259.1606, found (M + 1): 260.1699.

*Ethyl 5-methyl-3-(piperidin-1-ylmethyl)hexanoate* (**16b**). 0.17 g (52%) as a yellow oil. ^1^H-NMR (CDCl_3_): δ 4.05 (q, *J* = 7.2 Hz, 2H), 2.44–2.32 (m, 3H), 2.24–2.11 (m, 5H), 2.05–1.99 (m, 1H), 1.68–1.57 (m, 1H), 1.52–1.46 (m, 4H), 1.39–1.32 (m, 2H), 1.19 (t, *J* = 7.2 Hz, 3H), 1.11–0.97 (m, 2H), 0.81 (dd, *J* = 6.5, 5.3 Hz, 6H). ^13^C-NMR (CDCl_3_) δ 173.85, 64.54, 60.04, 55.21 (2C), 43.00, 39.25, 30.97, 26.23 (2C), 25.52, 24.69, 22.95 (2C), 14.40. HRMS (FAB+): *m*/*z* calcd for C_15_H_29_NO_2_: 255.2198, found (M + 1): 256.2267.

*Ethyl 5-methyl-3-((3-methylpiperidin-1-yl)methyl)hexanoate* (**16c**). 0.09 g (20%) as an amber oil. ^1^H-NMR (CDCl_3_): δ 4.09 (q, *J* = 7.1 Hz, 2H), 2.84–2.76 (m, 1H), 2.68–2.61 (m, 1H), 2.38–2.32 (m, 1H), 2.21–2.09 (m, 3H), 2.08–1.99 (m, 1H), 1.92–1.85 (m, 1H), 1.71–1.36 (m, 6H), 1.24 (t, *J* = 7.1 Hz, 3H), 1.17–1.02 (m, 2H), 0.88–0.81 (m, 10H). ^13^C-NMR (CDCl_3_): δ 173.79 (C-3), 64.31 (C-6), 64.27 (C-6), 63.69 (C-7), 62.03 (C-7), 60.01 (C-2), 55.52 (C-12), 53.90 (C-12), 43.10 (C-13), 43.07 (C-13), 39.21 (C-4), 33.33 (C-10), 31.41 (C-8), 31.13 (C-5), 25.80 (C-11), 25.70 (C-11), 25.54 (C-14), 23.03(C-15), 22.97 (C-16), 19.83 (C-9), 19.81 (C-9), 14.40 (C-1). HRMS (FAB+): *m*/*z* calcd for C_16_H_31_NO_2_: 269.2355, found (M + 1): 270.2411.

*Ethyl 5-methyl-3-((4-methylpiperidin-1-yl)methyl)hexanoate* (**16d**). 0.164 g (37%) as a yellow oil. ^1^H-NMR (CDCl_3_): δ 4.12 (q, *J* = 7.2 Hz, 2H), 2.87 (d, *J* = 11.3 Hz, 1H), 2.71 (d, *J* = 11.8 Hz, 1H), 2.36 (dd, *J* = 5.7 Hz, 1H), 2.18–2.11 (m, 2H), 2.07 (m, 1H), 1.96 (ddd, *J* = 11.5,, 11.5, 2.5 Hz), 1.74 (ddd, *J* = 11.6, 2.4 Hz, 1H), 1.64 (m, *J* = 6.4 Hz, 1H), 1.57–1.53 (m, 2H), 1.26 (t, *J* = 7.1 Hz, 4H), 1.20–1.03 (m, 4H), 0.91–0.85 (m, 9H). ^13^C-NMR (CDCl_3_) δ 173.89, 64.13, 60.06, 55.56, 53.67, 42.98, 39.27, 34.78, 34.52, 31.05, 31.03, 25.44, 22.95 (2C), 22.13, 14.41. HRMS (FAB+): *m*/*z* calcd for C_16_H_31_NO_2_: 269.2355, found (M + 1): 270.2429.

*Ethyl 5-methyl-3-(morpholinomethyl)hexanoate* (**16e**). 0.166 g (37%) as a yellow oil. ^1^H-NMR (CDCl_3_): δ 4.13 (q, *J* = 7.2 Hz, 2H), 3.71–3.59 (m, 4H), 2.50 (m, 2H), 2.34 (m, 3H), 2.26–2.16 (m, 3H), 2.13–2.06 (m, 1H), 1.71–1.59 (m, 1H), 1.26 (t, *J* = 7.1 Hz, 3H), 1.20–1.06 (m, 2H), 0.89 (d, *J* = 6.3 Hz, 6H). ^13^C-NMR (CDCl_3_): δ 173.71, 67.23 (2C), 64.25, 60.21, 54.16 (2C), 42.82, 39.09, 30.42, 25.41, 22.96, 22.86, 14.40. HRMS (FAB+): *m*/*z* calcd for C_14_H_27_NO_3_: 257.1991, found (M + 1): 258. 2077.

*Ethyl 5-methyl-3-(thiomorpholinomethyl)hexanoate* (**16f**). 0.11 g (25%) as a yellow oil. ^1^H-NMR (CDCl_3_): δ 4.17–4.07 (m, 2H), 2.78–2.71 (m, 2H), 2.63–2.53 (m, 6H), 2.32–2.24 (m, 2H), 2.2–2.13 (m, 2H), 2.04 (dd, *J* = 12.2, 9.0 Hz, 1H), 1.69–1.57 (m, 1H), 1.27 (t, *J* = 7.2 Hz, 3H), 1.16–1.03 (m, 2H), 0.88 (d, *J* = 6.2 Hz, 6H). ^13^C-NMR (CDCl_3_): δ 173.76, 64.60, 60.22, 55.75, 42.82, 39.15, 30.87, 28.18, 25.47, 23.03, 22.91, 14.46. HRMS (FAB+): *m*/*z* calcd for C_14_H_27_NO_3_S: 273.1762, found (M + 1): 274.1858.

*Ethyl 3-(4-chlorophenyl)-4-(piperidin-1-yl)butanoate* (**18b**). 0.2 g (52%) as a yellow oil. ^1^H-NMR (CDCl_3_): δ 7.25 (d, *J* = 8.5 Hz, 2H) 7.13 (d, *J* = 8.5 Hz, 2H), 4.03 (q, *J* = 7.1 Hz, 2H), 3.35 (ddd, *J* = 11.9, 9.1, 6.0 Hz, 1H), 2.87 (dd, *J* = 15.5, 6.3 Hz, 1H), 2.49–2.41 (m, 3H), 2.41–2.32 (m, 2H), 2.31–2.23 (m, 2H), 1.55–1.46 (m, 4H), 1.42–1.38 (m, 2H), 1.16 (t, *J* = 7.1 Hz, 3H). ^13^C-NMR (CDCl_3_) δ 172.57, 141.72, 132.11, 128.90 (2C), 128.47 (2C), 65.17, 60.19, 54.87 (2C), 39.25 (2C), 26.00, 24.34, 14.12. HRMS (FAB+): *m*/*z* calcd for C_17_H_24_ClNO_2_: 309.1496, found (M + 1): 310.1556.

*Ethyl 3-(4-chlorophenyl)-4-(3-methylpiperidin-1-yl)butanoate* (**18c**). 0.22 g (49%) as a yellow oil. ^1^H-NMR (CDCl_3_): δ 7.24 (d, *J* = 8.5 Hz, 2H), 7.13 (d, *J* = 8.5 Hz, 2H), 4.02 (q, *J* = 7.1 Hz, 2H), 3.42–3.32 (m, 1H), 2.86 (dd, *J* = 15.5, 6.3 Hz, 2H), 2.69 (d, *J*= 8.8 Hz, 1H), 2.49–2.39 (m, 2H), 2.35 (dd, *J* = 12.6, 5.7 Hz, 1H), 1.96 (ddd, *J* = 11.2, 11.2, 3.0 Hz, 1H), 1.75 (ddd, *J* = 11.4, 11.4, 2.7 Hz, 1H), 1.71–1.39 (m, 5H), 1.15 (t, *J* = 7.1 Hz, 3H), 0.83 (t, *J* = 6.2 Hz, 4H). ^13^C-NMR (CDCl_3_): δ 172.66 (C-3), 141.66 (C-13), 132.14 (C-16), 128.90 (C-15), 128.48 (C-14), 64.93 (C-6), 64.90 (C-6), 63.35 (C-7), 61.42 (C-7), 60.25 (C-2), 55.28 (C-12), 53.40 (C-12), 39.32 (C-5), 39.24 (C-4), 32.92 (C-10), 31.14 (C-8), 30.87 (C-8), 25.52 (C-11), 25.38 (11), 19.67 (C-9), 19.63 (C-9), 14.10 (C-1). HRMS (FAB+): *m*/*z* calculado para C_18_H_26_ClNO_2_: 323.1652, experimental (M + 1): 324.1734.

*Ethyl 3-(4-chlorophenyl)-4-(4-methylpiperidin-1-yl) butanoate* (**18d**). 0.75 g (49%) as a yellow oil. ^1^H-NMR (CDCl_3_): δ 7.24 (d, *J* = 8.5 Hz, 2H), 7.13 (d, *J* = 8.5 Hz, 2H), 4.03 (q, *J* = 7.1 Hz, 2H), 3.39–3.31 (m, 1H), 2.94–2.89 (d, *J* = 11.1 Hz, 1H), 2.85 (dd, *J* = 15.5, 6.3 Hz, 1H), 2.74 (d, *J* = 11.1 Hz, 1H), 2.50–2.41 (m, 2H), 2.36 (dd, *J* = 12.6, 5.7 Hz, 1H), 2.03 (ddd, *J* = 11.1, 11.1, 2.5 Hz, 1H), 1.83 (ddd, *J* = 11.1, 11.1, 2.5 Hz, 1H), 1.59–1.52 (m, 2H), 1.35–1.24 (m, 1H), 1.15 (t, *J* = 7.1 Hz, 6H), 0.89 (d, *J* = 6.4 Hz, 3H). ^13^C-NMR (CDCl_3_) δ 172.36, 141.75, 132.06, 128.88 (2C), 128.42 (2C), 64.76, 60.04, 55.34, 53.18, 39.38, 39.18, 34.55, 34.28, 30.71, 21.90, 14.11. HRMS (FAB+): *m*/*z* calcd for C_18_H_26_ClNO_2_: 323.1652, found (M + 1): 324.1772.

*Ethyl 3-(4-chlorophenyl)-4-morpholinobutanoate* (**18e**). 0.645 g (55 %) as a yellow oil. ^1^H-NMR (CDCl_3_): δ 7.25 (d, *J* = 8.4 Hz, 2H), 7.13 (d, *J* = 8.4 Hz, 2H), 4.05 (q, *J* = 7.1 Hz, 2H), 3.67–3.59 (m, 4H), 3.43–3.32 (m, 1H), 2.85 (dd, *J* = 15.5, 6.6 Hz, 1H), 2.58–2.30 (m, 7H), 1.16 (t, *J* = 7.1 Hz, 3H). ^13^C-NMR (CDCl_3_) δ 172.25, 141.22, 132.21, 128.82 (2C), 128.50 (2C), 66.89 (2C), 64.71, 60.20, 53.79 (2C), 39.12, 38.67, 14.09. HRMS (FAB+): *m*/*z* calcd for C_16_H_22_ClNO_3_: 311.1288, found (M + 1): 312.1299.

*Ethyl 3-(4-chlorophenyl)-4-thiomorpholinobutanoate* (**18f**). 0.66 g (58 %) as a yellow oil. ^1^H-NMR (CDCl_3_): δ 7.25 (d, *J* = 8.5 Hz, 1H), 7.12 (d, *J* = 8.4 Hz, 1H), 4.05 (q, *J* = 7.1 Hz, 2H), 3.40–3.30 (m, 1H, H-5), 2.83–2.77 (m, 3H), 2.66–2.56 (m, 6H), 2.5–2.39 (m, 3H), 1.18 (t, *J* = 7.1 Hz, 3H). ^13^C-NMR (CDCl_3_): δ 172.42, 141.23, 132.29, 128.86 (2C), 128.55 (2C), 65.06, 60.34, 55.39 (2C), 39.13 (2C), 27.92 (2C), 14.14. HRMS (FAB+): *m*/*z* calcd for C_16_H_22_ClNO_2_S: 327.106, found (M + 1): 328.1084.

#### 3.2.6. General Procedure for the Hydrolysis of Esters **16a**–**f** and **18b**–**f**

The corresponding ethyl ester **16a**–**f** or **18b**–**f** (1.0 equiv) were dissolved in MeOH (6.0 mL), and NaOH (1.1 equiv) dissolved in water (2 mL) was added dropwise. The reaction was monitored until the consumption of the starting materials (tlc). When the reaction was complete, the solvent was evaporated and aqueous HCl was added until the mixture reached a pH = 5. The mixture was extracted with ethyl acetate (3 × 5 mL), dried over Na_2_SO_4_ and the solvent removed in vacuo.

*5-Methyl-3-(thiazolidin-3-ylmethyl)hexanoic acid* (**8a**). 0.055 g (77%) as a yellow oil. ^1^H-NMR (CD_3_OD): δ 3.83 (s, 2H), 3.51–3.42 (m, 1H), 3.23 (m, 2H), 3.10–2.68 (m, 3H), 2.56–2.44 (m, 1H), 2.37–2.18 (m, 2H), 1.74–1.63 (m, 1H), 1.22–1.12 (m, 2H), 0.94–0.91 (m, 6H). ^13^C-NMR (CD_3_OD): δ 180.44, 60.81, 59.41, 51.43, 43.92, 39.09, 31.45, 30.39, 26.31, 23.36, 22.65.

*5-Methyl-3-(piperidin-1-ylmethyl)hexanoic acid* (**8b**). 0.55 g (90%) as a yellow oil. ^1^H-NMR (CD_3_OD): δ 3.04–2.99 (m, 1H), 2.85 (dd, *J* = 13.2, 10.4 Hz, 1H), 2.52 (dt, *J* = 16.6, 1.8 Hz, 1H), 2.35 (dd, *J* = 16.6, 10.3 Hz, 1H), 2.20 (m, 1H), 1.86 (s, 5H), 1.74–1.63 (m, 2H), 1.14 (dd, *J* = 7.2, 7.2 Hz, 2H), 0.935 (d, *J* = 4 Hz, 3H), 0.925 (d, *J* = 4 Hz, 3H). ^13^C-NMR (CD_3_OD) δ 181.03, 65.16 (3C), 44.96, 44.16, 30.36, 26.11, 24.76 (2C), 23.00, 22.96, 22.77. HRMS (FAB+): *m*/*z* calcd for C_13_H_25_NO_2_: 227.1885, found (M + 1): 228.1988.

*5-Methyl-3-((3-methylpiperidin-1-yl)methyl)hexanoic acid* (**8c**). 0.04 g (70%) as a yellow oil. ^1^H-NMR (CD_3_OD): δ 3.61–3.47 (m, 2H), 3.28–3.26 (m, 1H), 3.04 (d, *J* = 13.2 Hz, 1H), 2.94–2.85 (m, 2H), 2.74–2.59 (m, 1H), 2.53 (dd, *J* = 16.7 Hz, 1H), 2.36 (dd, *J* = 16.6, 10.2 Hz, 1H), 2.27–2.17 (m, 1H), 1.94–1.88 (m, 2H), 1.88–1.77 (m, 2H), 1.73–1.64 (m, 1H), 1.15 (dd, *J* = 7.2, 7.2 Hz, 2H), 1.02–0.99 (m, 3H), 0.95–0.92 (m, 6H). ^13^C-NMR (CD_3_OD): δ 180.92 (C-1), 65.05 (C-4), 59.38 (C-5), 53.18 (C-10), 44.79 (C-2), 44.70 (C-2), 44.15 (C-11), 44.13 (C-11), 31.54 (C-8), 30.37 (C-6, C-9), 30.26 (C-3), 26.11 (C-12), 23.05 (C-15), 22.99 (C-15), 22.80 (C-16), 22.77 (C-16), 18.95 (C-7). δ HRMS (FAB+): *m*/*z* calcd for C_14_H_27_NO_2_: 241.2042, found (M + 1): 242.2106.

*5-Methyl-3-((4-methylpiperidin-1-yl)methyl)hexanoic acid* (**8d**). 0.1 g (90%) as a yellow oil. ^1^H-NMR (CD_3_OD): δ 3.64–3.56 (m, 2H), 3.04–3.00 (m, 1H), 2.88 (dd, *J* = 13.4, 10.3 Hz, 1H), 2.57–2.48 (m, 1H), 2.35 (dd, *J* = 16.8, 10.3 Hz, 1H), 2.25–2.14 (m, 1H), 1.91–1.82 (m, 2H), 1.68 (m, 1H), 1.53–1.48 (m, 3H), 1.14 (ddd, *J* = 7.3 Hz, 2H), 1.01 (d, *J* = 6.7 Hz, 3H), 0.93 (d, *J* = 6.7 Hz, 6H). ^13^C-NMR (CD_3_OD) δ 180.86, 64.63, 53.21 (2C), 44.75, 44.07, 32.56 (2C), 30.37, 28.50, 26.05, 22.99, 22.76, 21.24. HRMS (FAB+): *m*/*z* calcd for C_14_H_27_NO_2_: 241.2042, found (M + 1): 242.2121.

*5-Methyl-3-(morpholinomethyl)hexanoic acid* (**8e**). 0.1 g (85%) as a yellow oil. ^1^H-NMR (CD_3_OD): δ 3.89–3.87 (m, 4H), 3.29–3.24 (m, 2H), 3.12–3.06 (m, 2H), 3.05–3.00 (m, 1H), 2.88 (dd, *J* = 13.1, 10.2 Hz), 2.54–2.48 (m, 1H), 2.41 (dd, *J* = 16.6, 9.6 Hz, 1H), 2.29–2.18 (m, 1H), 1.75–1.62 (m, 1H), 1.16 (dd, *J* = 3.9, 3.9 Hz, 2H), 0.93 (d, *J* =4.8 Hz, 3H), 0.92 (d, *J* =4.8 Hz, 3H). ^13^C-NMR (CD_3_OD) δ 180.28, 65.80 (2C), 64.92, 53.58 (2C), 43.95 (2C), 29.94, 26.12, 23.03, 22.79. HRMS (FAB+): *m*/*z* calcd for C_14_H_27_NO_2_: 229.1678, found (M + 1): 230.1743.

*5-Methyl-3-(thiomorpholinomethyl)hexanoic acid* (**8f**). 0.067 g (90%) as a yellow oil. ^1^H-NMR (CD_3_OD): δ 3.51–3.44 (m, 2H), 3.35–3.29 (m, 2H), 3.11–3.02 (m, 1H), 2.95–2.92 (m, 4H), 2.88–2.80 (m, 1H), 2.50 (d, *J* = 16.6 Hz, 1H), 2.39 (dd, *J* = 16.6, 10.0 Hz, 1H), 2.25–2.16 (m, 1H), 1.68 (sept, *J* = 8 Hz, 1H), 1.14 (dd, *J* = 7.2, 7.2 Hz, 2H), 0.92 (d, *J* = 5.2 Hz, 3H), 0.9 (d, *J* = 5.2 Hz, 3H). ^13^C-NMR (CD_3_OD) δ 179.55, 64.35, 54.31 (2C), 43.16, 42.58, 28.83, 25.24 (2C), 24.71, 21.72, 21.41. HRMS (FAB+): *m*/*z* calcd for C_14_H_27_NO_2_: 245.1449, found (M + 1): 246.1524.

*3-(4-Chlorophenyl)-4-(piperidin-1-yl)butanoic acid* (**9b**). 0.3 g (85%) of a white solid, m.p = 146–149 °C. ^1^H-NMR (CD_3_OD): δ 7.34 (d, *J* = 8.6 Hz, 2H), 7.27 (d, *J* = 8.6 Hz, 2H), 3.52 (dddd, *J* = 8 Hz, 8 Hz, 4 Hz, 4 Hz, 1H), 3.37 (dd, *J* = 14, 8 Hz, 3H), 3.11 (ddd, *J* = 13.0, 2.0, 1.2 Hz, 3H), 2.91 (dd, *J* = 16.7, 5.2 Hz, 1H), 2.63 (ddd, *J* = 13.6, 13, 1.2 Hz, 1H), 1.89–1.82 (m, 4H), 1.64 (sa, 2H). ^13^C-NMR (CD_3_OD) δ 179.28, 142.37, 133.06, 130.11 (2C), 129.81 (2C), 64.71, 54.68, 45.63, 38.12, 24.62 (2C), 22.93. HRMS (FAB+): *m*/*z* calcd for C_15_H_20_ClNO_2_: 281.1183, found (M + 1): 282.1287.

*3-(4-Chlorophenyl)-4-(3-methylpiperidin-1-yl)butanoic acid* (**9c**). 0.2 g (52%) as a yellow oil. ^1^H-NMR (CD_3_OD): δ 7.37–7.26 (m, 4H), 3.76 (d, *J* = 11.5 Hz, 1H), 3.68 (d, *J* = 11.8 Hz, 1H), 3.58–3.47 (m, 1H), 3.43–3.36 (m, 2H), 3.08 (d, *J* = 13.0 Hz, 1H), 2.93 (dd, *J* = 16.7, 10.8 Hz, 1H), 2.70–2.55 (m, 2H), 2.41 (t, *J* = 11.7 Hz, 1H), 1.95–1.79 (m, 4H), 1.21–1.09 (m, 1H), 1.01 (d, *J* = 6.7 Hz, 1H), 0.99 (d, *J* = 6.7 Hz, 2H). ^13^C-NMR (CD_3_OD): δ 179.60 (C-1), 179.57 (C-1), 142.62(C-11), 134.13 (C-14), 130.23 (C-13), 129.97 (C-12), 64.91(C-4), 64.81 (C-4), 61.19 (C-5), 59.66 (C-5), 54.89 (C-10), 53.48 (C-10), 45.93 (C-2), 38.34 (C-3), 38.19 (C-3), 31.67 (C-8), 31.21 (C-6), 30.85 (C-6), 24.39 (C-9), 24.27 (C-9), 19.13 (C-7), 19.11 (C-7). HRMS (FAB+): *m*/*z* calcd for C_16_H_22_ClNO_2_: 295.1339, found (M + 1): 296.1445.

*3-(4-Chlorophenyl)-4-(4-methylpiperidin-1-yl)butanoic acid* (**9d**). 0.26 g (68%) of a yellow solid, m.p = 142–144 °C. ^1^H-NMR (CD_3_OD): δ 7.34 (d, *J* = 8.6 Hz, 1H), 7.27 (d, *J* = 8.6 Hz, 1H), 3.77 (d, *J* = 11.7 Hz, 1H), 3.54–3.49 (m, 1H), 3.47–3.34 (m, 2H), 3.08 (d, *J* = 12.9 Hz, 1H), 3.01–2.95 (m, 1H), 2.93 (dd, *J* = 16, 16 Hz, 1H), 2.78 (dd, *J* = 11.4, 11.4 Hz, 1H), 2.62 (dd, *J* = 16, 4 Hz,1H), 1.94–1.85 (m, 2H), 1.69 (m, 1H), 1.59–1.45 (m, 2H), 1.01 (d, *J* = 6.5 Hz, 3H). ^13^C-NMR (CD_3_OD) δ 179.75, 142.61, 134.00, 130.10 (2C), 129.74 (2C), 64.59, 54.86, 53.36, 46.09, 38.25, 32.84, 32.57, 29.94, 21.22. HRMS (FAB+): *m*/*z* calcd for C_16_H_22_ClNO_2_: 295.1339, found (M + 1): 296.1445

*3-(4-Chlorophenyl)-4-morpholinobutanoic acid* (**9e**). 0.28 g (69%) of a yellow solid, m.p = 166–168 °C. ^1^H-NMR (CD_3_OD): δ 7.32 (d, *J* = 8.5 Hz, 2H), 7.25 (d, *J* = 8.5 Hz, 2H), 3.81–3.78 (m, 4H), 3.5–3.43 (dddd, *J* = 4.8, 4.8, 4, 4 Hz, 1H), 3.08–2.98 (m, 3H), 2.94–2.81 (m, 4H), 2.57 (dd, *J* = 16.2, 4.5 Hz, 1H). ^13^C-NMR (CD_3_OD) δ 178.35, 142.88, 133.90, 130.13 (2C), 130.00 (2C), 66.77 (2C), 65.41, 54.26 (2C), 43.60 (1C), 38.88. HRMS (FAB+): *m*/*z* calcd for C_16_H_22_ClNO_2_: 283.0975, found (M + 1): 284.1009.

*3-(4-Chlorophenyl)-4-thiomorpholinobutanoic acid* (**9f**). 0.288 g (70%) of a white solid, m.p = 144–146 °C. ^1^H-NMR (CD_3_OD): δ 7.32 (d, *J* = 8 Hz, 2H), 7.25 (d, *J* = 8 Hz, 2H), 3.53–3.44 (m, 1H), 3.37–3.33 (m, 2H), 3.17–3.06 (m, 3H), 2.97–2.85 (m, 2H), 2.87–2.85 (m, 4H), 2.58 (dd, *J* = 16.3, 3.8 Hz, 1H). ^13^C-NMR (CD_3_OD) δ 178.90, 142.77, 133.79, 129.92 (2C), 129.89 (2C), 65.79, 55.95 (2C), 44.32, 38.78, 27.23 (2C). HRMS (FAB+): *m*/*z* calcd for C_16_H_22_ClNO_2_: 299.0747, found (M + 1): 300.0811.

### 3.3. Enzyme Inhibitory Tests

The enzymatic reactions were carried out at final concentrations of 0.2 mM of α-ketoglutarate, 1.25 mM of β-NADP and 0.0209 mg/mL of GABA-AT from *Pseudomonas fluorescens* in a 0.1 M potassium pyrophosphate buffer solution, pH = 8.6, at 25 °C for 30 min. Once the incubation period was over, the β-NADPH absorbance was measured at λ_max_ = 340 nm. The experiments were carried out at final equimolar concentrations of 0.8 mM for compounds **7a**–**f**, **8a**–**f** and **9b**–**f**, VGB and VPNa versus 0.8 mM of GABA.

### 3.4. In Vivo Tests

All experiments were carried out on male CD1 mice with body weights between 20–26 g, which were kept under the same conditions of *ad libitum* feeding, natural lightness-darkness cycle and a temperature at 22 °C and four days of adaptation to laboratory conditions. The test compounds and reference drug (sodium valproate; VPNa) were injected i.p. with volume injection at 1.0 mL/100 g weight to groups between 6–9 mice [63,64,65,66] at 0.50 and 1.00 mmole/kg for **9b** and VPNa. Another group between 6–9 mice served as control, which received the vehicle (phosphate buffer, C = 0.1 M at pH = 8.0). The assays were carried out at 1 h and 4 h of pretreatment. 1 h or 4 h after, 75 mg/kg of PTZ were injected subcutaneously with volume injection at 1.0 mL/100 g weight. Each animal was placed into an individual plastic cage for observation lasting 1 h. Seizures, tonic-clonic convulsions, death and latency were recorded. At least 80% of the animals in the control group showed convulsions.

### 3.5. Computational Details

#### 3.5.1. Conformational Analysis and Geometry Optimization

All the molecules in the study (in their neutral and zwitterionic form) were submitted to a conformational analysis within the molecular mechanics level of theory employing the SYBYL force field [67]. For geometry optimization semi-empirical calculations using the parametric method number 3 (PM3) [68] over all the equilibrium conformers of the GABA derivatives were performed. A further optimization within the density functional theory approach, employing the hybrid functional B3LYP [69] and the 6–31*G basis set [70] was carried out. A frequency analysis was carried out to corroborate that we were in a minimum in the potential energy surface. All the calculations were performed in the SPARTAN 08 program [71]. Mulliken electrostatic partial charges were obtained to be used further as molecular quantum descriptors.

#### 3.5.2. Molecular Descriptors Calculations

We obtained a total of 209 molecular descriptors derived from the constitutional, topological, geometrical, functional groups counts, molecular charge and molecular properties families of descriptors (Table 8). The selection of these molecular descriptor families was based on finding the structural modifications (functional groups, form, charge distribution and physicochemical properties) over the GABA scaffold that improves its potency as GABA-AT inhibitors. For these calculations the DRAGON 05 software was employed [72].

#### 3.5.3. QSAR Model Construction and Validation

Mathematical model construction was carried out with the genetic algorithms technique (GA) in the MobyDigs 01 program [73]. All the molecular descriptors of the GABA analogues were used as the independent variables (X) and percent inhibitory value as the dependent variable (Y). QSAR model selection was based on its statistical parameters ($R^{2}$, $Q^{2}$, s, and F) values and that it passed the QUIK, REDUNDANCY, OVERFITTING and $Q_{\mathrm{ASYMPTOTIC}}^{2}$ rules. The final QSAR model was validated with a test group that consist of 20% of all the molecules in this study, these molecules were randomly selected. This procedure was done a minimum of ten times.

The QUIK rule allows the rejection of models with high molecular descriptors co-linearity that can lead to casual correlation [74]. This rule is based on the K multivariate correlation index that measures the total correlation of a set of variables, defined as:

(4) $$\;K=\;\frac{\sum_{j}\left[\frac{\lambda_{j}}{\sum_{j}\lambda_{j}}-\frac{1}{p}\right]}{\frac{2\left(p-1\right)}{p}}\;j=1,\ldots ,p\;and\;0\leq K\leq 1\;$$

where $\lambda_{j}$ is the data from the joint X(n, p) matrix eigenvalues, n is the number of objects and p is the number of variables in the model. The total correlation in the set given by the descriptors X plus the response Y ($K_{XY}$ ) should always be greater than that measured only in the set of descriptors ($K_{X}$); if $K_{XY}-K_{X}<\Delta K$, the model is rejected; ΔK may have values between 0.0 and 0.05.

The goal of the OVERFITTING rule is to detect models with an excess of “bad” molecular descriptors. This rule stipulates that: if $R^{N}<t^{N}\left(\varepsilon \right)$ the model is rejected. The $t^{N}\left(\varepsilon \right)$ values are calculated by the following equation:

(5) $$\;\;t^{N}\left(\varepsilon \right)=\frac{p.\varepsilon -R}{p.R}\;\;$$

where p is the number of descriptors in the model and ε values range from 0.01 to 0.1. For this study ε=0.01. $R^{N}$ is defined by:

(6) $$\;R^{N}=\overset{P^{-}}{\underset{j=1}{\sum }}M_{j}\;M_{j}<0\;and\;-1\leq R^{N}\geq 0\;$$

where $M_{j}$ is defined by the equation:

(7) $$\;M_{j}=\frac{R_{jY}}{p}-\frac{1}{p}\;-\frac{1}{p}\leq M_{j}\geq \frac{p-1}{p}\;$$

$R_{jY}$ is the absolute value of the regression coefficient between the *j*-th descriptor and the response Y. The parameter $R^{N}$ accounts for an excess of noisy or useless variables and can be thought as a measure of overfitting. It takes the maximum value equal to zero when non-noisy variables are in the model.

As mentioned above, to test our model more rigorously in terms of the predictive aspect, an evaluation of the estimated error was performed on all the molecules that were excluded from the training process. To evaluate the internal predictive ability of our models, the leave one out technique was employed ($Q_{LOO}^{2}$). This technique consists of evaluating each molecule within the training set to determine as to how it corresponds to the validation set [75]. Each individual molecule is evaluated, and its Y value is predicted:

(8)  QLOO2=1− ∑i=1n(yi− y^ii)2∑i=1n(yi− y¯)2 

Another form to evaluate the predictive ability of our mathematical model is by the asymptotic squared Q rule ($Q_{\mathrm{ASYMPTOTIC}}^{2}$ ) which states that a model is predictive if $Q_{LOO}^{2}-Q_{ASYM}^{2}>\Delta Q$, where ΔQ values range from −0.005 up to 0.005. $Q_{ASYM}^{2}$ is defined as:

(9) $$\;Q_{ASYM}^{2}=1-\;\left(1-\;R^{2}\right).{\left(\frac{n}{n-\overset{´}{p}}\right)}^{2}\;$$

where n is the number of objects and $\overset{´}{p}$ is the number of variables in the model.

In addition, the standard deviation (s) and Fisher test (F) values were calculated, where the s parameter contains information about the correlation between the experimental and calculated activities and the number of molecules in the study.

(10) $$\;s=\;\frac{\sum_{i=1}^{n}{\left({\hat{y}}_{i}-\;y_{i}\right)}^{2}}{n-2}\;$$

n represents the number of molecules in the study, ${\hat{y}}_{i}$ represents the experimental activity and $y_{i}$ is the calculated activity from the QSAR model.

Meanwhile, parameter, *F* is related to the probability for the mathematical model to casually occur:

(11)  F= ∑i=1n(y^i− y¯)2dfM∑i=1n(y^i− yi)2dfE  

$df_{M}$ and $df_{E}$ refer to the molecular descriptors of the model and error, respectively. Also, ${\hat{y}}_{i}$, $\bar{y}$ and $y_{i}$ refer to the experimental, average value of experimental and calculated activities, respectively. In the mathematical model, s and F should have the smallest and largest possible values, respectively, to ensure that the QSAR model is reliable [76].

#### 3.5.4. Homology Structural Modeling and Refinement

Given that a full 3D structure of the GABA aminotransferase of human and *P. fluorencens* is unavailable, we generated homology structural models. We used GABA aminotransferase structures available in the Protein Data Bank server (PDB) as structural templates (Table 9) with the MODELLER 9.18 program [77]. According to the sequence alignment of GABA aminotransferases (Figure S1), those of bacteria are similar amongst themselves, and the same is true for animal GABA aminotransferases (human and wild boar).

For the model of human GABA-AT we used the crystallographic structure 4y0h of wild boar, on account of its resolution (1.63 Å) and a higher sequence identity (~95%) to the human enzyme. The chains A and B were used to build the monomers. Once done, the dimer was assembled based in the same structure of 4y0h.

The model of *P. fluorencens* GABA-AT was performed using the 1sf2 crystallographic structure of *E. coli*. We used this structure instead of the *M. abscessus*, due a better conservation with respect of *E. coli* aminotransferase (~74% of identity). Again, the A and B chains were used to build the monomers and the dimer was assembled according to the 1sf2 structure.

In the case of the bacterial GABA aminotransferases, both crystallographic structures of *Mycobacterium* and *E. coli* are tetrameric structures instead of dimers as in animals; however, the catalytic site is in the interface of each dimer instead of tetramer interface, suggesting that dimer is the minimal catalytic form. This agrees with experimental studies suggesting that the dimer of bacteria is functional [27]; therefore, we used the bacterial dimer in this work.

Finally, to optimize the complete structures of GABA-AT models, we used the CHARMM36 force field within CHARMM38b1 [78] to add hydrogen atoms and carry out a 100 step steepest decent minimization fixing all heavy atoms, followed by a conjugate gradient minimization of the full structure to remove bad contacts and clashes. These last protein conformations obtained were employed to carry out the molecular docking calculations.

To incorporate the prosthetic group (PLP) in the homology models an alignment employing a crystal structure that possessed the PLP was done (3r4t and 1ohw for the *Pseudomonas* and *human* models respectively). Additionally, from this alignment the Fe_2_/S_2_ (inorganic) cluster, present in the crystal structures of *wild boar* as a cofactor, was added to the human GABA-AT model.

#### 3.5.5. Molecular Docking Calculations

Molecular docking calculations of all the GABA analogues (including *R* & *S* enantiomers for molecules of series 2 and 3) over *Pseudomonas* and *human* GABA-AT structures, generated in the homology modeling process, were done in Molegro Virtual Docker (MVD) 6.0 software (Redwood, CA, USA) [79,80]. The cavities were detected by the expanded Van der Waals sphere method. The calculations were performed over the catalytic site with a cavity volume of 137.73 Å^3^ and 339.46 Å^3^ for the *Pseudomonas* and *human* model respectively. The cavity selection was based on the results of the high molecular similarity with the endogenous ligand. We employed the minimum energy geometries of all the GABA derivatives in their zwitterionic form, and the Mulliken partial charges obtained by the DFT calculations. The residues within 6 Å were set as flexible, 2000 minimization steps for each flexible residue and 2000 steps of global minimization per run were set. The MolDock Optimizer search function, based on an evolutionary algorithm, was used. A total of 20 runs with a maximum of 4000 iterations using a population of 200 individuals (for the *Pseudomonas* model) and 400 individuals (for the *human* model) per run were set. To calculate the interaction energy, we used the scoring function Moldock Score [GRID]. The scoring function GRID was set at 0.2 Å and the search sphere fixed as 15 Å radius. For the energy analysis of the ligand, the electrostatic internal interactions, internal hydrogen bonds and the sp^2^-sp^2^ torsions were considered. The method was validated by reproducing the experimental binding mode of the reference inhibitor within the **3r4t** crystal structure to validate the *Pseudomonas* model, and **1ohw** and **1ohy** to validate the *human* model, with a Root Media Square Deviation (RMSD) of 1.7 Å, 1.3 Å and 1.8 Å respectively (Figures S2–S4, see Supplementary Material).

## 4. Conclusions

The synthesis of a new series of GABA analogues **7**, **8** and **9** substituted at the γ-position with thiazolidine, piperidine, morpholine and thiomorpholine heterocyclic ring systems, was accomplished by short and efficient synthetic routes. The chemical modifications achieved by the attachment of these heterocyclic moieties to the GABA backbone, led to compounds with GABA-AT inhibitory activity. As compared with the positive control compounds VGB or VPNa, enzymatic inhibition tests carried out with all analogues, showed that **9b** display a 73% inhibition over the GABA-AT enzyme. In vivo studies carried out with male CD1 mice show that **9b** has a dose-dependent neuroprotective effect. In the number of seizures generated during the observation time, there is a decrease in the number of seizures at a 0.5 mmole/kg dose. However, at a 1.00 mmole/kg dose, **9b** has no protecting effect.

Our QSAR model allowed us to find the structural parameters of the GABA analogues that enhance their inhibitory activity over GABA-AT. Molecular docking results confirmed the descriptive ability of our QSAR model by correlating GABA derivatives molecular descriptors with the interaction elements that are key to bind into GABA-AT. These results suggest that compound **9b** is a good candidate for further studies. Finally, these methods can be used for the design and further development of new reversible GABA-AT competitive inhibitors.

## Figures and Tables

**Table 1:** Synthesis of analogues **7**.

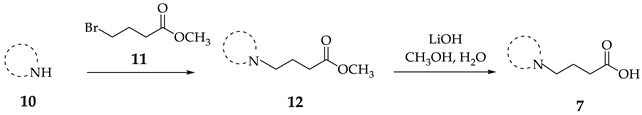			
Compound	Heterocycle	% Yield 12	% Yield 7
**7a**	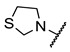	37	81
**7b**	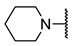	67	95
**7c**	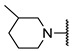	60	95
**7d**	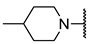	67	95
**7e**	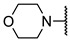	52	98
**7f**	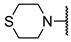	58	86

**Table 2:** Synthesis of conjugated esters **14**.

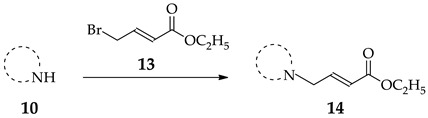		
Compound	Heterocycle	% Yield 14
**10a**	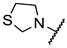	63
**10b**	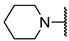	90
**10c**	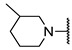	77
**10d**	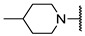	80
**10e**	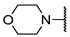	89
**10f**	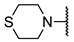	80

**Table 3:** Synthesis of the β-substituted analogues **8**.

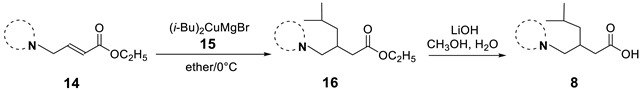				
Entries	Compound	Heterocycle	% Yield 16	% Yield 8
**a**	**8a**	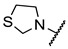	13	77
**b**	**8b**	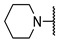	52	90
**c**	**8c**	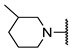	20	70
**d**	**8d**	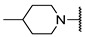	37	90
**e**	**8e**	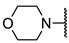	37	85
**f**	**8f**	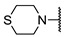	25	90

**Table 4:** Synthesis of the β-substituted analogues **9**.

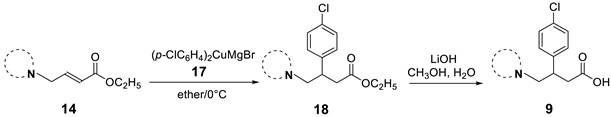				
Entries	Compound	Heterocycle	% Yield 18	% Yield 9
**a**	**9a**	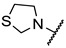	--	--
**b**	**9b**	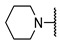	52	85
**c**	**9c**	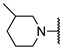	49	52
**d**	**9d**	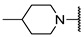	49	68
**e**	**9e**	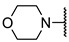	55	69
**f**	**9f**	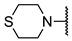	58	70

**Table 5:** Parameters of anticonvulsive activity of compounds **9b** and VPNa on the PTZ-induced seizures model. * *p* < 0.05.

Dose (mmole/kg)	Pre-Treatment Time (h)	Latency ± SEM (min)	Number Seizures ± SEM
Control	1	12.69 ± 2.06	1.22 ± 0.32
**9b** (0.50)		10.27 ± 2.38	1.16 ± 0.16
**9b** (1.00)		9.81 ± 1.67	3.33 ± 0.84 *
VPNa (0.50)		11.00 ± 4.36	0.50 ± 0.22
VPNa (1.00)		11.00 ± 3.06 *	0.50 ± 0.22
Control	4	4.24 ± 1.03	1.66 ± 0.40
**9b** (0.50)		11.22 ± 7.89	0.33 ± 0.21 *
**9b** (1.00)		9.79 ± 1.72	1.50 ± 0.34
VPNa (0.50)		14.00	0.16 ± 0.16 *
VPNa (1.00)		22.50 ± 3.50	0.33 ± 0.21 *

**Table 6:** Molecular descriptors values, experimental GABA-AT inhibition % (Y) and predicted ($Y_{pred}$) of GABA derivatives present in the QSAR model.

Molecule	T(O..S)	TI2	HOMAT	RCI	Y	$\;Y_{pred}\;$
**7a**	14	3.435	0	0	24.9	25.35
**7b**	0	3.483	0	0	24.9	18.62
**7c**	0	3.503	0	0	19.4	21.27
**7d**	0	3.532	0	0	22.9	21.8
**7e**	0	3.483	0	0	18.3	20.67
**7f**	16	3.483	0	0	26.5	29.65
**8a**	14	2.755	0	0	−3.5	5.54
**8b**	0	2.95	0	0	0.84	2.06
**8c**	0	3.106	0	0	5.96	7.11
**8d**	0	3.135	0	0	2.64	9.2
**8e**	0	2.95	0	0	6.2	0.06
**8f**	16	2.95	0	0	16.8	6.31
**9b**	0	2.897	5.781	1.404	73	-
**9c**	0	3.08	5.747	1.408	28	27.46
**9d**	0	3.105	5.781	1.404	8.6	9.34
**9e**	0	2.897	5.771	1.404	7	5
**9f**	16	2.897	5.767	1.404	14.2	16.05
**VPNa**	0	2.02	0	0	40	-

**Table 7:** Experimental GABA-AT inhibition % (Y), calculated ($Y_{cal}$) and predicted ($Y_{pred}$) for the GABA analogues in this study. The calculated and predicted residual values ($residual_{cal}$ and $residual_{pred}$) are also shown.

Molecule	Y	$\;Y_{cal}\;$	$\;Y_{pred}\;$	$\;Residual_{cal}\;$	$\;Residual_{pred}\;$
**7a**	24.9	-	25.35	-	0.45
**7b**	24.9	20.11	18.62	−4.79	−6.28
**7c**	19.4	20.8	21.27	1.4	1.87
**7d**	22.9	-	21.8	-	−1.1
**7e**	18.3	20.11	20.67	1.81	2.37
**7f**	26.5	27.99	29.65	1.49	3.15
**8a**	−3.5	1.9	5.54	5.4	9.04
**8b**	0.84	1.73	2.06	0.89	1.22
**8c**	5.96	-	7.11	-	1.15
**8d**	2.64	8.11	9.2	5.47	6.56
**8e**	6.2	1.73	0.06	−4.47	−6.14
**8f**	16.8	9.61	6.31	−7.19	−10.49
**9b**	73	-	-	-	-
**9c**	28	27.98	27.46	−0.02	−0.54
**9d**	8.6	8.94	9.34	0.34	0.74
**9e**	7	5.71	5	−1.29	−2
**9f**	14.2	15.17	16.05	0.97	1.85
**VPNa**	40	-	-	-	-

**Table 8:** Molecular descriptors obtained from DRAGON 05.

Molecular Descriptors	Type
*MW, AMW, Sv, Se, Sp, Ss, Mv, Me, Mp, Ms, nAT, nSK, nBT, nBO, nBM, SCBO, ARR, nCIC, nCIR, RBN, RBF, nAB, nH, nC, nO, nS, nCL, nX, nR06, nBnz*	Constitutional
*ZM1, ZM1V, ZM2, ZM2V, Qindex, SNar, HNar, GNar, Xt, Dz, Ram, Pol, LPRS, VDA, MSD, SMTI, SMTIV, GMTI, GMTIV, Xu, SPI, W, WA, Har, Har2, QW, TI1, TI2, STN, HyDp, RHyDp, w, ww, Rww, D/D, Wap, WhetZ, Whetm, Whetv, Whete, Whetp, J, JhetZ, Jhetm, Jhetv, Jhete, Jhetp, MAXDN, MAXDP, DELS, TIE, S0K, S1K, S2K, S3K, PHI, BLI, PW2, PW3, PW4, PW5, PJI2, CSI, ECC, AECC, DECC, MDDD, UNIP, CENT, VAR, BAC, Lop, ICR, D/Dr06, T(N..O), T(N..S), T(N..Cl), T(O..O), T(O..S), T(O..Cl)*	Topological
*W3D, J3D, H3D, AGDD, DDI, ADDD, G1, G2, RGyr, SPAN, SPAM, SPH, ASP, FDI, PJI3, L/Bw, SEig, HOMA, RCI, AROM, HOMT, DISPm, QXXm, QYYm, QZZm, DISPv, QXXv, QYYv, QZZv, DISPe, QXXe, QYYe, QZZe, DISPp, QXXp, QYYp, QZZp, G(N..O), G(N..S), G(N..Cl), G(O..O), G(O..S), G(O..Cl)*	Geometrical
*nCp, nCs, nCt, nCrs, nCrt, nCar, nCbH, nCb-, nRNH2, nRNR2, nROR, nRSR, nArX, nHDon, nHAcc, C-001, C-002, C-003, C-006, C-024, C-025, C-026, H-046, H-047, H-050, H-052, O-059, N-066, N-068, Cl-089, S-107*	functional group counts
*Ui, Hy, AMR, TPSA (NO), TPSA (tot), MLogP, MLogP^2^, ALogP, ALogP^2^*	Molecular properties
*qpmax, qnmax, Qpos, Qneg, Qtot, Qmean, Q2, RPCG, RNCG, SPP, TE1, TE2, PCWTe, LDI*	Atomic charge

**Table 9:** GABA aminotransferase crystal structures available in PDB server.

PDB_ID	Resolution (Å)	Organism	% Identity Respect to Human	% Identity Respect to *P. fluorencens*
4zsw	1.70	*Wild boar*	95.46	27.52
4y0h	1.63	*Wild boar*	95.46	27.52
4y0i	1.66	*Wild boar*	95.46	27.52
1ohv	2.30	*Wild boar*	95.46	27.52
1ohw	2.30	*Wild boar*	95.46	27.52
1sf2	2.40	*E. coli*	26.35	73.82
4ffc	1.80	*M. abscessus*	27.99	42.79

## Supplementary Materials

The supplementary materials are available online. Figures S1–S40: describe NMR data for all compounds, Figures S41–S52: computational data, Tables S1–S3: describe computational data.

## References

[1] Kandel E.R., Schartz J.H., Jessell T.M. (2000). Principles of Neural Science.

[2] Niciu M.J., Kelmendi B., Sanacora G. (2012). Overview of glutamatergiC-Neurotransmission in the nrevous system. Pharm. Biochem. Behav..

[3] Amadasi A., Bertoldi M., Contestabile R., Bettati S., Cellini B., di Salvo M.L., Borri-Voltattorni C., Bossa F., Mozzarelli A. (2007). Piridoxal 5′-phosphate enzymes as targets for therapeutic agents. Curr. Med. Chem..

[4] Baxter C.F., Roberts E.J. (1958). The γ-Aminobutyric Acid-α-Ketoglutaric Acid Transaminase of Beef Brain. J. Biol. Chem..

[5] Bakay R.A., Harris A.B. (1981). Neurotransmitter, receptor and biochemical changes in monkey cortical epileptic foci. Brain Res..

[6] Aoyagi T., Wada T., Nagai M., Kojima F., Harada S., Takeuchi T., Takahashi H., Hirokawa K., Tsumita T. (1990). Increased γ-aminobutyrate aminotransferase activity in brain of patients with Alzheimer’s disease. Chem. Pharm. Bull..

[7] Nishino N., Fujiwara H., Noguchi-Kuno S.A., Tanaka C. (1988). GABA_A_ receptor but not muscarinic receptor density was decreased in the brain of patients with Parkinson’s disease. Jpn. J. Pharmacol..

[8] Ebato H., Seyfried T.N., Yu R.K. (1983). Biochemical study of heterosis for brain myelin content in mice. J. Neurochem..

[9] Hersh D.S., Wadajkar A.S., Roberts N.B., Woodworth G.F., Kim A.J. (2016). Evolving drug delivery strategies to overcome the blood brain barrier. Curr. Pharm. Des..

[10] Li Z., Taylor C.P., Weber M., Piechan J., Prior F., Bian F., Cui M., Hoffman D., Donevan S. (2011). Pregabalin is a potent and selective ligand for α2δ-1 andα2δ-2 calcium channels subunits. Eur. J. Pharm..

[11] Silverman R.B. (2008). From basic science to blockbuster drug: The discovery of Lyrica. Angew. Chem. Int. Ed..

[12] Reinares M., Rosa A.R., Franco C., Goikolea J.M., Fountoulakis K., Siamouli M., Gonda X., Frangou S., Vieta E. (2013). A systematic review on the role of anticonvulsants in the treatment of acute bipolar depression. Int. J. Neuropsychopharmacol..

[13] Wensel T.M., Powe K.W., Cates M.E. (2012). Pregabalin for the treatment of generalized anxiety disorder. Ann. Pharmacother..

[14] Bellioti T.R., Capiris T., Ekhato V., Kinsora J.J., Field M.J., Heffner T.G., Meltzer L.T., Schwarz J.B., Taylor C.P., Thorpe A.J. (2005). Structure–activity relationships of Pregabalin and analogues that target the α2-δ protein. J. Med. Chem..

[15] Bowery N.E. (1982). Baclofen: 10 years on. Trends Pharm. Sci..

[16] Olpe H.R., Demie’ville H., Baltzer V., Bencze E.L., Koella W.P., Wolf P., Haas H.L. (1978). The biological activity of d-Baclofen (Lipresal^®^). Eur. J. Pharmacol..

[17] Schelkun R.M., Yuen P.-W., Wustrow D.J., Kinsora J., Su T.-Z., Vartanian M.G. (2006). Heteroaromatic side-chain analogs of Pregabalin. Bioorg. Med. Chem. Lett..

[18] Brow K.M., Roy K.K., Hockerman G.H., Doerksen R.J., Colby D.A. (2015). Activation of the γ-Aminobutyric Acid Type B (GABA_B_) Receptor by Agonists and Positive Allosteric Modulators. J. Med. Chem..

[19] Attia M., Herdeis C., Osborne H.B. (2013). GABAB-antagonistic activity of certain Baclofen homologues. Molecules.

[20] Xu F., Peng G., Phan T., Dilip U., Chen J.L., Chernov-Rogan T., Zhang X., Grindstaff K., Amamalai T., Koller K. (2011). Discovery of a novel potent GABA_B_ receptor agonist. Bioorg. Med. Chem. Lett..

[21] Abdel-Hafez A.A., Abdel-Wahab B.A. (2008). 5-(4-chlorophenyl)-5,6-duhydro-1,3-oxazepin-7(4h)-one derivatives as lipophilic cyclic analogues of Baclofen: Design, synthesis and neuropharmacological evaluation. Bioorg. Med. Chem..

[22] Constantino G., Macchiarulo A., Entrena Guadix A., Pelliciari R. (2001). QSAR and molecular modeling studies of Baclofen analogues as GABA_B_ agonists. Insights into the role of the aromatic moiety in GABA_B_ binding in activation. J. Med. Chem..

[23] Steffan T., Renukappa-Gutke T., Höfner G., Wanner K.T. (2015). Design synthesis and SAR studies of GABA uptake inhibitors derived from 2-substituted pyrrolidine-2-yl acetic acids. Bioorg. Med. Chem..

[24] Nielsen L., Brehm L., Krogsgaard-Larsen P. (1990). GABA agonists and uptake inhibitors. Synthesis, absolute stereochemistry, and enantioselectivity of (*R*)-(−)- and (*S*)-(+)-homo-.beta.-proline. J. Med. Chem..

[25] Silverman R.B. (2018). Design and Mechanism of GABA Aminotransferase Inactivators. Treatments for Epilepsies and Addictions. Chem. Rev..

[26] Sulaiman-Saba A.J., Suliman F.E.O., Barghouthi S. (2003). Kinetic Studies on the Inhibition of GABA-T by γ-Vinyl GABA and Taurine. J. Enzym. Inhib. Med. Chem..

[27] Liu W., Peterson P.E., Carter R.J., Zhou X., Langston J.A., Fisher A.J., Toney M.D. (2004). Crystal Structures of Unbound and Aminooxyacetate-Bound *Escherichia coli* γ-Aminobutyrate Aminotransferase. Biochemistry.

[28] Burke J.R., Silverman R.B. (1991). Mechanism of inactivation of γ-aminobutyiric acid aminotransferase by 4-amino-5-hexynoic acid (γ-ethynylGABA). J. Am. Chem. Soc..

[29] Hawker D.D., Silverman R.B. (2012). Synthesis and evaluation of novel heteroaromatic substrates of GABA aminotransferase. Bioorg. Med. Chem..

[30] Clift M.D., Silverman R.B. (2008). Synthesis and evaluation of novel aromatic substrates and competitive inhibitors of GABA aminotransferase. Bioorg. Med. Chem..

[31] Yuan H., Silverman R.B. (2006). New substrates and inhibitors of γ-aminobutyric acid aminotransferase containing bioisosteres of the carboxylic acid group: Design, synthesis, and biological activity. Bioorg. Med. Chem..

[32] Le H.V., Hawker D.D., Wu R., Doud E., Widom J., Sanishvili R., Liu D., Lelleher N.L., Silverman R.B. (2015). Design and mechanism of tetrahydrothiophene-based-γ-aminobutyiric acid aminotransferase inactivators. J. Am. Chem. Soc..

[33] Lee H., Le H.V., Wu R., Doud E., Sanishvili R., Kellie J.F., Compton P.D., Pachaiyappan B., Liu D., Kelleher N.L. (2015). Mechanism of inactivation of GABA aminotransferase by (*E*)- and (*Z*)-(1*S*,3*S*)-3-amino-4-fluoromethylenyl-1-cyclopentanoic acid. ACS Chem. Biol..

[34] Juncosa J.L., Takaya K., Le H.V., Moschitto M.J., Weerawarna P.M., Mascarenhas R., Liu D., Dewey S.L., Silverman R.B. (2018). Design and Mechanism of (*S*)-3-Amino-4- (difluoromethylenyl)cyclopent-1-ene-1-carboxylic Acid, a Highly Potent γ-Aminobutyric Acid Aminotransferase Inactivator for the Treatment of Addiction. J. Am. Chem. Soc..

[35] Pinto A., Tamborini L., Pennacchietti E., Caluccia A., Silvestri A., Cullia G., De Micheli C., Conti A., De Biase D. (2016). Bicyclic γ-amino acids as inhibitors ot γ-aminobutyrate aminotransferase. J. Enzym. Inhib. Med. Chem..

[36] Rekatas G.V., Tani E., Demopoulos V.J., Kourounakis P.N. (1996). Synthesis of GABA-valproic acid derivatives and evaluation of their anticonvulsant and antioxidant activity. Arch. Pharm. Med. Chem..

[37] Sahu M., Siddiqui N., Sharma V., Wakode S. (2018). 5,6-Dihydropyrimidine-1(2H)-carbothioamides: Synthesis, in vitro GABA-AT screening, anticonvulsant activity and molecular modelling study. Bioorg. Chem..

[38] Bansal S.K., Sinha B.N., Khosa R.L. (2011). QSAR and docking based computational chemistry approach to novel GABA-AT inhibitors: *k*NN-MFZ-based 3dQSAR model for phenyl-substituted analogs of β-phenylethylidene hydrazine. Med. Chem. Res..

[39] Bansal S.K., Sinha B.N., Khosa R.L., Olson A.J. (2011). Novel GABA-AT inhibitors: QSAR and docking based virtual screening of β-phenyl substituted β-phenyl ethylidene hydrazine analogues. Med. Chem. Res..

[40] Davood A., Iman M. (2011). Docking and QSAR studies of β-phenylethylidenehydrazine derivatives as a Gamma-aminobutyric acid aminotransferase inhibitor. Med. Chem. Res..

[41] Abdulfatai U., Uzairu A., Uba S. (2018). Molecular docking and QSAR analysis of a few Gamma amino butyric acid amino transferase inhibitors. EJBAS.

[42] Abdulfatai U., Uzairu A., Uba S. (2017). Quantitative structure-activity relationship and molecular docking studies of a series of quinazolinonyl analogues as inhibitors of gamma amino butyric acid aminotransferase. J. Adv. Res..

[43] Singh Y., Jain J., Chowdhury P., Nainwal L. (2012). Study of halogen substitution docking and 3d QSAR properties of aryl substituted thiosemicarbazones as anticonvulsant. IJTA.

[44] Tovar-Gudiño E., Guevara-Salazar J.A., Bahena-Herrera J.R., Trujillo-Ferrara J.G., Martínez-Campos Z., Razo-Hernández R.S., Santiago A., Pastor N., Fernández-Zertuche M. (2018). Novel-Substituted Heterocyclic GABA Analogues. Enzymatic Activity against the GABA-AT Enzyme from *Pseudomonas fluorescens* and in Silico Molecular Modeling. Molecules.

[45] Kovalev G.V., Tyurenkov I.N., Perekalin V.V., Zobacheva M.M., Grineva V.S., Kiseleva I.N., Morozov I.S., Sobolev S.G., Papayan G.L., Vasilyeva O.S. (1979). Study of the relationship between the chemical structure and vasoactive properties of GABA derivatives. Trudy Volgogradskogo Gosudarstvennogo Meditsinskogo Instituta.

[46] Lipinski C.A., Lombardo F., Dominy B.W., Feeney P.J. (1997). Experimental and computational approaches to estimate solubility and permeability in drug discovery and development settings. Adv. Drug Deliv. Rev..

[47] Zanaletti R., Bettinetti L., Castaldo C., Ceccarelli I., Cocconcelli G., Comery T.A., Dunlop J., Genesio E., Ghiron C., Haydar S.N. (2012). N-[5-(5-Fluoropyridin3-yl)-1H-pyrazol-3-yl]-4-piperidin-1-ylbutyramide (SEN78702, WYE308775): A medicinal chemistry effort toward an a7 nicotinic acetylcholine receptor agonist Preclinical candidate. J. Med. Chem..

[48] Dega-Szafran Z., Gaszczyk I., Maciejewska D., Szafran M., Tykarska E., Wawer I. (2001). 13C CP MAS NMR, FTIR, X-ray diffraction and PM3 studies of some N-(ω-carboxyalkyl)morpholine hydrohalides. J. Mol. Struct..

[49] Harris L.S., Pars H.G., Razdan R.K., Sheehan J.C. (1976). Heterocyclic esters of Benzopyrans. U.S. Patent.

[50] Tunnicliff G., Crites G. (1998). Chemical Inactivation of Bacterial GABA Aminotransferase. Biochem. Mol. Biol. Int..

[51] Yogeeswari P., Sriram D., Thirumurugan R., Jit L.R.J.S., Ravagendran J.V., Kavya R., Rakhra K., Saraswat V. (2006). Synthesis of N^4^-(2,4-dimethylphenyl) semicarbazones as 4-aminobutyrate aminotransferase inhibitors. Acta Pharm..

[52] Marquez Darche Cantú M.J. (1991). Probability and Statistics for Biological Chemistry Sciences.

[53] Yang J., Sun C., Fu D., Yu T. (2017). Test for L-glutamate inhibition of growth of *Alternaria alternate* by inducing resistance in tomato fruit. Food Chem..

[54] Giacomini M., Bisio A., Giacomelli E., Pivetti S., Bertolini S., Fraternale D., Ricci D., Romussi G., De Tommasi N. (2011). Data collection and advanced statistical analysis of aerial exudates of Salvia spp.. Rev. Bras. Farmcongn..

[55] Bruchey A.K., Gonzalez-Lima F. (2008). Behavioral, physiological and biochemical hormetic responses to the autoxidizable dye methylene blue. Am. J. Pharmacol. Toxicol..

[56] Fukushima S., Kinoshita A., Puatanachokchai R., Kushida M., Wanibuchi H., Morimura K. (2005). Hormesis and dose-response-mediated mechanisms in carcinogenesis: Evidence for a threshold in carcinogenicity of non-genotoxic carcinogens. Carcinogenesis.

[57] Calabrese E.J., Calabrese V., Giordiano J. (2017). The role of hormesis in the functional performance and protection of neural systems. Brain Circ..

[58] Calabrese E.J. (2008). Modulation of the epileptic seizure threshold: Implications of biphasic dose responses. Crit. Rev. Toxicol..

[59] Calabrese E.J. (2008). Hormesis and medicine. Br. J. Clin. Pharmacol..

[60] Jug K. (1984). Bond order as a Tool for Molecular Structure and Reactivity. Croat. Chem. Acta.

[61] Krygowski T.M., Cyrański M., Ciesielski A., Świrska B., Leszczyński P. (1996). Separation of the Energetic and Geometric Contributions to Aromaticity. 2. Analysis of the Aromatic Character of Benzene Rings in Their Various Topological Environments in the Benzenoid Hydrocarbons. Crystal and Molecular Structure of Coronene. J. Chem. Inf. Comput. Sci..

[62] Sakar A., Middya T.R., Jana A.D. (2012). A QSAR study of radical scavenging antioxidant activity of a series of flavonoids using DFT based quantum chemical descriptors—The importance of group frontier electron density. J. Mol. Model..

[63] Huang R.Q., Dillon G.H. (2001). Pentylenetetrazole-induced inhibition of recombinant γ-aminobutyric acid type A (GABA_A_) receptors: Mechanism and site action. J. Pharmacol. Exp. Ther..

[64] Luszczki J.J., Wojcik-Cwokla J., Andres M.M., Czuczwar S.J. (2005). Phamacological and behavioural characteristics of interactions between vigabatrin and conventional antiepileptic drugs in pentylenetetrazole-induced seizures in mice: An isobolographic analysis. Neuropsychopharmacology.

[65] Luszczki J.J., Zuchora M., Sawicka K.M., Czuczwar S.J. (2006). Acute exposure to caffeine decreases the anticonvulsant action of ethosuximide, but not that of clonazepam, phenobarbital and valproate against pentetrazole-induced seizures in mice. Pharmacol. Rep..

[66] Sugaya E., Ishige A., Sekiguchi K. (1986). Pentylenetetrazole-induced convulsion and effect of anticonvulsants in mutant inbred strain E1 mice. Epilepsia.

[67] Clark M., Cramer R.D., Van Opdenbosch N.V. (1989). Validation of the general purpose tripos 5.2 force field. J. Comput. Chem..

[68] Stewart J.J.P. (1989). Optimization of parameters for semiempirical methods I. Method. J. Comput. Chem..

[69] Stephens P.J., Devlin F.J., Chabalowski C.F., Frisch M.J. (1994). Ab Initio Calculation of Vibrational Absorption and Circular Dichroism Spectra Using Density Functional Force Fields. J. Phys. Chem..

[70] Petersson G.A., Tensfeldt T.G., Montgomery Jr J.A. (1991). A complete basis set model chemistry. III. The complete basis set-quadratic configuration interaction family of methods. J. Chem. Phys..

[71] Wavefunction, Inc. Spartan’08. http://www.wavefun.com.

[72] Talete SRI (2006). DRAGON for Windows (Software for Molecular Descriptor Calculations).

[73] Todeschini R., Ballabio D.C., Mauri V. (2004). MobyDigs-Version 1.0.

[74] Todeschini R., Consonni V., Mauri A., Pavan M. (2004). Detecting “bad” regression models: Multicriteria fitness functions in regression analysis. Anal. Chim. Acta.

[75] Razo-Hernández R.S., Pineda-Urbina K., Velazco-Medel M.A., Villanueva-García M., Sumaya-Martínez M.T., Martínez-Martínez F.J., Gómez-Sandoval Z. (2014). QSAR study of the DPPH·radical scavenging activity of coumarin derivatives and xanthine oxidase inhibition by molecular docking. Cent. Eur. J. Chem..

[76] Pérez D.J., Sarabia O., Villanueva-García M., Pineda-Urbina K., Ramos-Organillo A., González-González J., Gómez-Sandoval Z., Razo-Hernández R.S. (2017). In silico receptor-based drug design of X, Y-benzenesulfonamide derivatives as selective COX-2 inhibitors. C. R. Chim..

[77] Šali A., Blundell T.L. (1993). Comparative protein modelling by satisfaction of spatial restraints. J. Mol. Biol..

[78] Huang J., MacKerell A.D. (2013). CHARMM36 all-atom additive protein force field: Validation based on comparison to NMR data. J. Comput. Chem..

[79] Yang J.M., Chen C.-C. (2004). GEMDOCK: A generic evolutionary method for molecular docking. Proteins Struct. Funct. Bioinform..

[80] Thomsen R., Christensen M.H. (2006). A New Technique for High-Accuracy Molecular Docking. J. Med. Chem..

