# Supplementary material for: QSAR and Molecular Docking Studies of the Inhibitory Activity of Novel Heterocyclic GABA Analogues over GABA-AT
Source: Molecules. 2018 Nov 15;23(11):2984. doi: 10.3390/molecules23112984 (PMC6278377; doi:10.3390/molecules23112984)
Supplement: Supplementary file 1 [file molecules-23-02984-s001.pdf]

Supplementary Material

QSAR and Molecular Docking Studies of the Inhibitory Activity of Novel Heterocyclic GABA Analogues over GABA-AT

Josué Rodríguez-Lozada[1], Erika Tovar-Gudiño[1], Juan Alberto Guevara-Salazar[2], Rodrigo Said Razo-Hernández[3], Ángel Santiago[3], Nina Pastor[3], Mario Fernández-Zertuche[1*]

Table of Contents

NMR Data for analogues **7**, **8** and **9** 08
Computational Details 24

NMR Data for analogues **7**, **8** and **9**

Figure 1S. ^{1}H NMR (200 MHz, CD_3OD) of 4-(thiazolidin-3-yl)butanoic acid (**7a**) 06

Figure 2S. ^{13}C NMR (50 MHz, CD_3OD) of 4-(thiazolidin-3-yl)butanoic acid (**7a**) 06

Figure 3S. ^{1}H NMR (400 MHz, CD_3OD) of 4-(piperidin-1-yl)butanoic acid (**7b**). 07

Figure 4S. ^{13}C NMR (400 MHz, CD_3OD) of 4-(piperidin-1-yl)butanoic acid (**7b**). 07

Figure 5S. ^{1}H NMR (400 MHz, D_2O) of 4-(3-methylpiperidin-1-yl)butanoic acid (**7c**) 07
.
Figure 6S. ^{13}C NMR (100 MHz, D_2O) of sodium 4-(3-methylpiperidin-1-yl)butanoic acid (**7c**). 08

Figure 7S. ^{1}H NMR (400 MHz, CD_3OD) of 4-(4-methylpiperidin-1-yl)butanoic acid (**7d**). 08

Figure 8S. ^{13}C NMR (100 MHz, CD_3OD) of 4-(4-methylpiperidin-1-yl)butanoic acid (**7d**). 08

Figure 9S. ^{1}H NMR (400 MHz, CD_3OD) of 4-morpholinobutanoic acid (**7e**). 09

Figure 10S. ^{13}C NMR (100 MHz, CD_3OD) of 09

4-morpholinobutanoic acid (**7e**).

Figure 11S. ^{1}H NMR (400 MHz, CD_3OD) of 4-thiomorpholinobutanoic acid (**7f**). 09

Figure 12S. ^{13}C NMR (100 MHz, CD_3OD) of 4-thiomorpholinobutanoic acid (**7f**). 10

Figure 13S. ^{1}H NMR (400 MHz, CD_3OD) of 5-methyl-3-(thiazolidin-3-ylmethyl)hexanoic acid (**8a)**. 10

Figure 14S. ^{13}C NMR (100 MHz, CD_3OD) of 5-methyl-3-(thiazolidin-3-ylmethyl)hexanoic acid (**8a**). 10

Figure 15S. 2D NMR (HETCOR 400 MHz, CD_3OD) of 5-methyl-3-(thiazolidin-3-ylmethyl)hexanoic acid (**8a**). 11

Figure 16S. ^{1}H NMR (400 MHz, CD_3OD) of 5-methyl-3-(piperidin-1-ylmethyl)hexanoic acid (**8b**). 11

Figure 17S. ^{13}C NMR (100 MHz, CD_3OD) of 5-methyl-3-(piperidin-1-ylmethyl)hexanoic acid (**8b**). 12

Figure 18S. 2D NMR (HETCOR 400 MHz, CD_3OD) of 5-methyl-3-(piperidina-1-ylmethyl) hexanoic acid (**8b**). 12

Figure 19S. ^{1}H NMR (400 MHz, CD_3OD) of 5-methyl-3-((3-methylpiperidin-1 yl)methyl)hexanoic acid (**8c**). 13

Figure 20S. ^{13}C NMR (100 MHz, CD_3OD) of 5-methyl-3-((3-methylpiperidin-1-yl)methyl)hexanoic acid (**8c**). 13

Figure 21S. 2D NMR (HETCOR 400 MHz, CD_3OD) of 5-methyl-3-((3-methylpiperidin-1-yl)methyl)hexanoic acid (**8c**). 14

Figure 22S. ^{1}H NMR (400 MHz, CD_3OD) of 5-methyl-3-((4-methylpiperidin-1-yl)methyl)hexanoic acid (**8d**). 14

Figure 23S. ^{13}C NMR (100 MHz, CD_3OD) of 5-methyl-3-((4-methylpiperidin-1-yl)methyl)hexanoic acid (**8d**). 15

Figure 24S. 2D NMR (HETCOR 400 MHz, CD_3OD) of 5-methyl-3-((4-methylpiperidin-1-yl)methyl)hexanoic acid (**8d**). 16

Figure 25S. ^{1}H NMR (400 MHz, CD_3OD) of 5-methyl-3-(morpholinomethyl)hexanoic acid (**8e**). 16

Figure 26S. ^{13}C NMR (100 MHz, CD_3OD) of 5-methyl-3-(morpholinomethyl)hexanoic acid (**8e)**. 17

Figure 27S. ^{1}H NMR (400 MHz, CD_3OD) of 5-methyl-3-(thiomorpholinomethyl)hexanoic acid (**8e**). 17

Figure 28S. ^{13}C NMR (100 MHz, CD_3OD) of 5-methyl-3-(thiomorpholinomethyl)hexanoic acid (**8f**). 18

Figure 29S. ^{1}H NMR (400 MHz, CD_3OD) of 3-(4-chlorophenyl)-4-(piperidin-1-yl)butanoic acid (**9b**). 18

Figure 30S. ^{13}C NMR (100 MHz, CD_3OD) of 3-(4-chlorophenyl)-4-(piperidin-1-yl)butanoic acid (**9b)**. 18

Figure 31S. ^{1}H NMR (400 MHz, CD_3OD) of 3-(4-chlorophenyl)-4-(3-methylpiperidin-1-yl)butanoic acid (**9c**). 19

Figure 32S. ^{13}C NMR (100 MHz, CD_3OD) of 3-(4-chlorophenyl)-4-(3-methylpiperidin-1-yl)butanoic acid (**9c**). 19

Figure 33S. ^{1}H NMR (400 MHz, CD_3OD) of 3-(4-chlorophenyl)-4-(4-methylpiperidin-1-yl) butanoic acid (**9d**). 19

Figure 34S. ^{13}C NMR (100 MHz, CD_3OD) of 3-(4-chlorophenyl)-4-(4-methylpiperidin-1-yl) butanoic acid (**9d**). 20

Figure 35S. 2D NMR (HETCOR 400 MHz, CD_3OD) of 3-(4-chlorophenyl)-4-(4-methylpiperidin-1-yl)butanoic acid (**9d**). 20

Figure 36S. ^{1}H NMR (400 MHz, CD_3OD) of 3-(4-chlorophenyl)-4-morpholinobutanoic acid (**9e**). 21

Figure 37S. ^{13}C NMR (100 MHz, CD_3OD) of 3-(4-chlorophenyl)-4-morpholinobutanoic acid (**9e)**. 21

Figure 38S. ^{1}H NMR (400 MHz, CD_3OD) of 3-(4-chlorophenyl)-4-thiomorpholinobutanoic acid (**9f**). 22

Figure 39S. ^{13}C NMR (100 MHz, CD_3OD) of 3-(4-chlorophenyl)-4-thiomorpholinobutanoic acid (**9f**). 22

Figure 40S. 2D NMR (HETCOR 400 MHz, CD_3OD) of 3-(4-chlorophenyl)-4-thiomorpholinobutanoic acid (**9f**). 23

Figure 41S. Alignment of *pseudomonas fluorencens* (**PF**), *human* (HS), *E. coli* (EC) and *wild boar* (JB). Red and blue color letters corresponds to the residues of the chain A and chain B respectively, that interact with vigabatrin in the 1ohv crystal structure 24

Figure 42S. Validation of the molecular docking calculation for the pseudomonas model. Ligand in the PDB:ID 3r4t crystal structure was reproduced with a RMSD of 1.7 Å. Ligand experimental (opaque color) and calculated conformation (shiny color) are displayed as sticks representation respectively. Residues within 4.0 Å of both ligands are shown as thin sticks 25

Figure 43S. Validation of the molecular docking calculation for the *human* model. Ligand in the PDB:ID 1ohw crystal structure was reproduced with a RMSD of 1.3 Å. Ligand experimental (opaque color) and calculated conformation (shiny color) are displayed as sticks representation respectively. Residues within 4.0 Å of both ligands are shown as thin sticks. 25

Figure 44S. Validation of the molecular docking calculation for the *human* model. Ligand in the PDB:ID 1ohy crystal structure was reproduced with a RMSD of 1.8 Å. Ligand experimental (opaque color) and calculated conformation (shiny color) are displayed as sticks representation respectively. Residues within 4.0 Å of both ligands are shown as thin sticks. 26

Figure 45S. Optimized structures of all GABA analogues, VPNa and VGB. 27

Figure 46S. Interactions between GABA analogues **7** and *Pseudomonas fluorescens* GABA-AT. a) **7a**, b) **7b**, c) **7c**, d) **7d**, e) **7e** and f) **7f**. PLP prosthetic group is showed as Van der Waals spheres and each protein chain is colored in green and cyan. Residues at 4 Å of each analogue are indicated. Hydrogen bonds are shown as orange dashed lines. 30

Figure 47S. Interactions between GABA analogues **8** and *Pseudomonas fluorescens* GABA-AT. a) (*S*)-**8a**, b) (*R*)-8**a**, c) (*S*)-**8b**, d) (*R*)-**8b**, e) (*S*)-**8c** and f) (*R*)-**8c**, g) (*S*)-**8d**, h) (*R*)-**8d**, i) (*S*)-**8e**, j) (*R*)-**8e**, k) (*S*)-**8f**, l) (*R*)-**8f**. PLP prosthetic group is showed as Van der Waals spheres and each protein chain is colored in green and cyan. Residues at 4 Å of each analogue are indicated. Hydrogen bonds are shown as orange dashed lines. 31

Figure 48S. Interactions between GABA analogues **9** and *Pseudomonas fluorescens* GABA-AT. a) (*S*)-**9c**, b) (*R*)-**9c**, c) (*S*)9**2d** and d) (*R*)-**9d**, e) (*S*)-**9e**, f) (*R*)-**9e**, g) (*S*)-**9f**, h) (*R*)-**9f**. PLP prosthetic group is showed as Van der Waals spheres and each protein chain is colored in green and cyan. Residues at 4 Å of each analogue are indicated. Hydrogen bonds are shown as orange dashed lines. 33

Table 1S. Energy interactions values obtained from the docking calculations of all GABA derivatives and *pseudomonas* GABA-AT model. All the values are in kcal/mol. 34

Figure 49S. Interactions between GABA analogues **7** and *Human* GABA-AT. a) **7a**, b) **7b**, c) **7c**, d) **7d**, e) **7e** and f) **7f**. PLP prosthetic group is showed as Van der Waals spheres and each protein chain is colored in yellow and red. Residues at 4 Å of each analogue are indicated. Hydrogen bonds are shown as orange dashed lines. 35

Figure 50S. Interactions between GABA analogues **8** and *Human* GABA-AT. a) (*S*)-**8a**, b) (*R*)-**8a**, c) (*S*)-**8b**, d) (*R*)-**8b**, e) (*S*)-**8c** and f) (*R*)-**8c**, g) (*S*)-**8d**, h) (*R*)-**8d**, i) (*S*)-**8e**, j) (*R*)-**8e**, k) (*S*)-**8f**, l) (*R*)-**8f**. PLP prosthetic group is showed as Van der Waals spheres and each protein chain is colored in yellow and red. Residues at 4 Å of each analogue are indicated. Hydrogen bonds are shown as orange dashed lines. 37

Figure 51S. Interactions between GABA analogues **9** and *Human* GABA-AT. a) (*S*)-**9b**, b) (*R*)-**9b**, c) (*S*)-**9c** and d) (*R*)-**9c**, e) (*S*)-**9d**, f) (*R*)-**9d**, g) (*S*)-**9e**, h) (*R*)-**9e**, i) (*S*)-**9f**, j) (*R*)-**9f**. PLP prosthetic group is showed as Van der Waals spheres and each protein chain is colored in yellow and red. Residues at 4 Å of each analogue are indicated. Hydrogen bonds are shown as orange dashed lines. 39

Figure 52S. Backbone structural alignment of GABA-AT structures. a) GABA-AT human model in cyan color. **1ohv**, **1ohw** and **1ohy** Sus scrofa crystal structures in red (RMSD=0.35), gray (RMSD=0.36) and orange (RMSD=0.40) color respectively. b) GABA-AT Pseudomonas fluorescens model in shiny red color, **1sf2** E. coli estructure in shiny yellow color (RMSD= 0.52). Fe_2S_2 (yellow/pink color) and PLP from human model in VDW representation. 40

Table 2S. Energy interactions values obtained from the docking calculations of all GABA derivatives and *human* GABA-AT model. All the values are in kcal/mol. 41

Table 3S. Values of the experimental (Y_{Exp}), calculated (Y_{Cal}) and predicted (Y_{Pred}) percent of inhibition of the GABA derivatives. Compounds that were considered form the test validation are marked with a script symbol. 42

NMR Spectra of compounds.

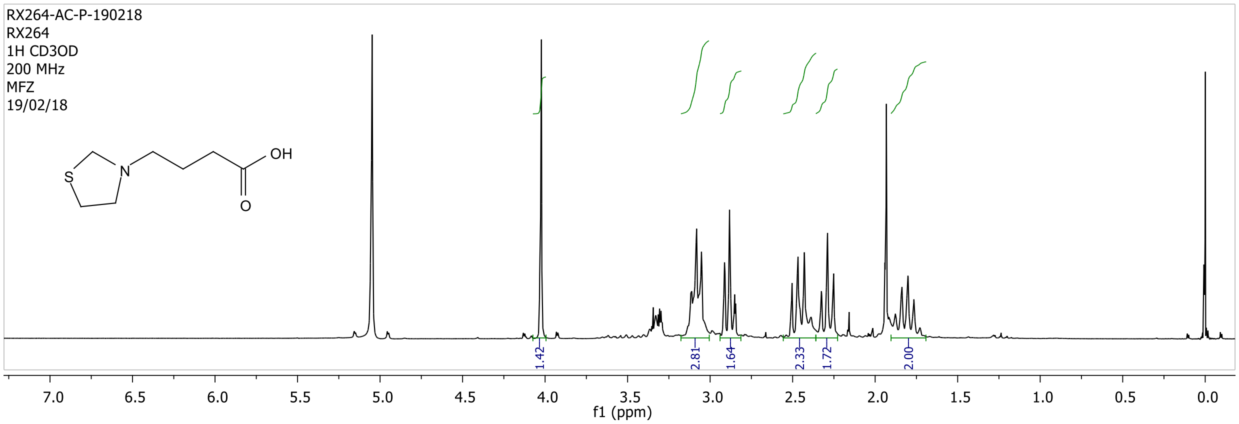

Figure 1S. ^{1}H NMR (200 MHz, CD_3OD) of 4-(thiazolidin-3-yl)butanoic acid (**7a**).

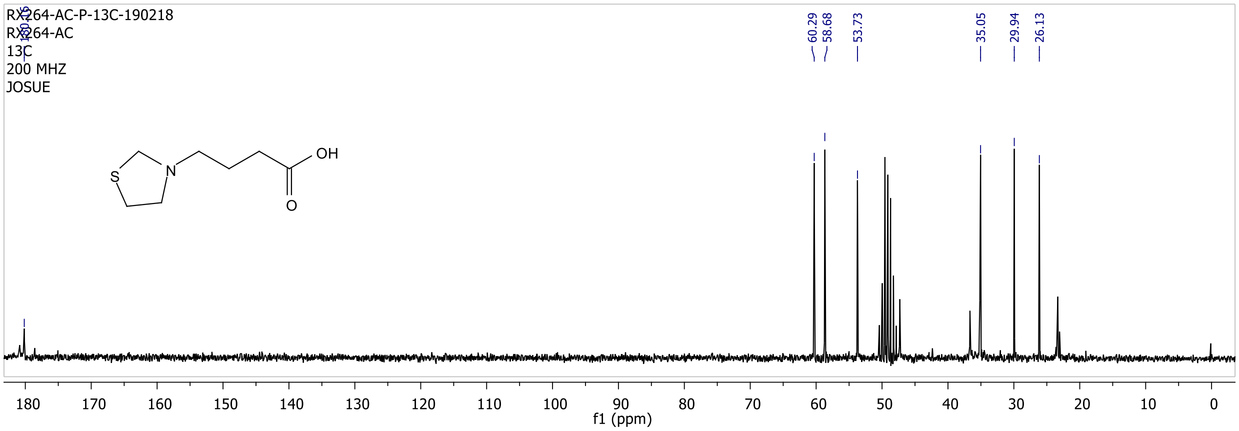

Figure 2S. ^{13}C NMR (50 MHz, CD_3OD) of 4-(thiazolidin-3-yl)butanoic acid (**7a**)

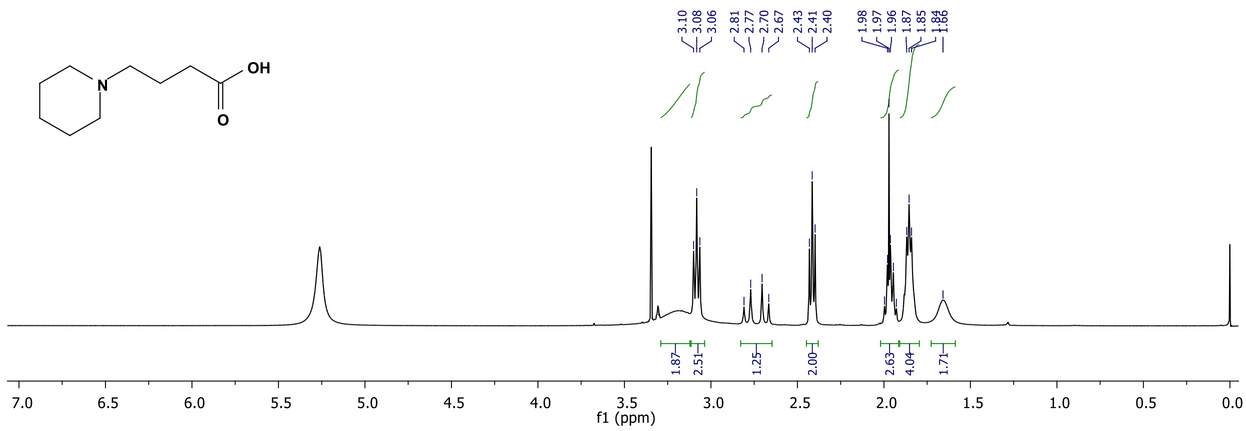

Figure 3S. ^{1}H NMR (400 MHz, CD_3OD) of 4-(piperidin-1-yl)butanoic acid (**7b**).

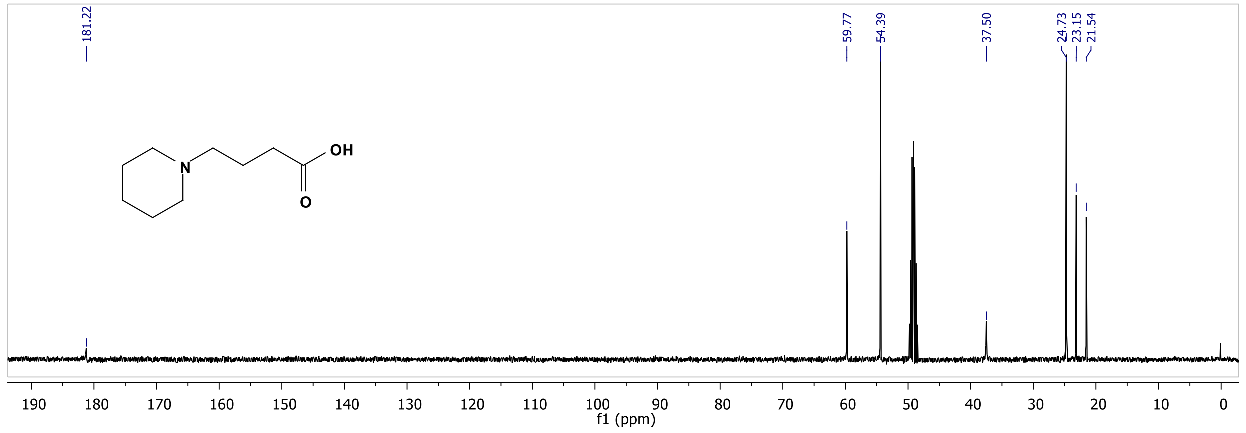

Figure 4S. ^{13}C NMR (400 MHz, CD_3OD) of 4-(piperidin-1-yl)butanoic acid (**7b**).

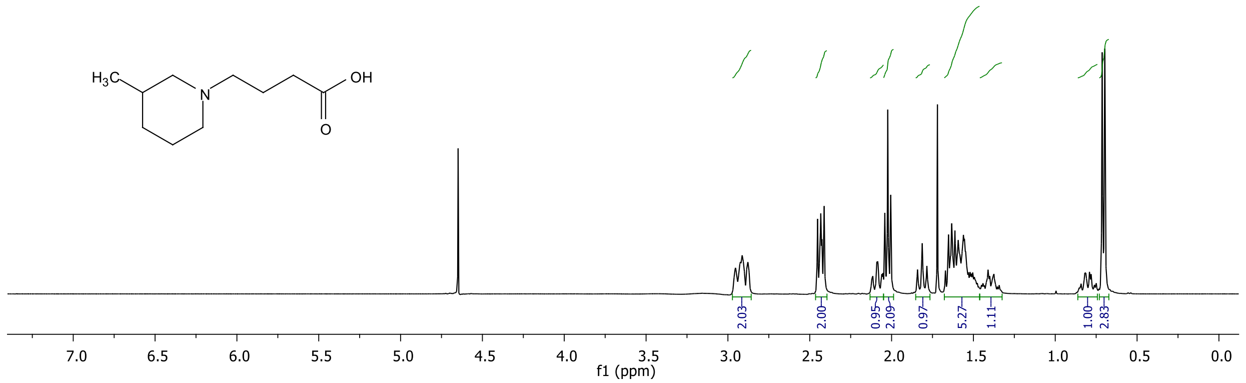

Figure 5S. ^{1}H NMR (400 MHz, D_2O) of 4-(3-methylpiperidin-1-yl)butanoic acid (**7c**).

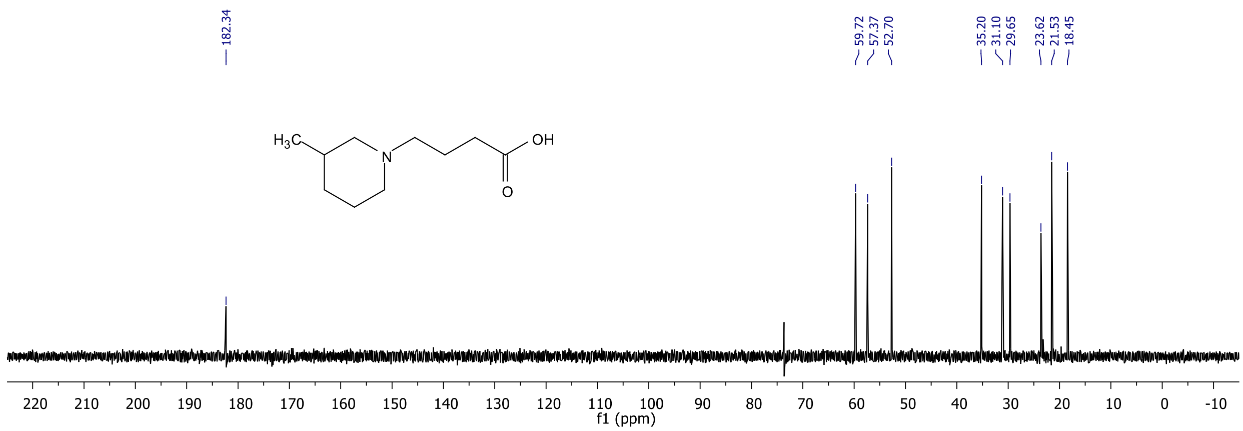

Figure 6S. ^{13}C NMR (100 MHz, D_2O) of sodium 4-(3-methylpiperidin-1-yl)butanoic acid (**7c**).

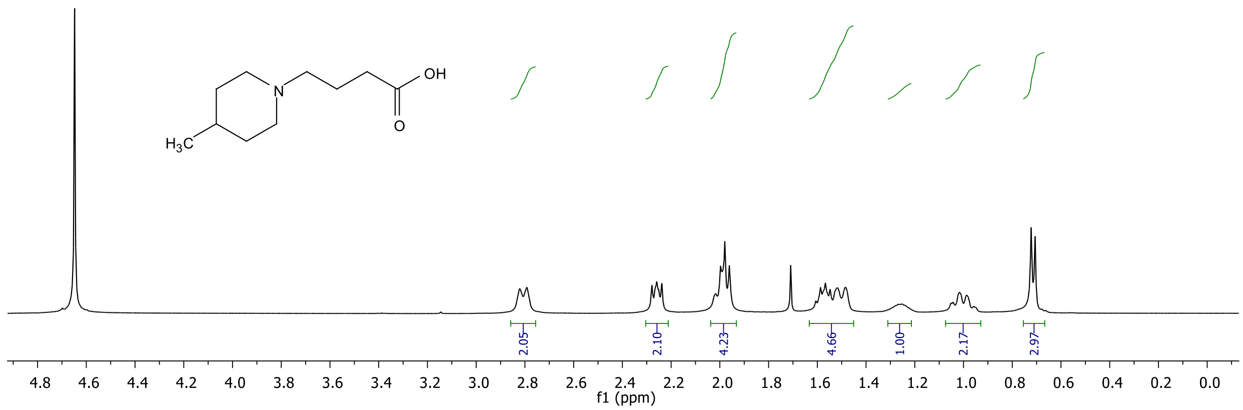

Figure 7S. ^{1}H NMR (400 MHz, CD_3OD) of 4-(4-methylpiperidin-1-yl)butanoic acid (**7d**).

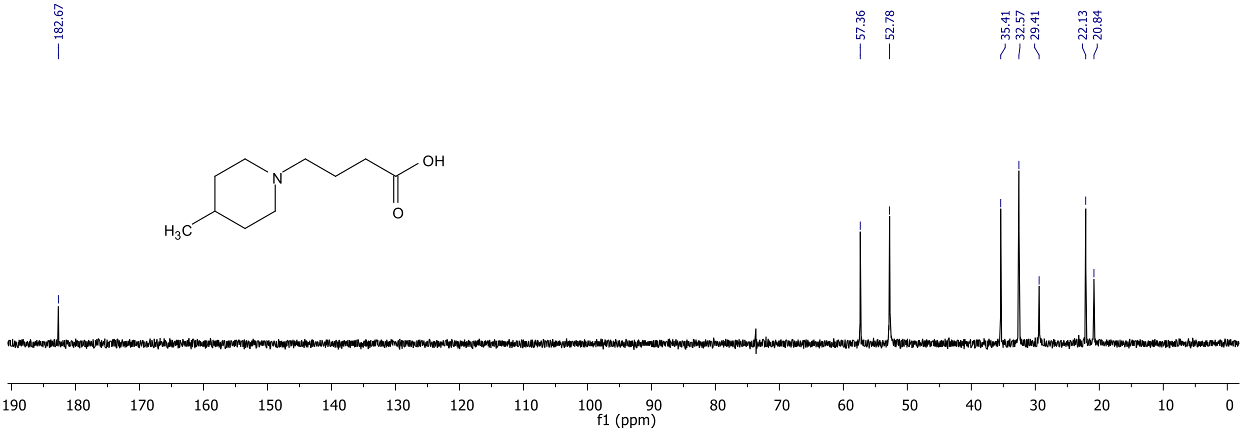

Figure 8S. ^{13}C NMR (100 MHz, CD_3OD) of 4-(4-methylpiperidin-1-yl)butanoic acid (**7d**).

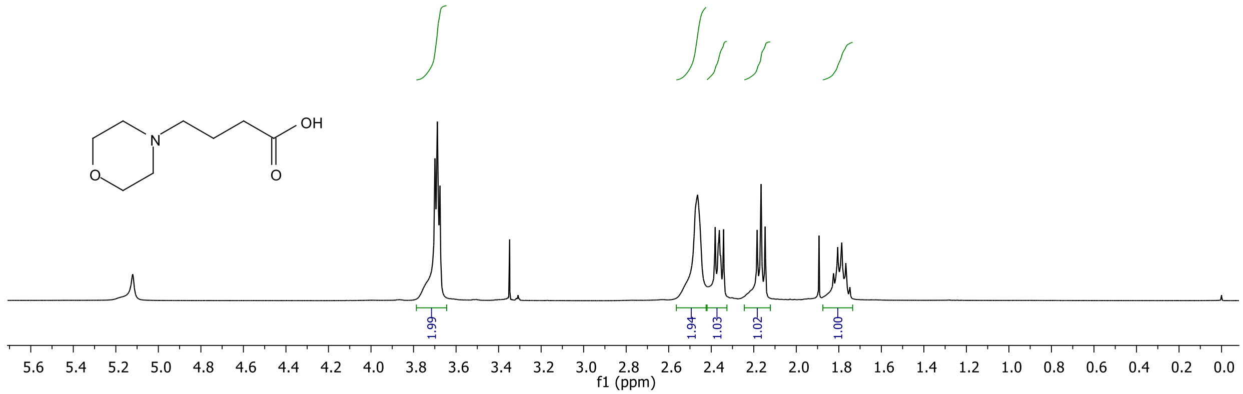

Figure 9S. ^{1}H NMR (400 MHz, CD_3OD) of 4-morpholinobutanoic acid (**7e**).

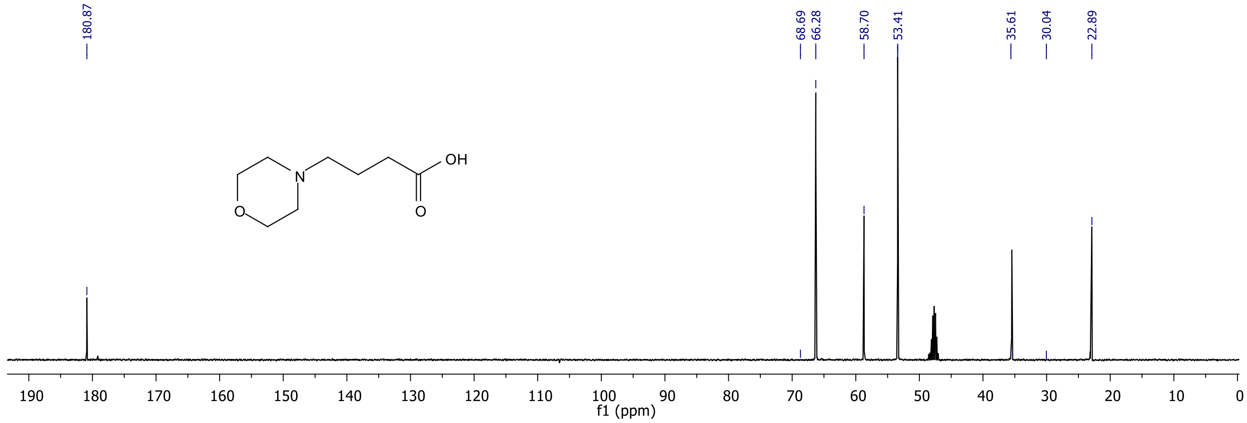

Figure 10S. ^{13}C NMR (100 MHz, CD_3OD) of 4-morpholinobutanoic acid (**7e**).

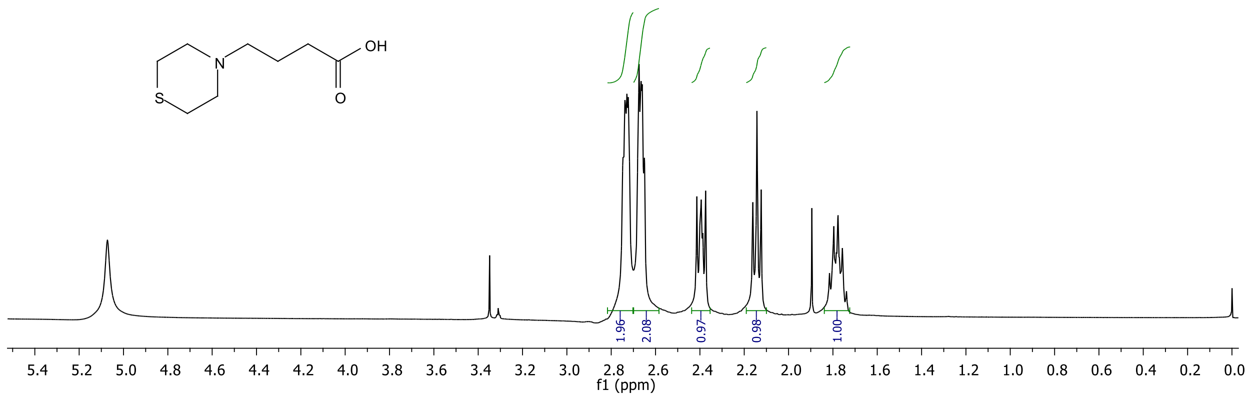

Figure 11S. ^{1}H NMR (400 MHz, CD_3OD) of 4-thiomorpholinobutanoic acid (**7f**).

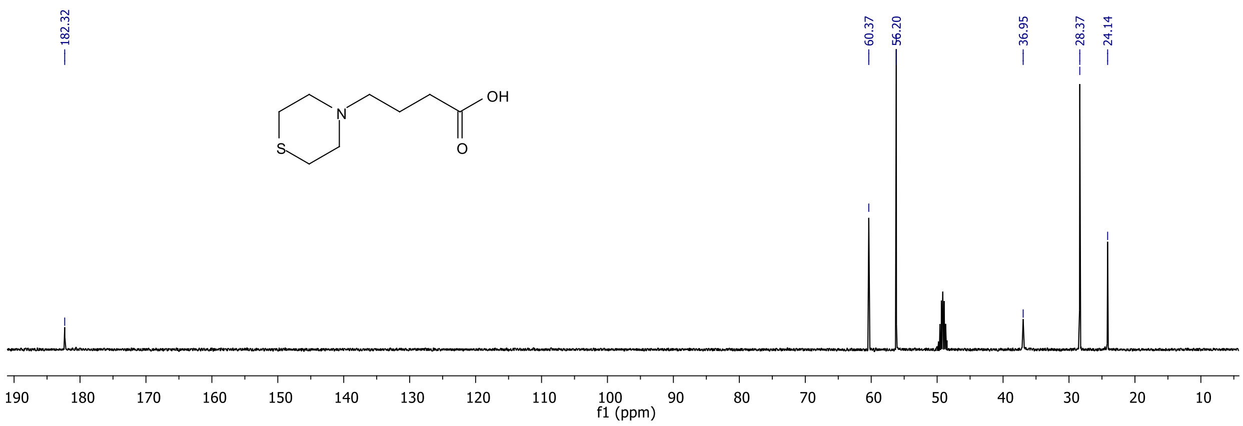

Figure 12S. ^{13}C NMR (100 MHz, CD_3OD) of 4-thiomorpholinobutanoic acid (**7f**).

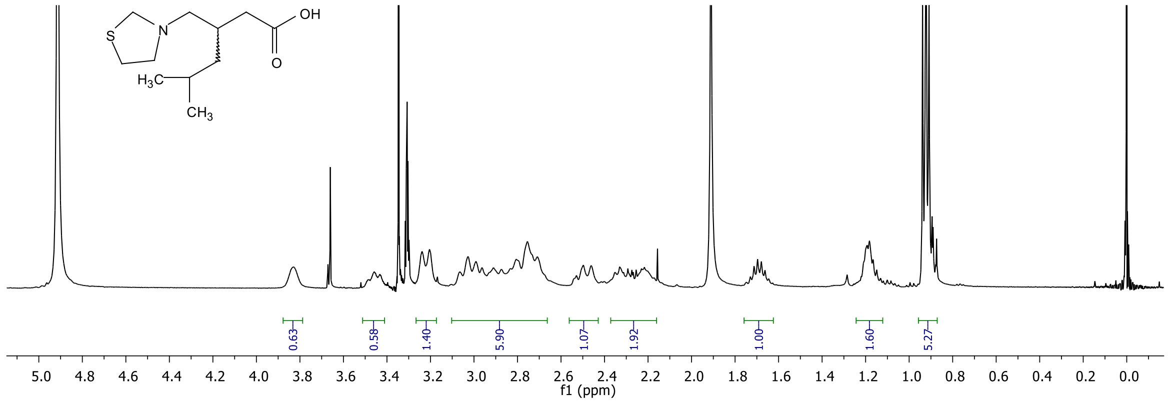

Figure 13S. ^{1}H NMR (400 MHz, CD_3OD) of 5-methyl-3-(thiazolidin-3-ylmethyl)hexanoic acid (**8a**).

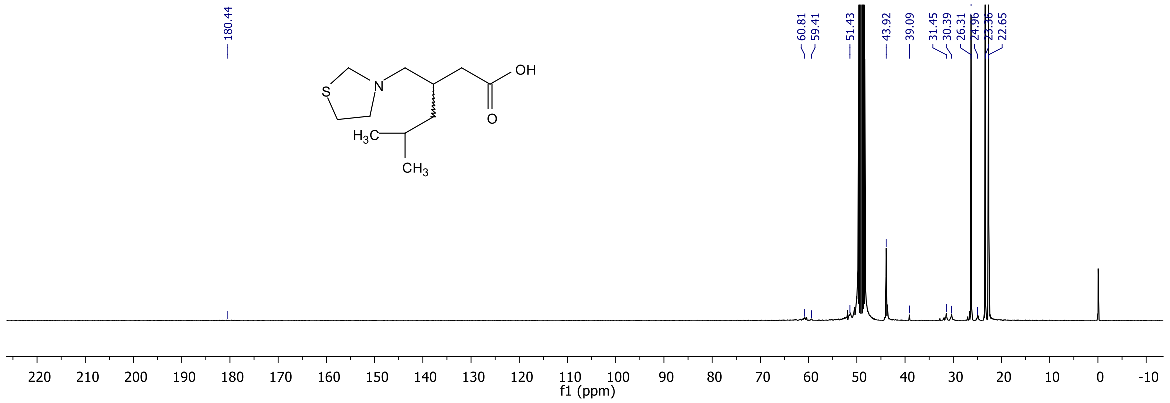

Figure 14S. ^{13}C NMR (100 MHz, CD_3OD) of 5-methyl-3-(thiazolidin-3-ylmethyl)hexanoic acid (**8a**).

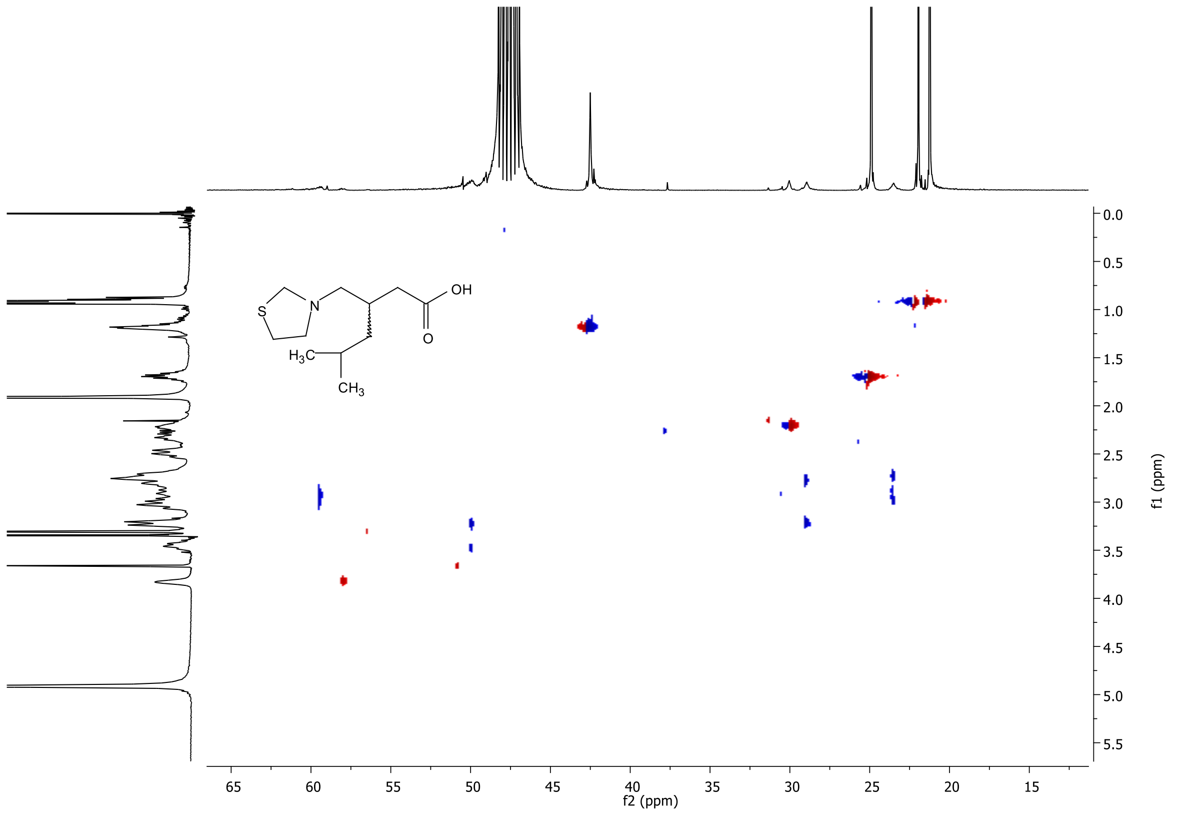

Figure 15S. 2D NMR (HETCOR 400 MHz, CD_3OD) of 5-methyl-3-(thiazolidin-3-ylmethyl)hexanoic acid (**8a**).

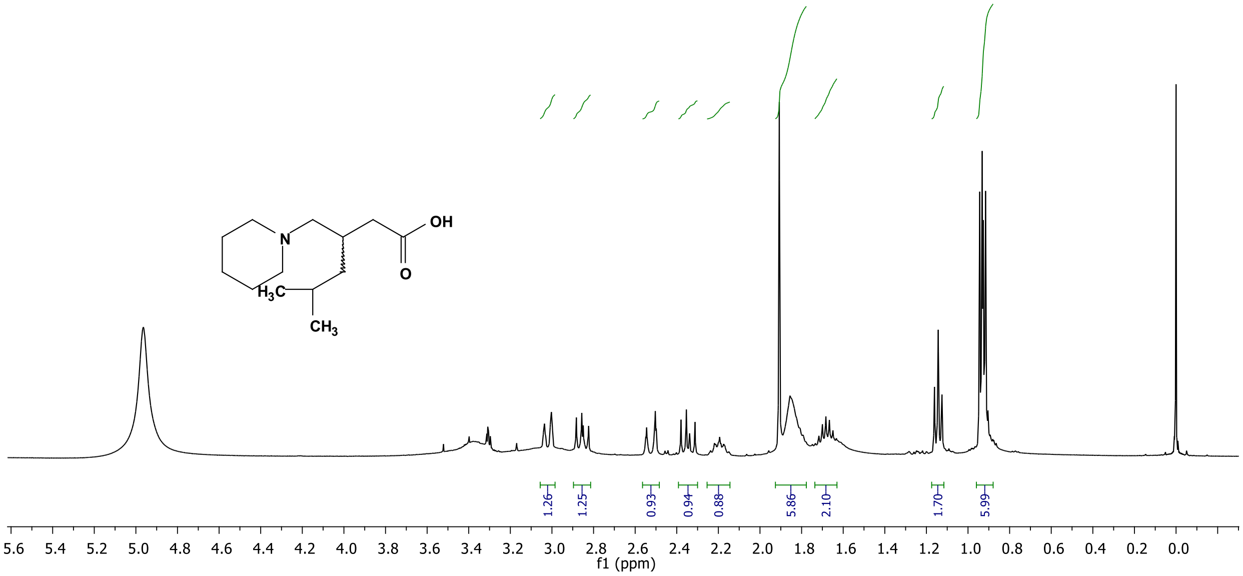

Figure 16S. ^1H NMR (400 MHz, CD_3OD) of 5-methyl-3-(piperidin-1-ylmethyl)hexanoic acid (**8b**).

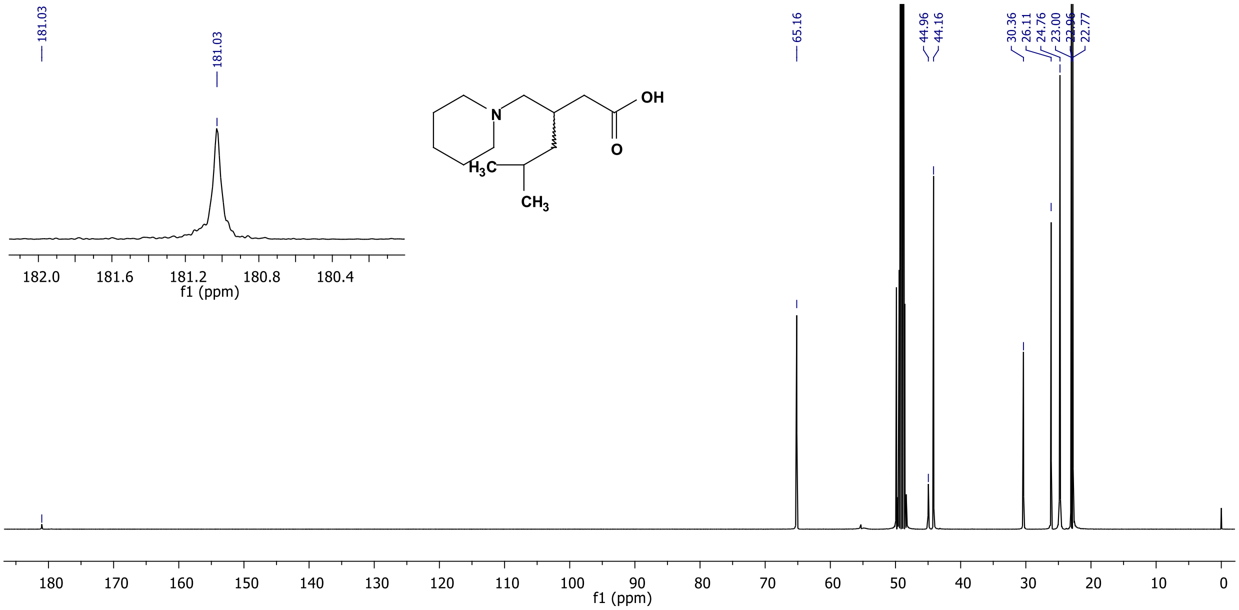

Figure 17S. ^{13}C NMR (100 MHz, CD_3OD) of 5-methyl-3-(piperidin-1-ylmethyl)hexanoic acid (**8b**).

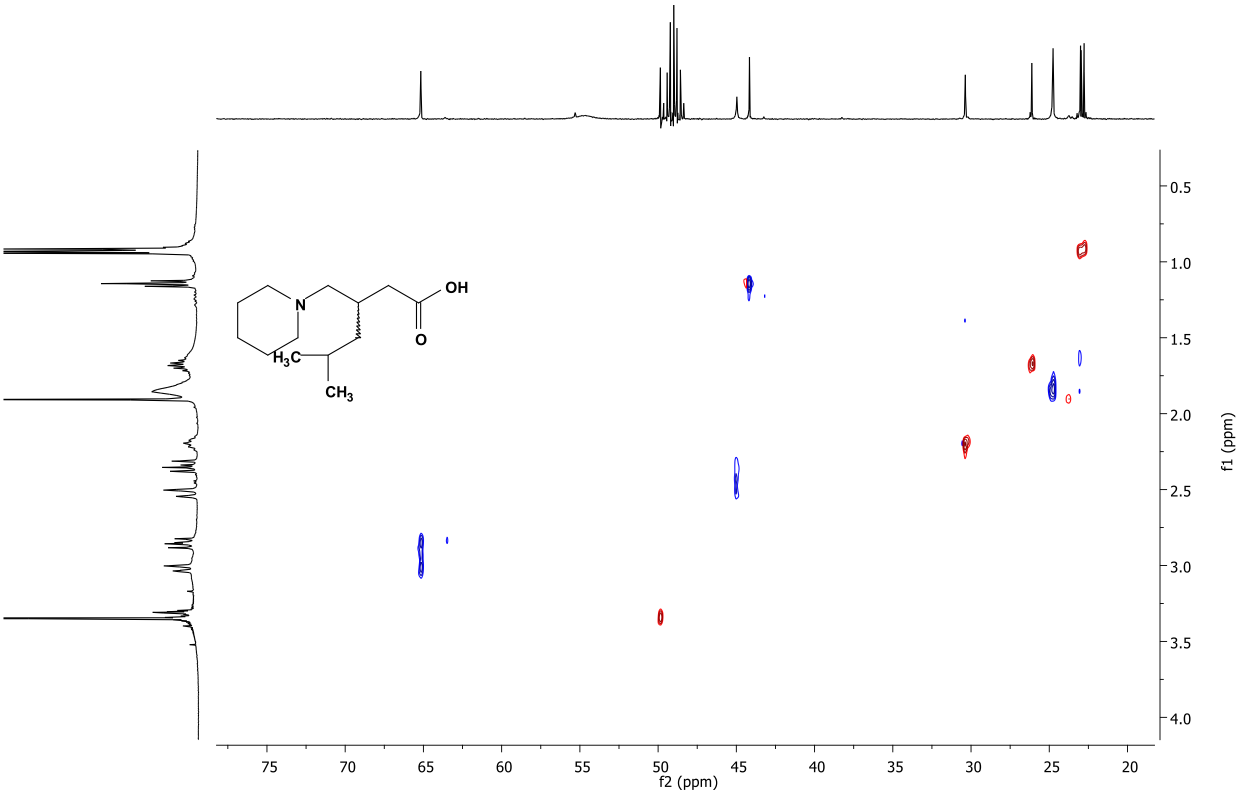

Figure 18S. 2D NMR (HETCOR 400 MHz, CD_3OD) of 5-methyl-3-(piperidina-1-ylmethyl) hexanoic acid (**8b**).

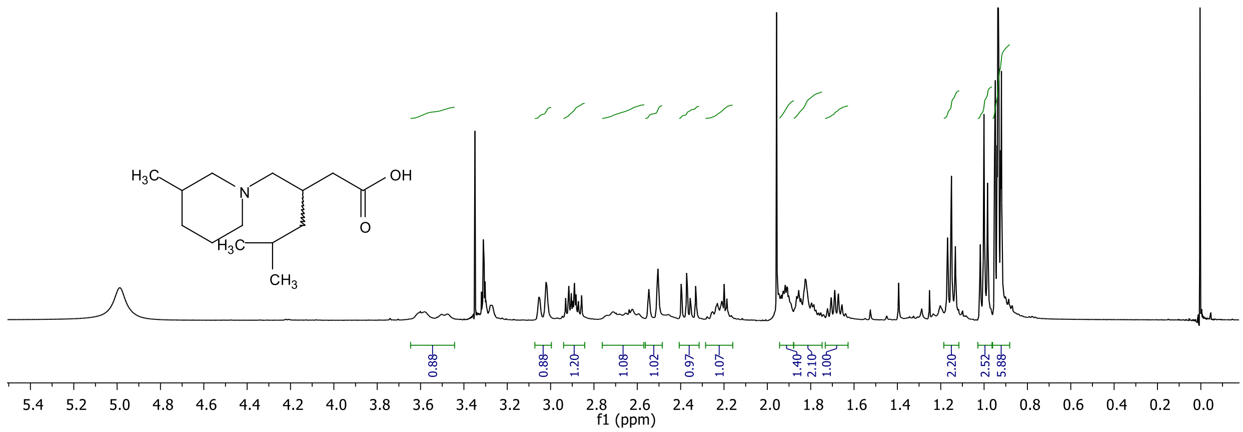

Figure 19S. ^{1}H NMR (400 MHz, CD_3OD) of 5-methyl-3-((3-methylpiperidin-1 yl)methyl)hexanoic acid (**8c**).

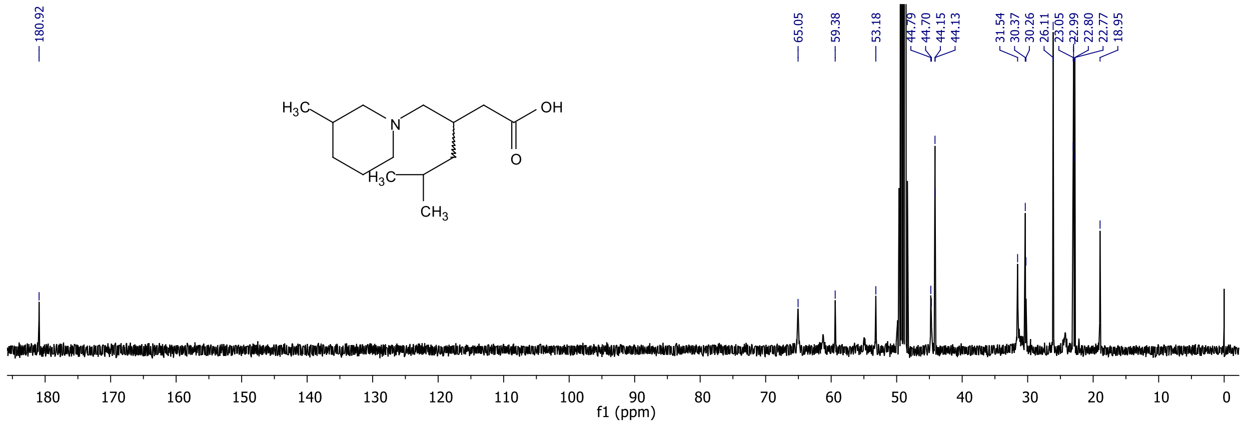

Figure 20S. ^{13}C NMR (100 MHz, CD_3OD) of 5-methyl-3-((3-methylpiperidin-1-yl)methyl)hexanoic acid (**8c**).

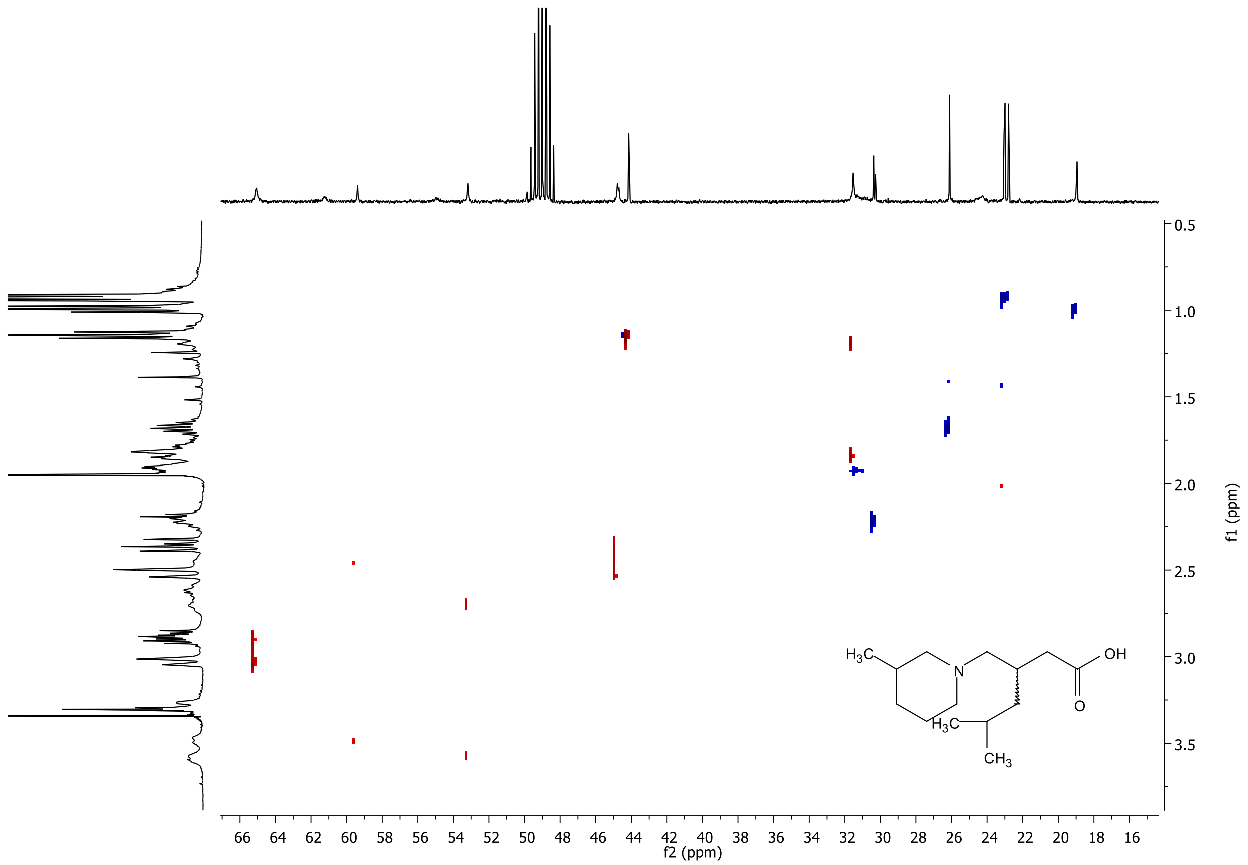

Figure 21S. 2D NMR (HETCOR 400 MHz, CD_3OD) of 5-methyl-3-((3-methylpiperidin-1-yl)methyl)hexanoic acid (**8c**).

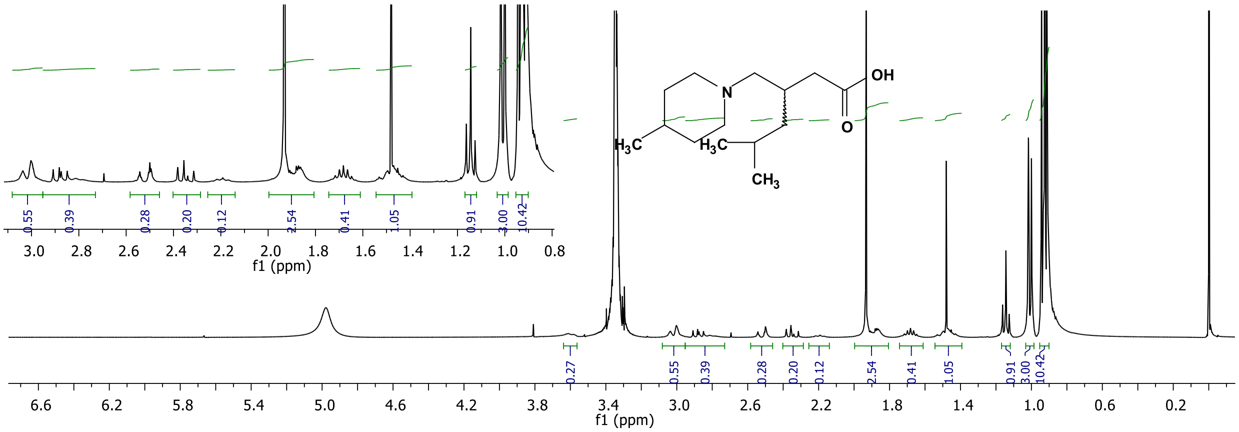

Figure 22S. ^{1}H NMR (400 MHz, CD_3OD) of 5-methyl-3-((4-methylpiperidin-1-yl)methyl)hexanoic acid (**8d**).

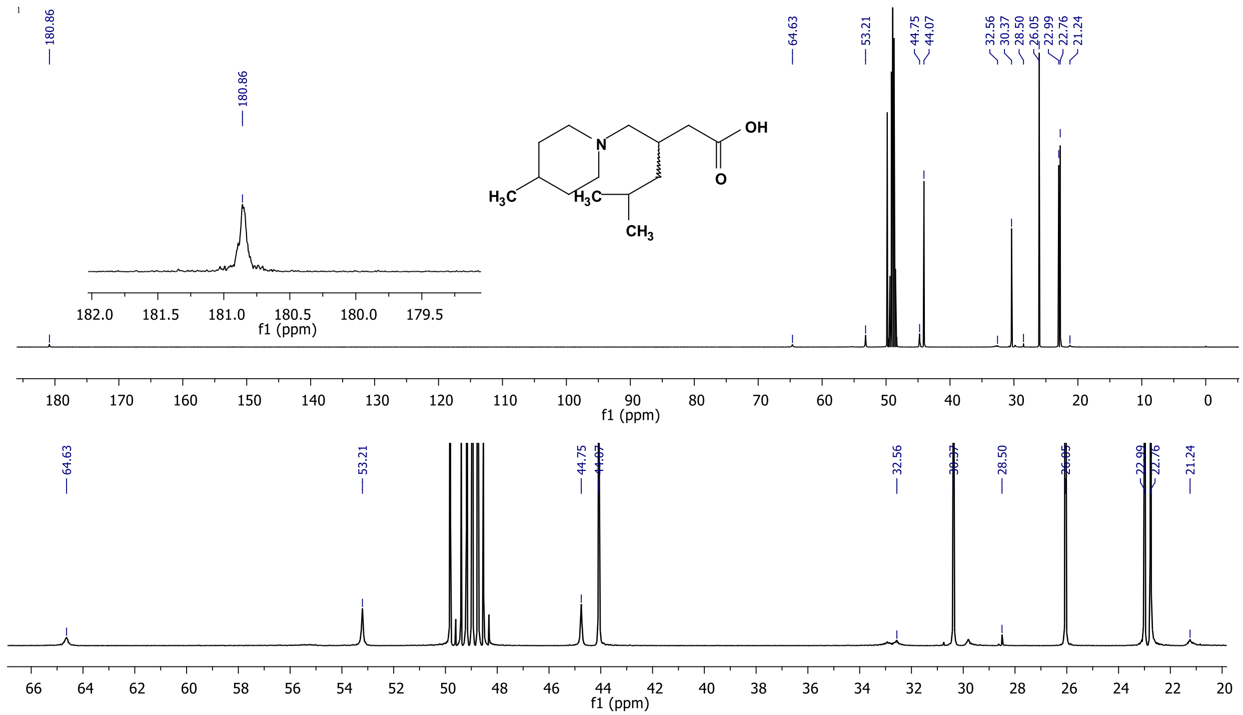

Figure 23S. ^{13}C NMR (100 MHz, CD_3OD) of 5-methyl-3-((4-methylpiperidin-1-yl)methyl)hexanoic acid (**8d**).

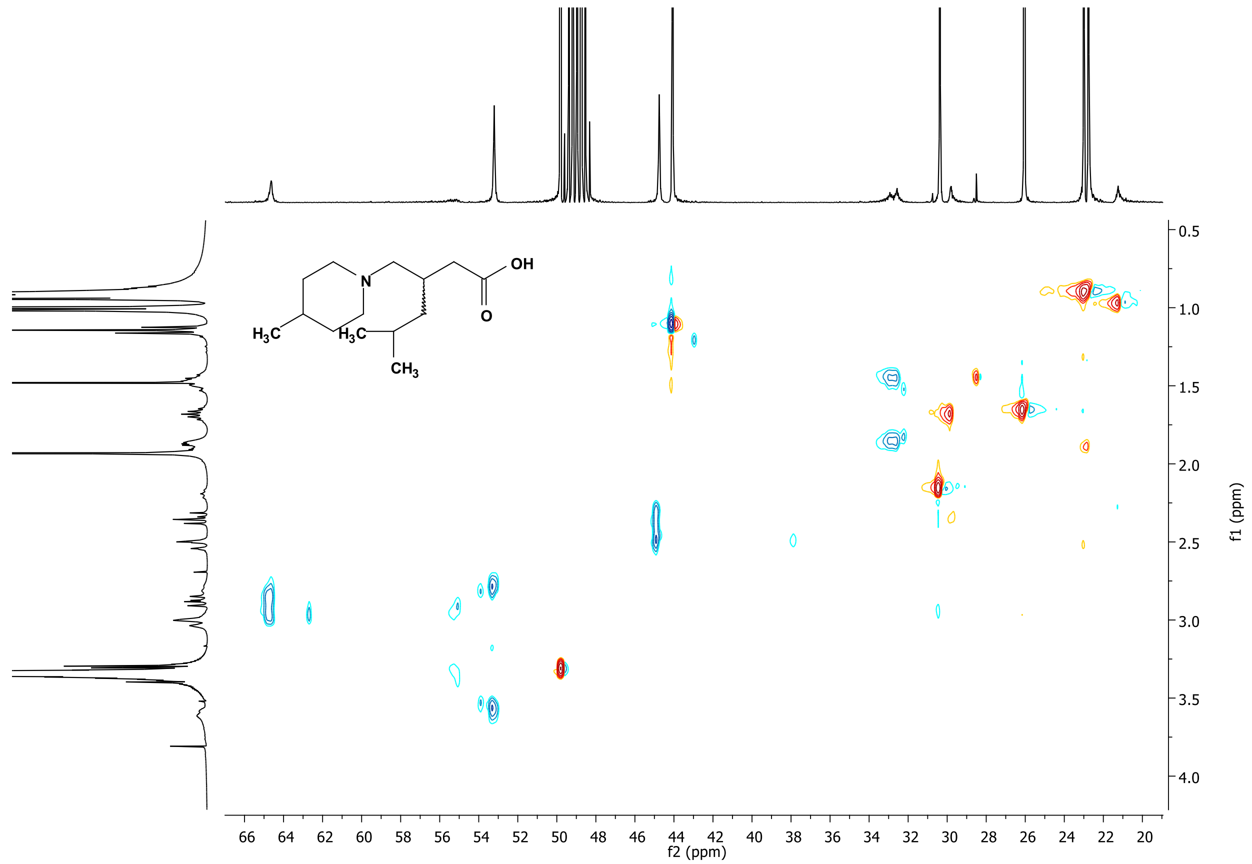

Figure 24S. 2D NMR (HETCOR 400 MHz, CD_3OD) of 5-methyl-3-((4-methylpiperidin-1-yl)methyl)hexanoic acid (**8d**).

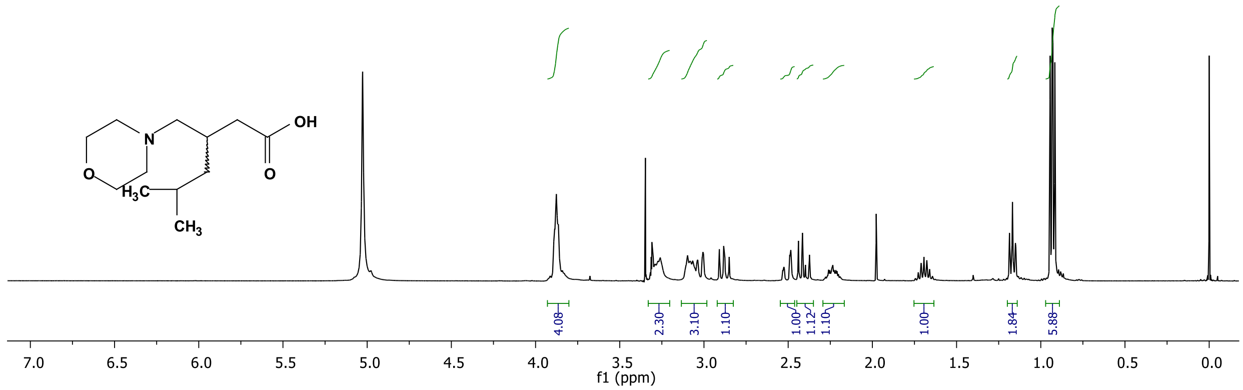

Figure 25S. ^{1}H NMR (400 MHz, CD_3OD) of 5-methyl-3-(morpholinomethyl) hexanoic acid (**8e**).

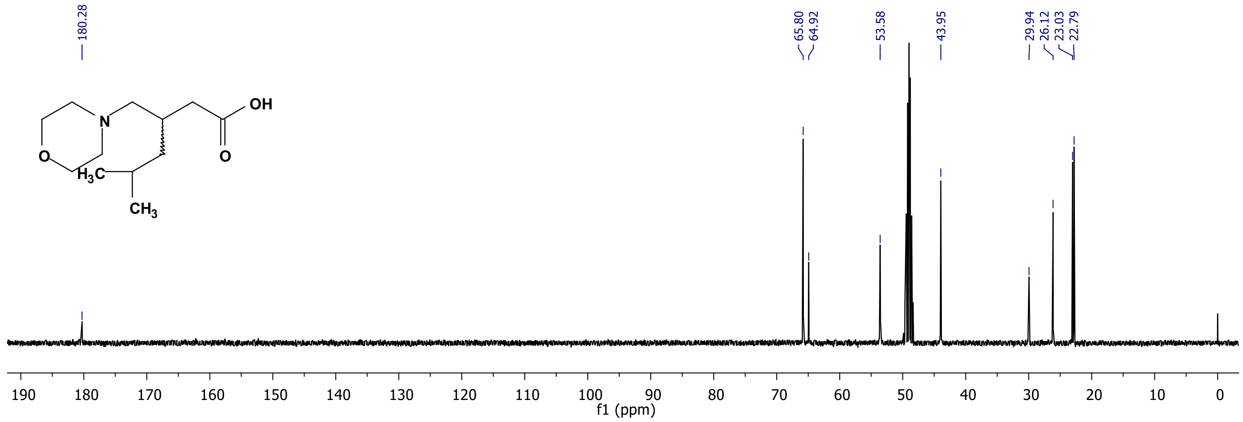

Figure 26S. ^{13}C NMR (100 MHz, CD_3OD) of 5-methyl-3-(morpholinomethyl)hexanoic acid (**8e**).

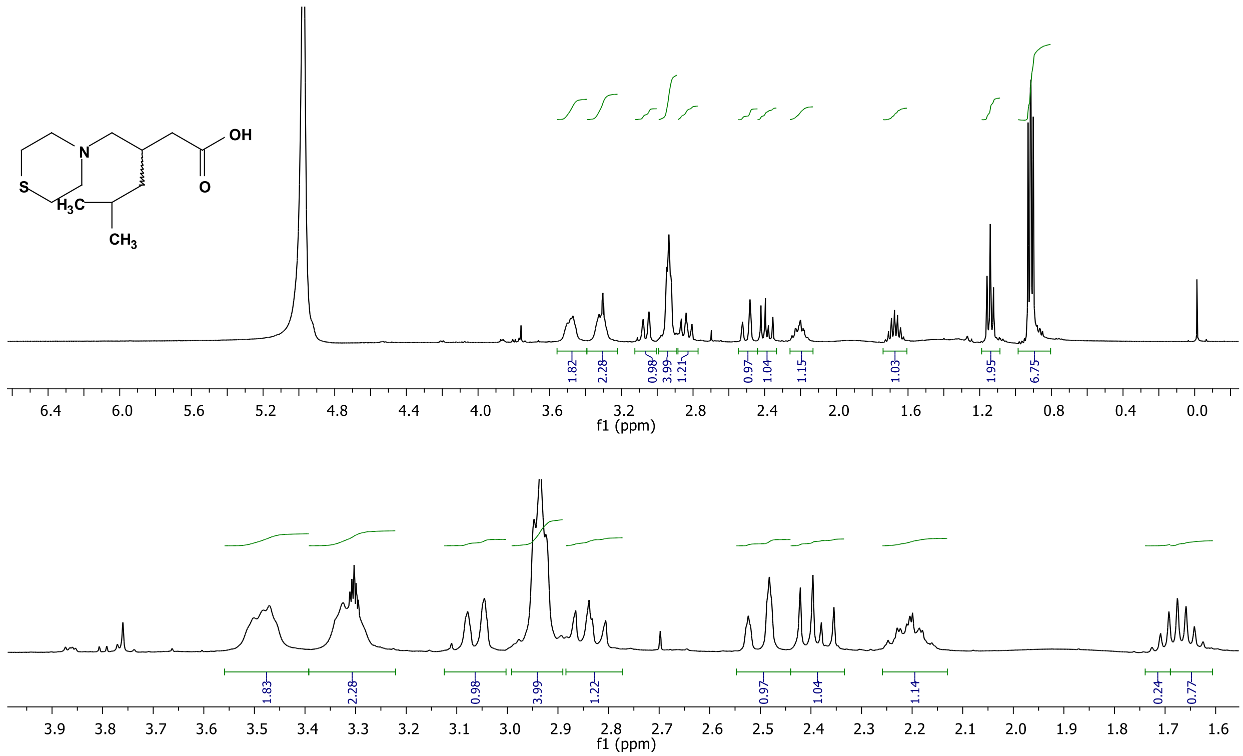

Figure 27S. ^{1}H NMR (400 MHz, CD_3OD) of 5-methyl-3-(thiomorpholinomethyl)hexanoic acid (**8e**).

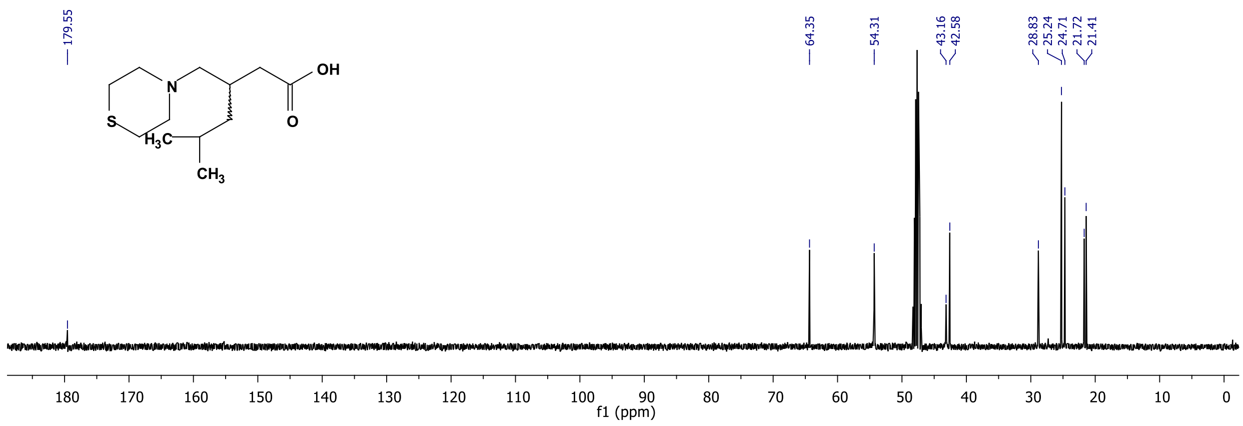

Figure 28S. ^{13}C NMR (100 MHz, CD_3OD) of 5-methyl-3-(thiomorpholinomethyl)hexanoic acid (**8f**).

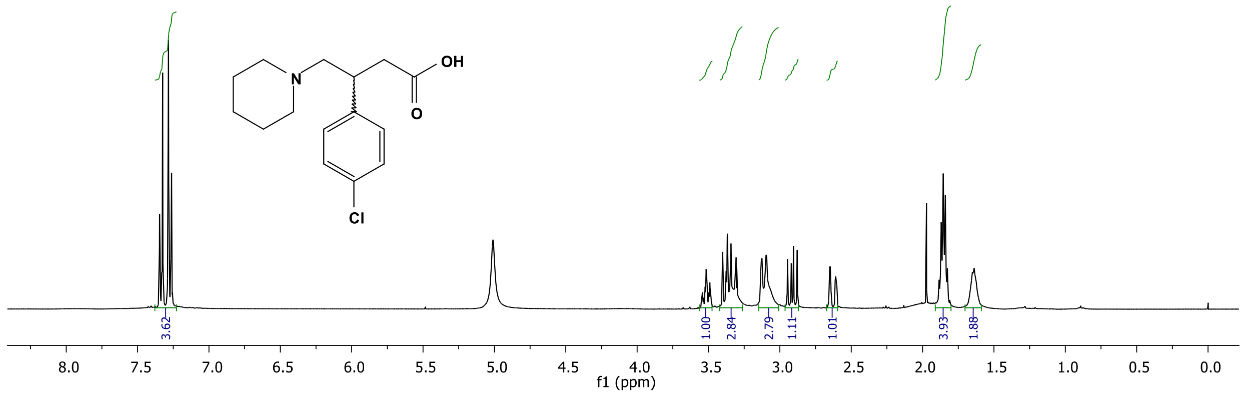

Figure 29S. ^{1}H NMR (400 MHz, CD_3OD) of 3-(4-chlorophenyl)-4-(piperidin-1-yl)butanoic acid (**9b**).

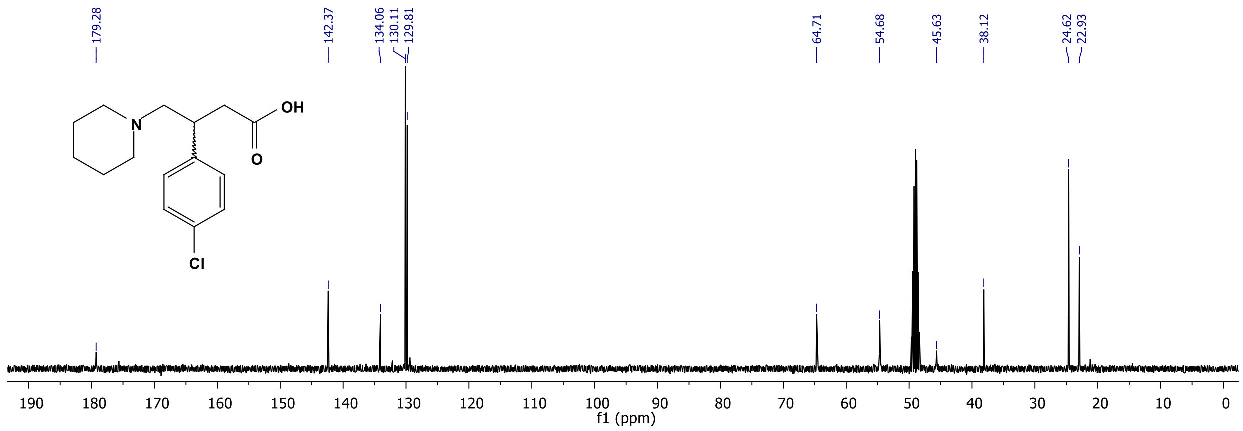

Figure 30S. ^{13}C NMR (100 MHz, CD_3OD) of 3-(4-chlorophenyl)-4-(piperidin-1-yl)butanoic acid (**9b**).

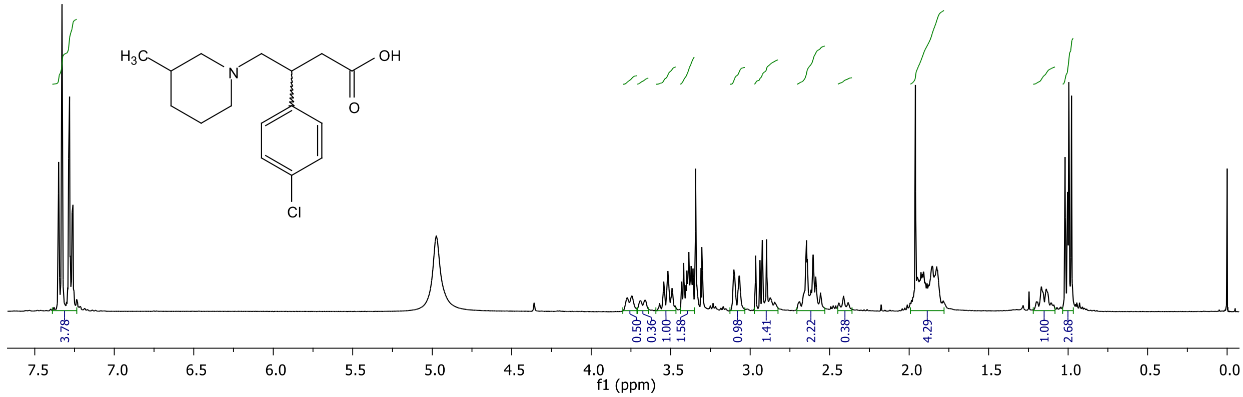

Figure 31S. ¹H NMR (400 MHz, CD_3OD) of 3-(4-chlorophenyl)-4-(3-methylpiperidin-1-yl)butanoic acid (**9c**).

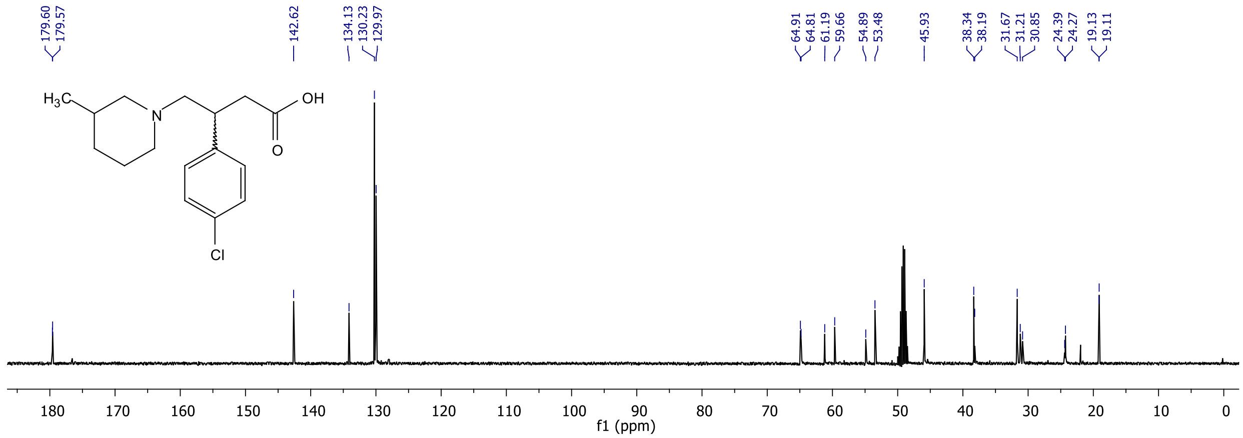

Figure 32S. ¹³C NMR (100 MHz, CD_3OD) of 3-(4-chlorophenyl)-4-(3-methylpiperidin-1-yl)butanoic acid (**9c**).

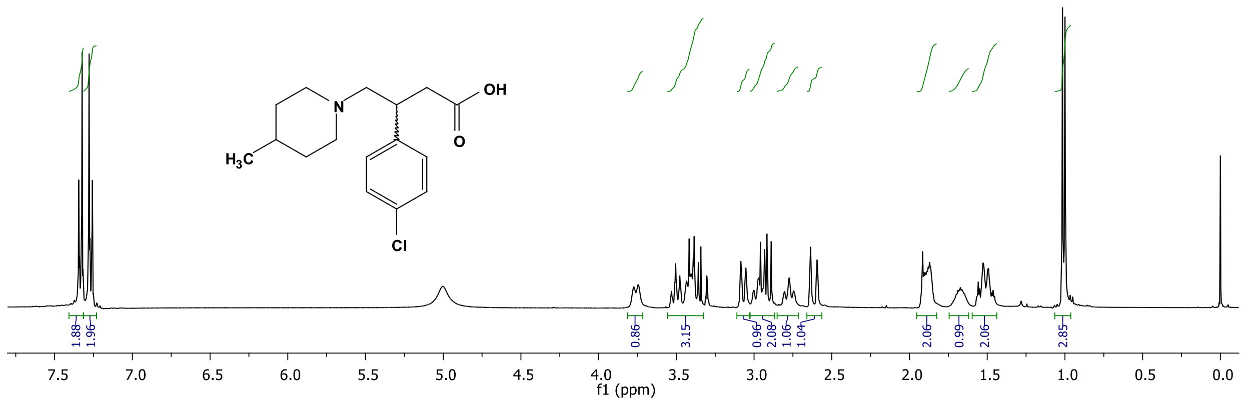

Figure 33S. ¹H NMR (400 MHz, CD_3OD) of 3-(4-chlorophenyl)-4-(4-methylpiperidin-1-yl) butanoic acid (**9d**).

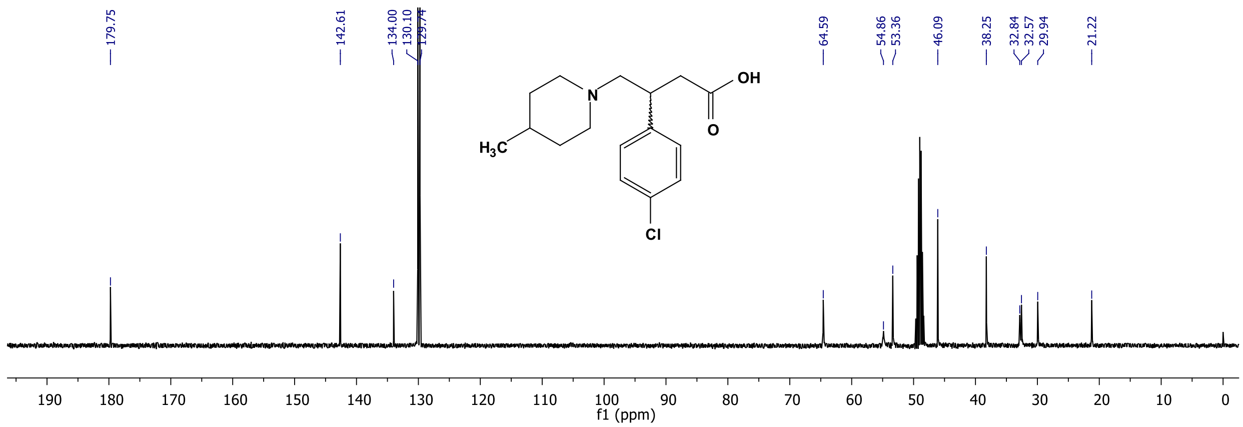

Figure 34S. ^{13}C NMR (100 MHz, CD_3OD) of 3-(4-chlorophenyl)-4-(4-methylpiperidin-1-yl) butanoic acid (**9d**).

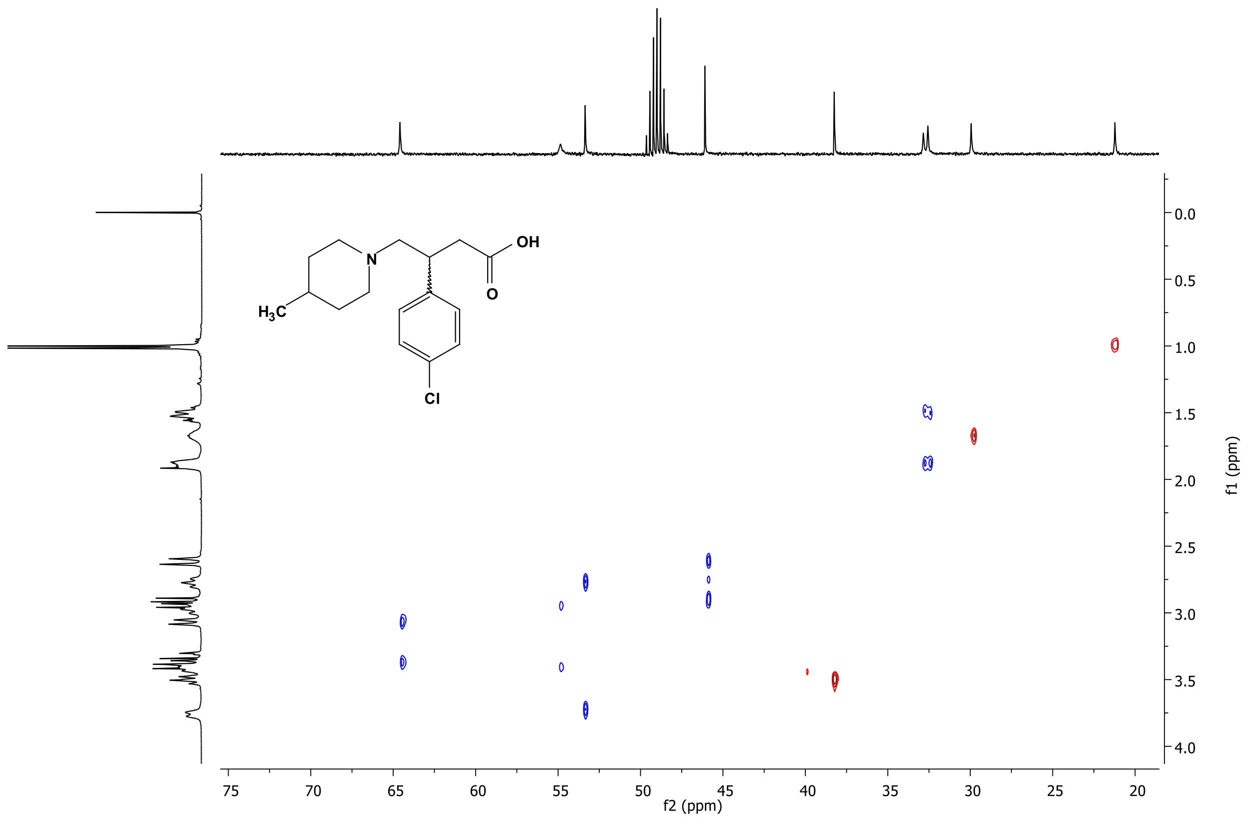

Figure 35S. 2D NMR (HETCOR 400 MHz, CD_3OD) of 3-(4-chlorophenyl)-4-(4-methylpiperidin-1-yl)butanoic acid (**9d**).

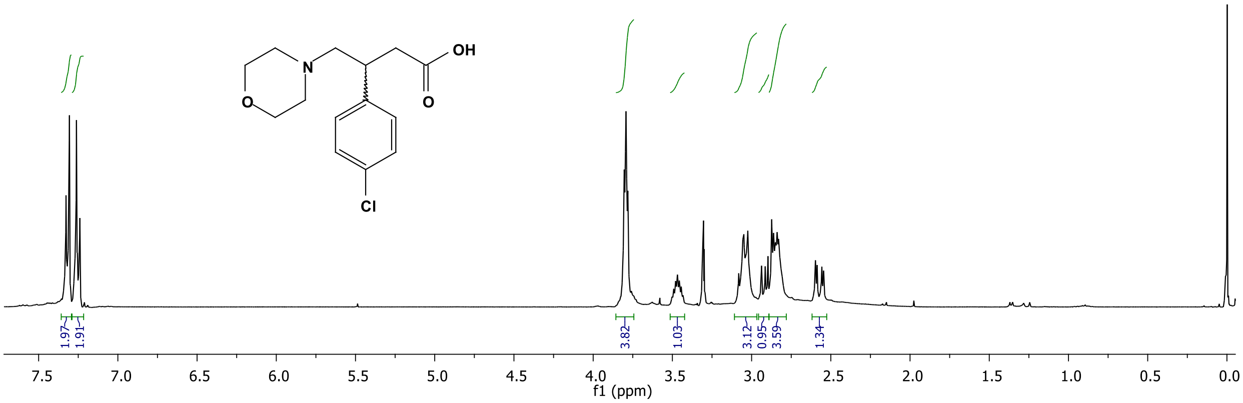

Figure 36S. ^{1}H NMR (400 MHz, CD_3OD) of 3-(4-chlorophenyl)-4-morpholinobutanoic acid (**9e**).

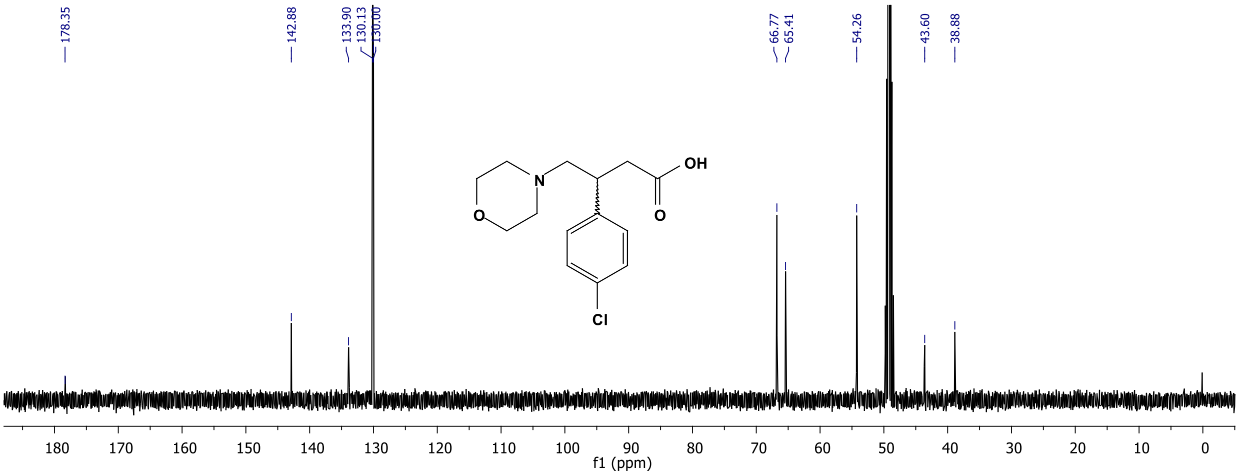

Figure 37S. ^{13}C NMR (100 MHz, CD_3OD) of 3-(4-chlorophenyl)-4-morpholinobutanoic acid (**9e**).

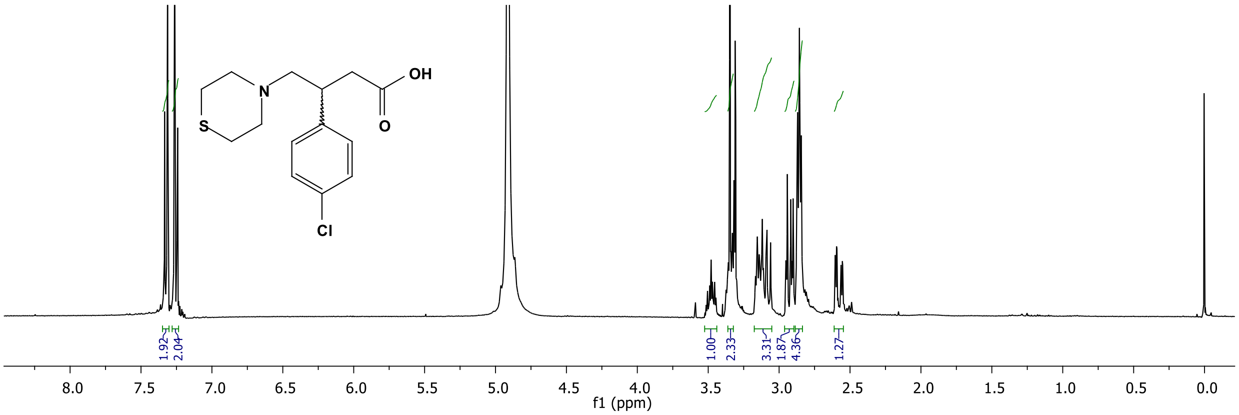

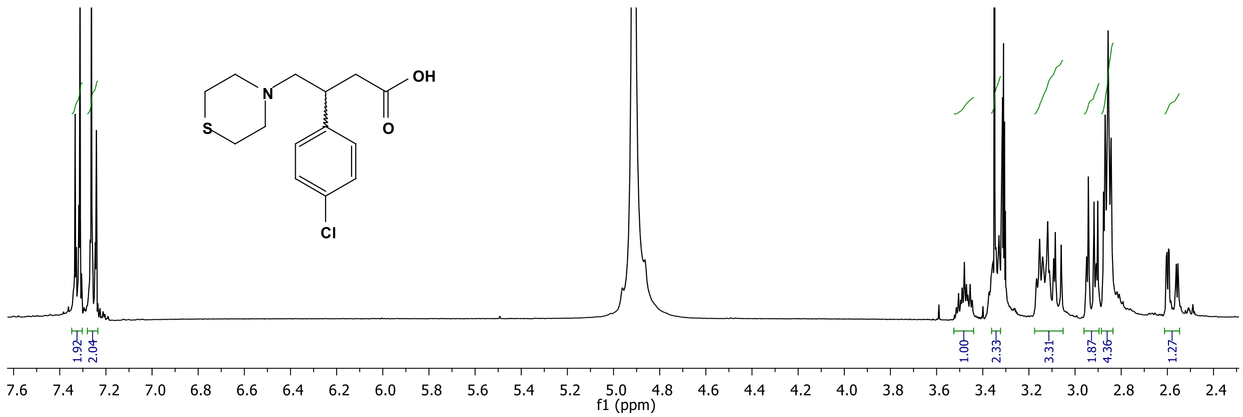

Figure 38S. ^{1}H NMR (400 MHz, CD_3OD) of 3-(4-chlorophenyl)-4-thiomorpholinobutanoic acid (**9f**).

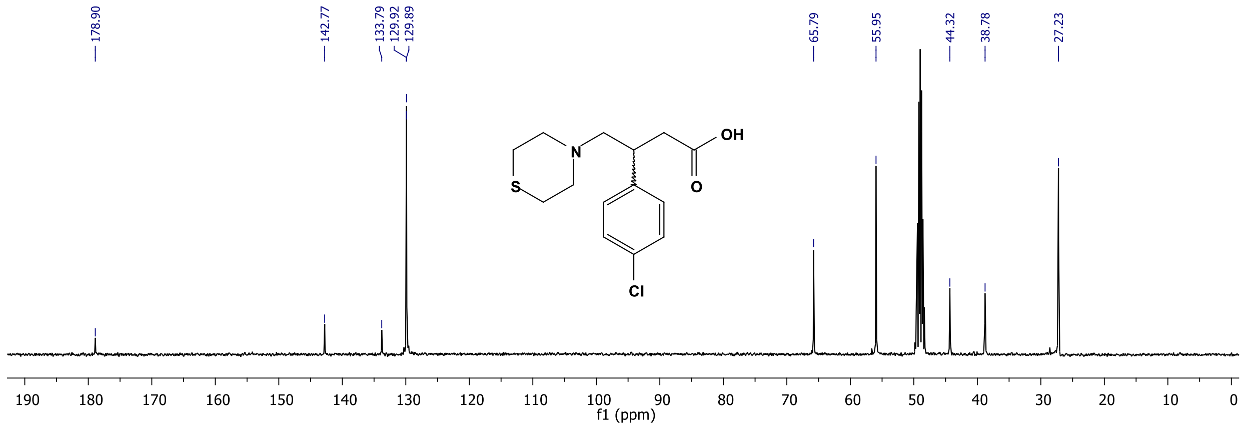

Figure 39S. ^{13}C NMR (100 MHz, CD_3OD) of 3-(4-chlorophenyl)-4-thiomorpholinobutanoic acid (**9f**).

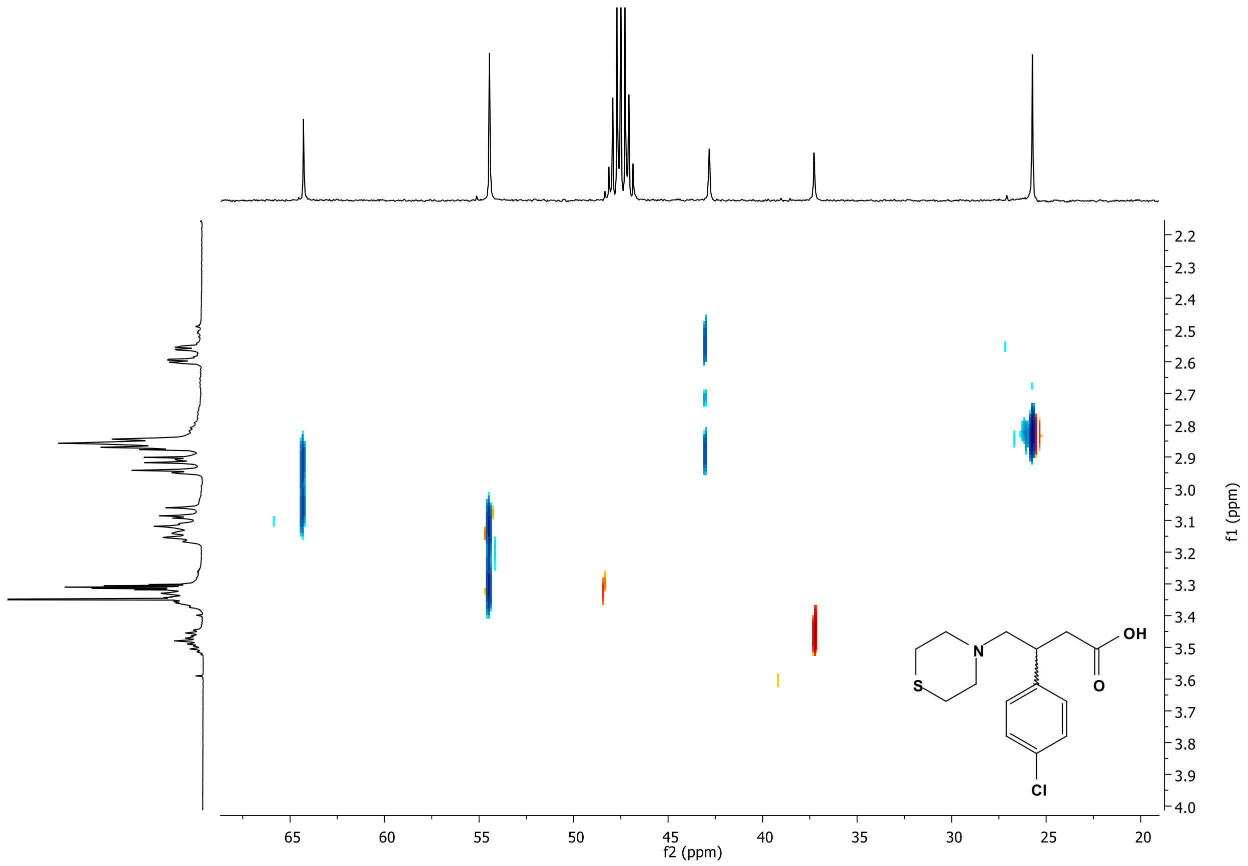

Figure 40S. 2D NMR (HETCOR 400 MHz, CD_3OD) of 3-(4-chlorophenyl)-4-thiomorpholinobutanoic acid (**9f**).

Computational Details

PF ------------MSNKTNASLMKRREAAVPRGVGQIHP-IFAESAKNATVTDVEGREFID
EC ---------------NSNKELMQRRSQAIPRGVGQIHPI-FADRAENCRVWDVEGREYLD
HS -FDYDGPLMKTEVPGPRSQELMKQLNII--QNAEAVHFFCNYEESRGNYLVDVDGNRMLD
JB -FDYDGPLMKTEVPGPRSRELMKQLNII--QNAEAVHFFCNYEESRGNYLVDVDGNRMLD

PF FAGGIAVLNTGHLHPKIIAAVTEQLNKLTH---TCFQVLAYEPYVELCEKVNAK-VPGDF
EC FAGGIAVLNTGHLHPKVVAAVEAQLKKLSH---TCFQVLAYEPYLELCEI-MNQKVPGDF
HS LYSQISSVPIGYSHPALLKLIQQPQNASMFVNRPALGILPPENFVEKLRQSLLSVAPKGM
JB LYSQISSIPIGYSHPALVKLVQQPQNVSTFINRPALGILPPENFVEKLRESLLSVAPKGM

PF AKKTLLVTTGSEA-------------------------VENAVKIARATTGRAGVIAFT
EC AKKTLLVTTGSEAVENA-------------------------VKIARAATKRSGTIAFS
HS -SQLITMACGSCSNENALKTIFMWYRSKERGQRGFSQEELETCMINQAPGCPDYSILSFM
JB -SQLITMACGSCSNENAFKTIFMWYRSKERGESAFSKEELETCMINQAPGCPDYSILSFM

PF GAYHGRTMMTLGLTGKVVPYSAGMGLM--P-GGIFRALYPNELHGVS-V---DDSIAS-I
EC GAYHGRTHYTLALTGKVNPYSAGMGL---MPGHVYRALYPCPLHGI----SEDDAIASI-
HS GAFHGRTMGCLATTHSKAIHKIDIPSFDWPIAPFPRLKYPLEEFVKENQQEEARCLEEVE
JB GAFHGRTMGCLATTHSKAIHKIDIPSFDWPIAPFPRLKYPLEEFVKENQQEEARCLEEVE

PF ERIFKNDAEPRDIAAIIIEPVQGEGGFYVAPKAFMKRLRELCDKHGILLIADEVQTGAGR
EC HRIFKNDAAPEDIAAIVIEPVQGEGGFYASSPAFMQRLRALCDEHGIMLIADEVQSGAGR
HS DLIVKYRKKKKTVAGIIVEPIQSEGGDNHASDDFFRKLRDIARKHGCAFLVDEVQTGGGC
JB DLIVKYRKKKKTVAGIIVEPIQSEGGDNHASDDFFRKLRDISRKHGCAFLVDEVQTGGGS

PF TGTFFAMEQMGVAA--DLTTFAKSI-AGGFPLAGVCGKAEYMDAIAPGGLGGTYAGSPIA
EC TGTLFAMEQMGVAP--DLTTFAKSI-AGGFPLAGVTGRAEVMDAVAPGGLGGTYAGNPIA
HS TGKFWAHEHWGLDDPADVMTFSKKMMTGGFFH-----K-EEFRPNAPYRIFNTWLGDPSK
JB TGKFWAHEHWGLDDPADVMTFSKKMMTGGFFH-----K-EEFRPNAPYRIFNTWLGDPSK

PF CAAALAVMEVFEEEHLLDRCKAVGERLVTGLKAIQAKYPVI-GEVRALGAMIALELFEDG
EC CVAALEVLKVFEQENLLQKANDLGQKLKDGLLAIAEKHPEI-GDVRGLGAMIAIELFEDG
HS NLLLAEVINIIKREDLLNNAAHAGKALLTGLLDLQARYPQFISRVRGRGTFCSFDT----
JB NLLLAEVINIIKREDLLSNAAHAGKVLLTGLLDLQARYPQFISRVRGRGTFCSFDT----

PF DSHKPNAAAVASVVAKARDKGLILLSCGTYGNVLRVLVPLTSPDEQLDKGLAIIEECFSEL-
EC DHNKPDAKLTAEIVARARDKGLILLSCGPYYNVLRILVPLTIEDAQIRQGLEIISQCFDEAK
HS ----PDDSIRNKLILIARNKGVVLGGCGDKSIRFRPTLVFRDHHA--HLFLNIFSDILADFK
JB ----PDESIRNKLISIARNKGVMLGGCGDKSIRFRPTLVFRDHHA--HLFLNIFSDILADFK

Figure 41S. Alignment of *pseudomonas fluorencens* (**PF**), *human* (HS), *E. coli* (EC) and *wild boar* (JB). Red and blue color letters correspond to the residues of the chain A and chain B respectively, that interact with VGB **3** in the 1ohv crystal structure.

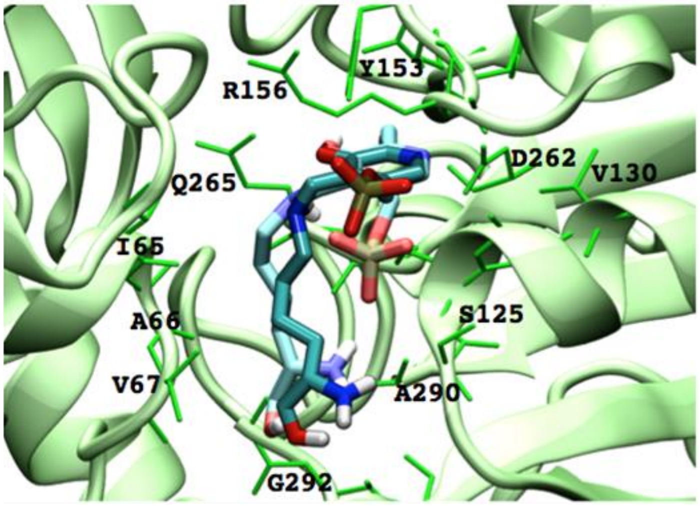

Figure 42S. Validation of the molecular docking calculation for the pseudomonas model. Ligand in the PDB:ID 3r4t crystal structure was reproduced with a RMSD of 1.7 Å. Ligand experimental (opaque color) and calculated conformation (shiny color) are displayed as sticks representation respectively. Residues within 4.0 Å of both ligands are shown as thin sticks.

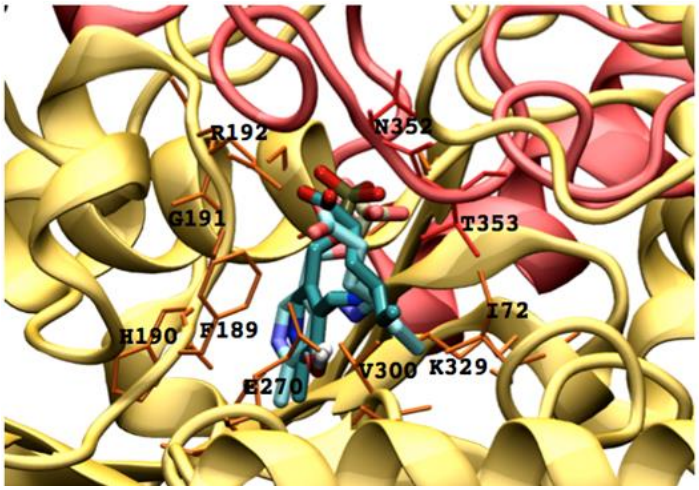

Figure 43S. Validation of the molecular docking calculation for the *human* model. Ligand in the PDB:ID 1ohw crystal structure was reproduced with a RMSD of 1.3 Å. Ligand experimental (opaque color) and calculated conformation (shiny color) are displayed as sticks representation respectively. Residues within 4.0 Å of both ligands are shown as thin sticks.

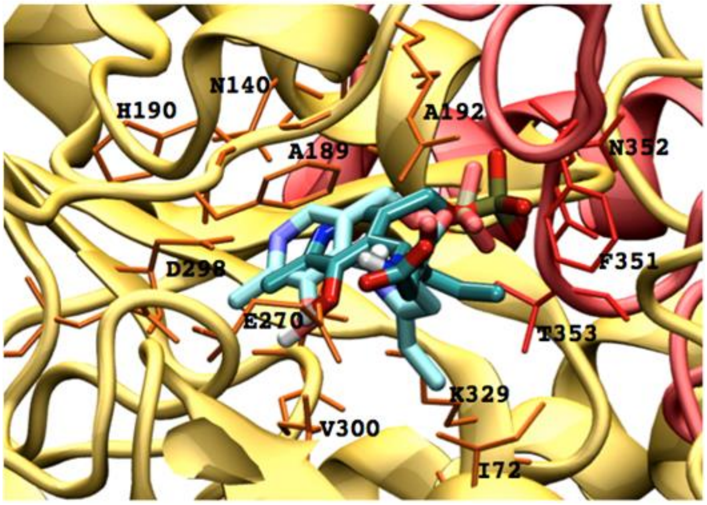

Figure 44S. Validation of the molecular docking calculation for the *human* model. Ligand in the PDB:ID 1ohy crystal structure was reproduced with a RMSD of 1.8 Å. Ligand experimental (opaque color) and calculated conformation (shiny color) are displayed as sticks representation respectively. Residues within 4.0 Å of both ligands are shown as thin sticks.

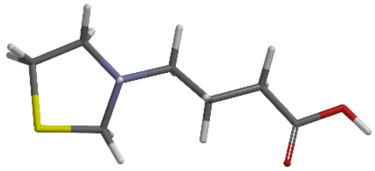
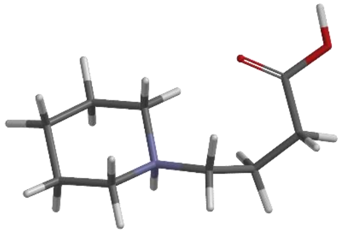
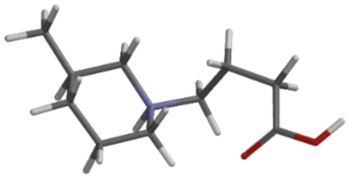

7a **7b** **7c**

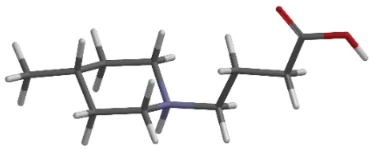
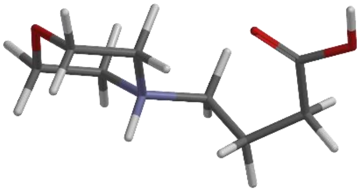
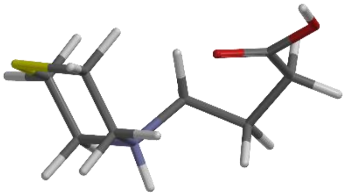

7d **7e** **7f**

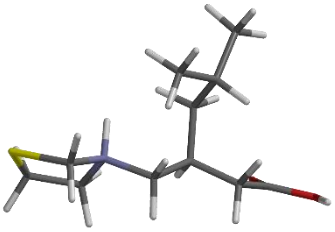
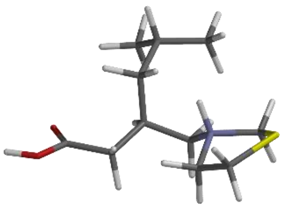
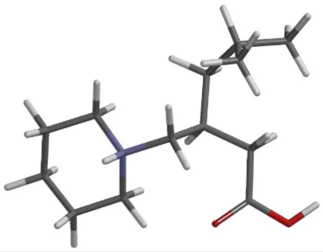

(*R*)-8a **(*S*)-8a** **(*R*)-8b**

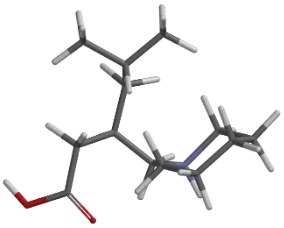
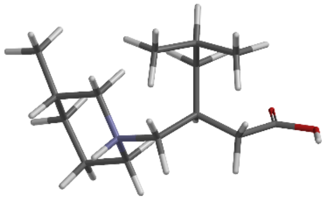
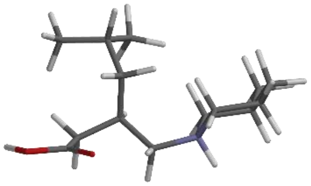

(*S*)-8b **(*R*)-8c** **(*S*)-8c**

Figure 45S. Optimized structures of all GABA analogues, VPNa and VGB.

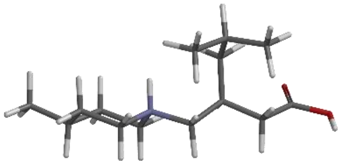

(*R*)-8d

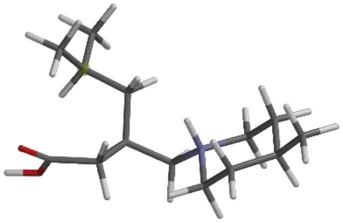

(*S*)-8d

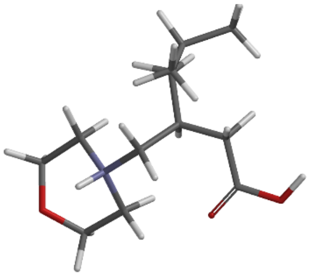

(*R*)-8e

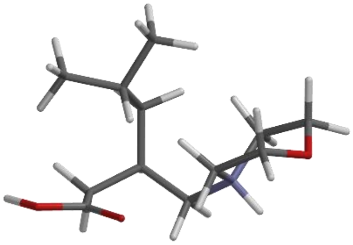

(*S*)-8e

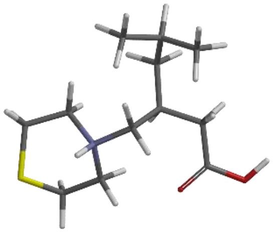

(*R*)-8f

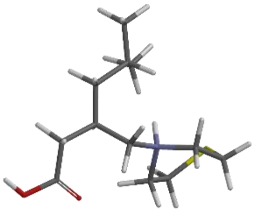

(*S*)-8f

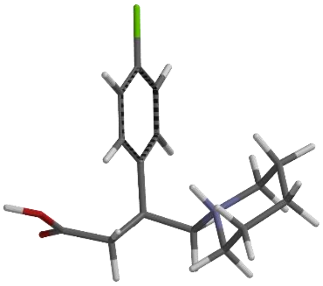

(*R*)-9b

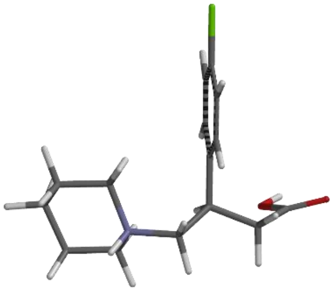

(*S*)-9b

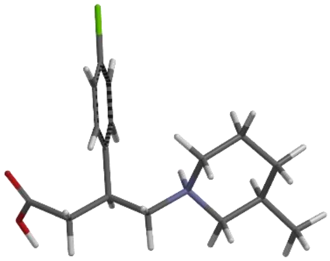

(*R*)-9c

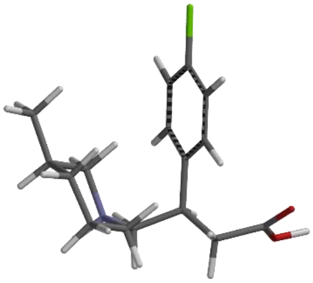

(*S*)-9c

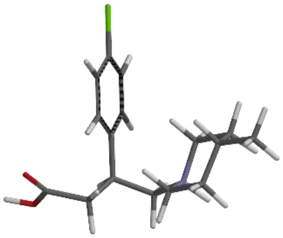

(*R*)-9d

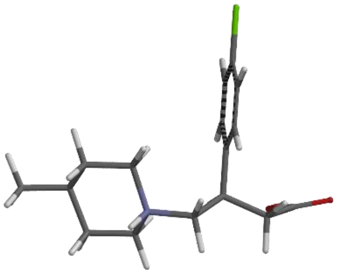

(*S*)-9d

Figure 45S. Optimized structures of all GABA analogues, VPNa and VGB. Continuation

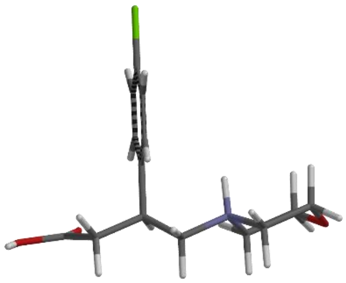

(*R*)-9e

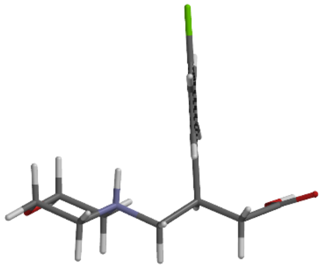

(*S*)-9e

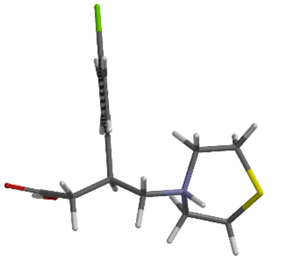

(*R*)-9f

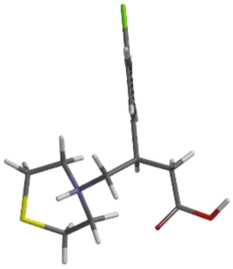

(*S*)-9f

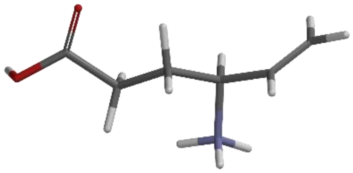

VGB

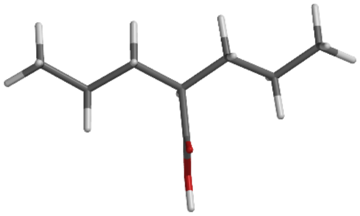

VPNa

Figure 45S. Optimized structures of all GABA analogues, VPNa and VGB. Continuation

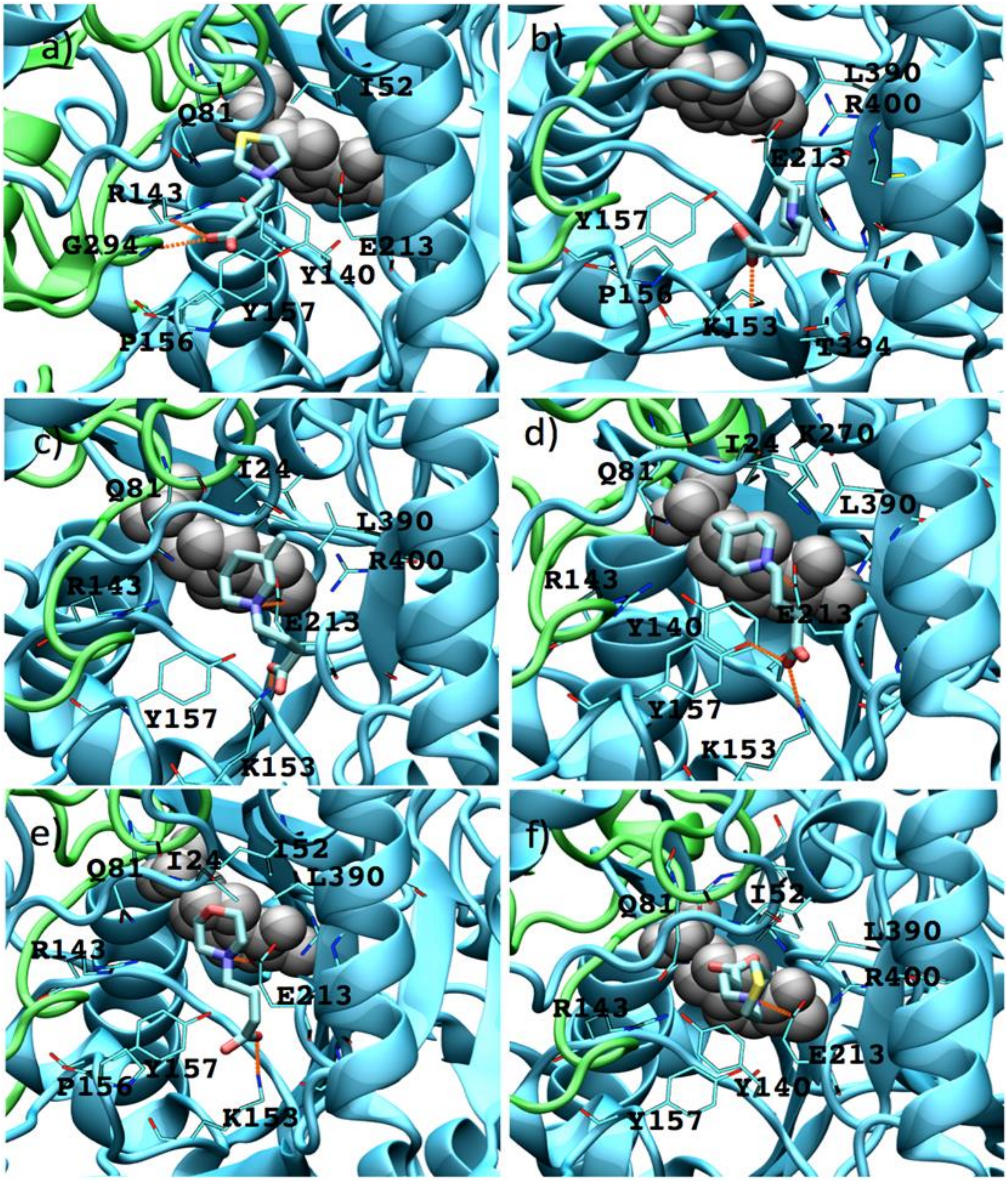

Figure 46S. Interactions between GABA analogues **7** and *Pseudomonas fluorescens* GABA-AT. a) **7a**, b) **7b**, c) **7c**, d) **7d**, e) **7e** and f) **7f**. PLP prosthetic group is showed as Van der Waals spheres and each protein chain is colored in green and cyan. Residues at 4 Å of each analogue are indicated. Hydrogen bonds are shown as orange dashed lines.

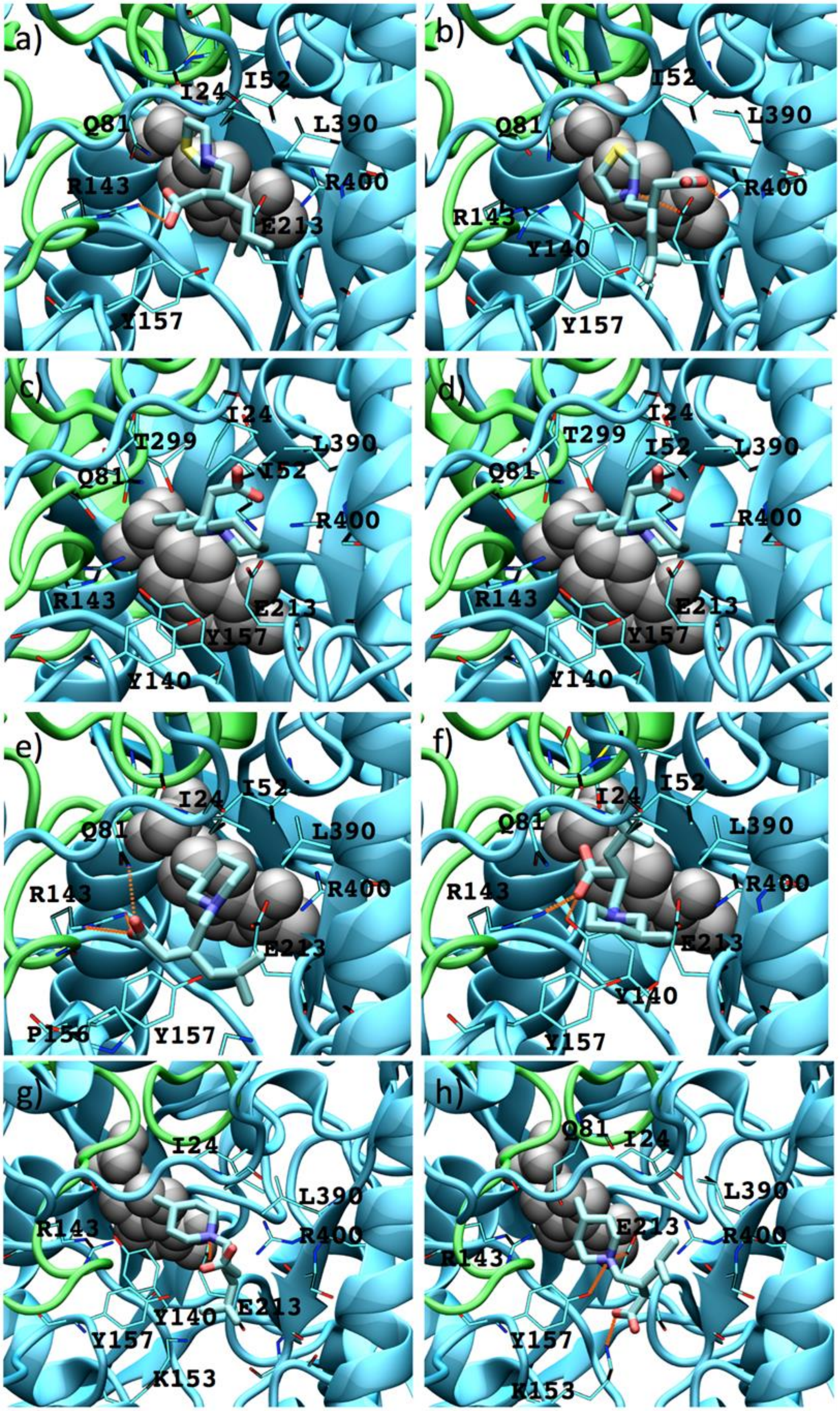
a)
I24
I52
Q81
L390
R143
R400
E213
Y157
b)
I52
Q81
L390
R400
R143
Y140
Y157
c)
I24
T299
L390
Q81
I52
R400
R143
E213
Y157
Y140
d)
I24
T299
I52
L390
Q81
R400
R143
E213
Y157
Y140
e)
I24
I52
Q81
L390
R143
R400
E213
P156
Y157
f)
I24
I52
Q81
L390
R143
R400
E213
Y140
Y157
g)
I24
L390
R143
R400
Y140
E213
Y157
K153
h)
Q81
I24
L390
E213
R400
R143
Y157
K153

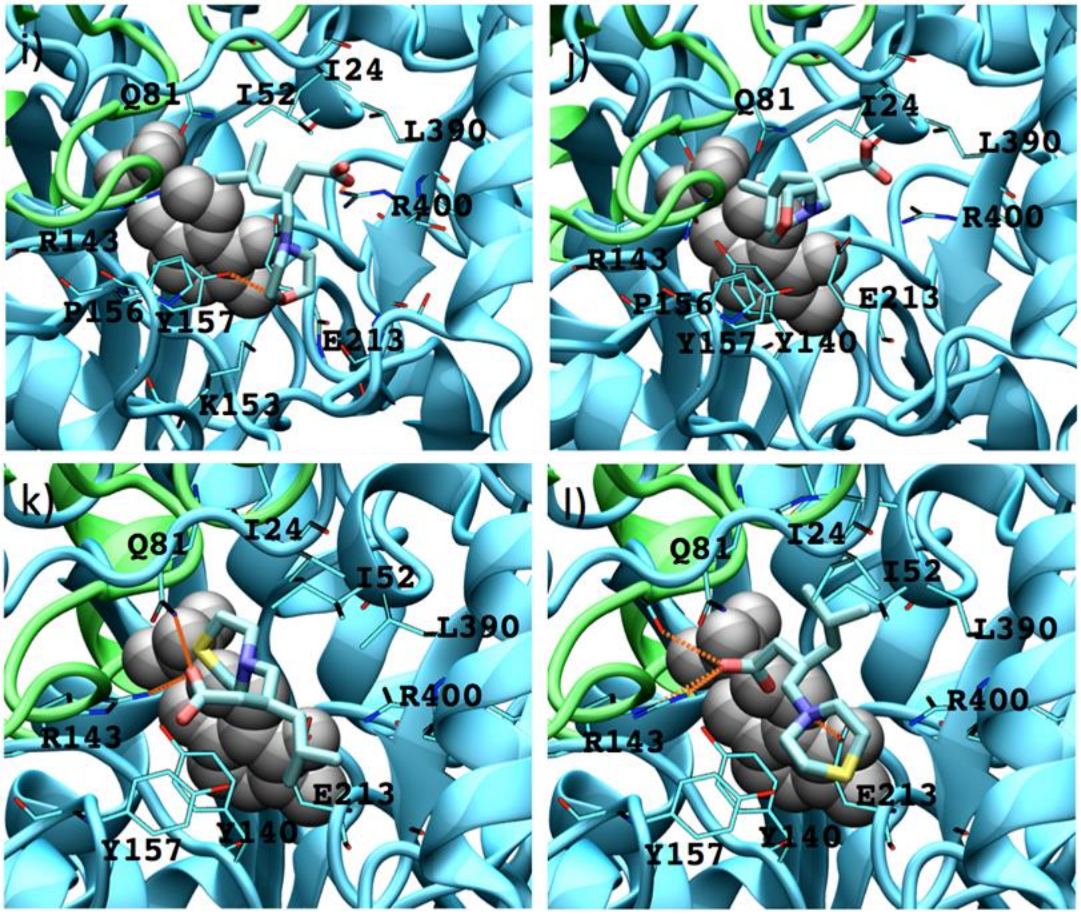

Figure 47S. Interactions between GABA analogues **8** and *Pseudomonas fluorescens* GABA-AT. a) (*S*)-**8a**, b) (*R*)-**8a**, c) (*S*)-**8b**, d) (*R*)-**8b**, e) (*S*)-**8c** and f) (*R*)-**8c**, g) (*S*)-**8d**, h) (*R*)-**8d**, i) (*S*)-**8e**, j) (*R*)-**8e**, k) (*S*)-**8f**, l) (*R*)-**8f**. PLP prosthetic group is showed as Van der Waals spheres and each protein chain is colored in green and cyan. Residues at 4 Å of each analogue are indicated. Hydrogen bonds are shown as orange dashed lines.

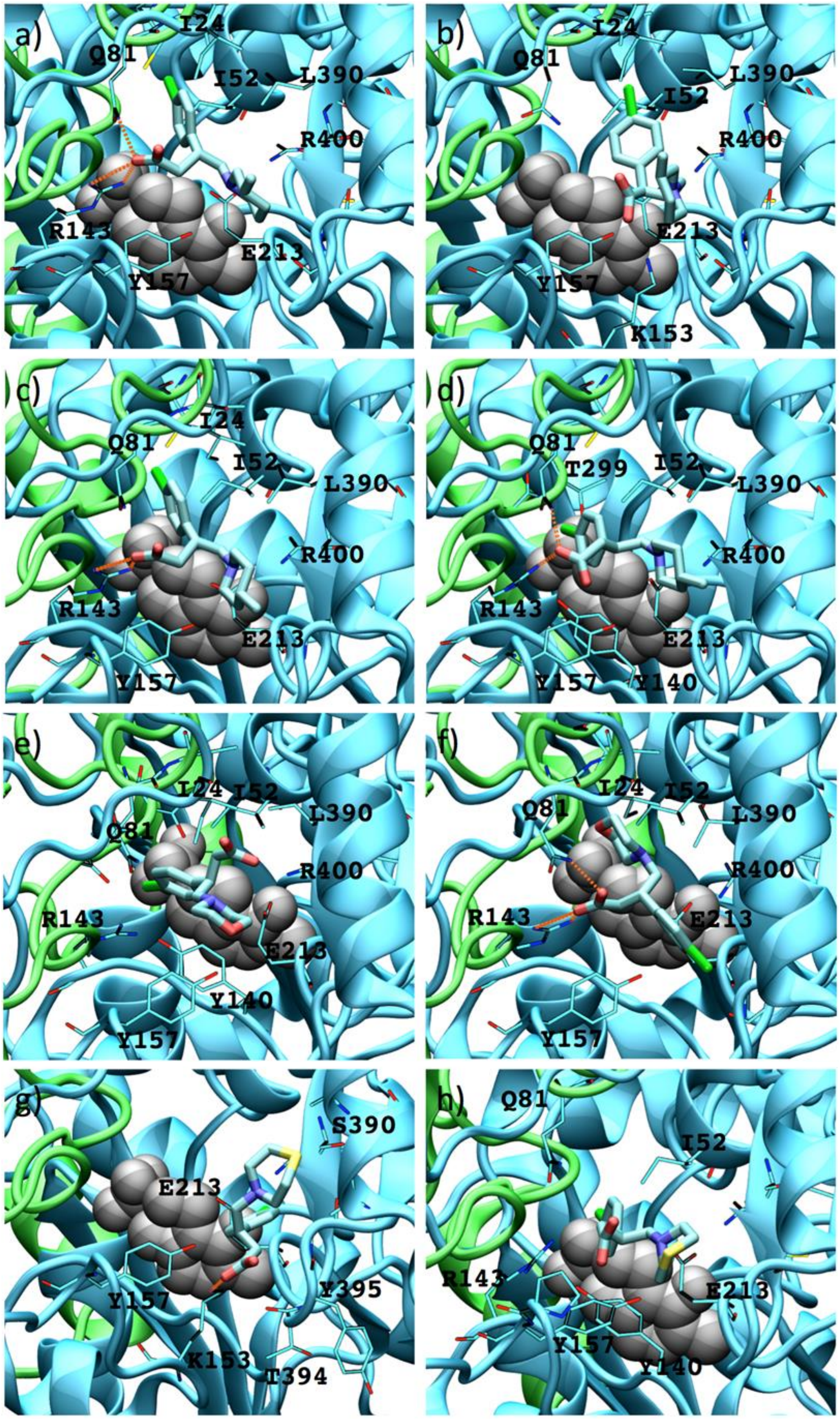
a)
I24
Q81
I52
L390
R400
R143
E213
Y157
b)
I24
Q81
L390
I52
R400
E213
Y157
K153
c)
Q81
I24
I52
L390
R400
R143
E213
Y157
d)
Q81
T299
I52
L390
R400
R143
E213
Y157
Y140
e)
I24
I52
L390
Q81
R400
R143
E213
Y140
Y157
f)
I24
I52
Q81
L390
R400
R143
E213
Y157
g)
S390
E213
Y395
Y157
K153
T394
h)
Q81
I52
R143
E213
Y157
Y140

Figure 48S. Interactions between GABA analogues **9** and *Pseudomonas fluorescens* GABA-AT. a) (*S*)-**9c**, b) (*R*)-**9c**, c) (*S*)-**9d** and d) (*R*)-**9d**, e) (*S*)-**9e**, f) (*R*)-**9e**, g) (*S*)-**9f**, h) (*R*)-**9f**. PLP prosthetic group is showed as Van der Waals spheres and each protein chain is colored in green and cyan. Residues at 4 Å of each analogue are indicated. Hydrogen bonds are shown as orange dashed lines.

Table 1S. Energy interactions values obtained from the docking calculations of all GABA derivatives and *pseudomonas* GABA-AT model. All the values are in kcal/mol.

Ligand	MolDock Score	Electro	HBond
**7a**	-73.1062	-7.11035	-2.65911
**7b**	-69.4043	-3.21553	-7.4961
**7c**	-63.4051	-8.18821	-3.26379
**7d**	-88.9465	-5.28229	-5.17495
**7e**	-81.1331	-10.6664	-5.7402
**7f**	-82.0854	-9.3513	-8.95745
(*S*)-**8a**	-88.4367	-7.59611	-2.37557
(*R*)-**8a**	-80.2747	-4.34932	-1.54938
(*S*)-**8b**	-95.13	-3.82411	-3.42391
(*R*)-**8b**	-82.2497	-1.10123	-9.99797
(*S*)-**8c**	-106.003	-11.4722	-6.60934
(*R*)-**8c**	-84.4955	-2.78647	-5.59387
(*S*)-**8d**	-102.496	-12.1057	-7.491
(*R*)-**8d**	-79.0166	-7.77168	-3.27213
(*S*)-**8e**	-97.1576	-7.49549	-4.62095
(*R*)-**8e**	-85.5462	-5.56573	-1.57816
(*S*)-**8f**	-110.456	-9.48398	-2.62015
(*R*)-**8f**	-80.3773	-1.33976	-5.50014
(*S*)-**9b**	-94.5623	-5.33333	-7.63276
(*R*)-**9b**	-82.3833	-8.45774	-4.99611
(*S*)-**9c**	-105.201	-4.4125	-6.8652
(*R*)-**9c**	-92.4029	0.375594	-4.915
(*S*)-**9d**	-93.087	-3.43971	-3.94599
(*R*)-**9d**	-102.403	-5.70107	-3.30324
(*S*)-**9e**	-97.4871	-4.61057	0
(*R*)-**9e**	-90.7655	-4.02959	-8.75642
(*S*)-**9f**	-92.252	-4.16681	-2.49799
(*R*)-**9f**	-83.726	-0.300184	0
VPNa	-64.703	-5.43962	-6.16675

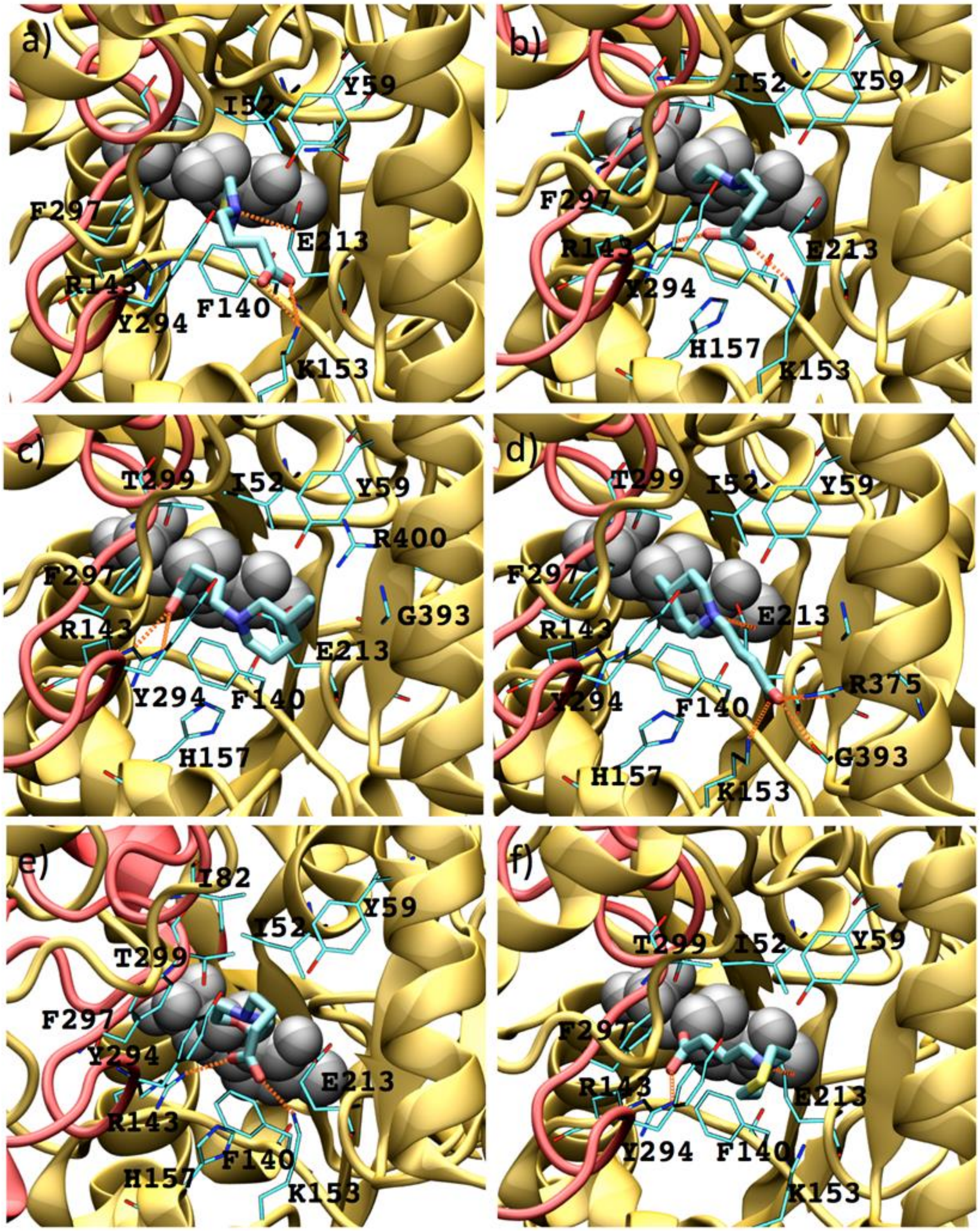

Figure 49S. Interactions between GABA analogues **7** and *Human* GABA-AT. a) **7a**, b) **7b**, c) **7c**, d) **7d**, e) **7e** and f) **7f**. PLP prosthetic group is showed as Van der Waals spheres and each protein chain is colored in yellow and red. Residues at 4 Å of each analogue are indicated. Hydrogen bonds are shown as orange dashed lines.

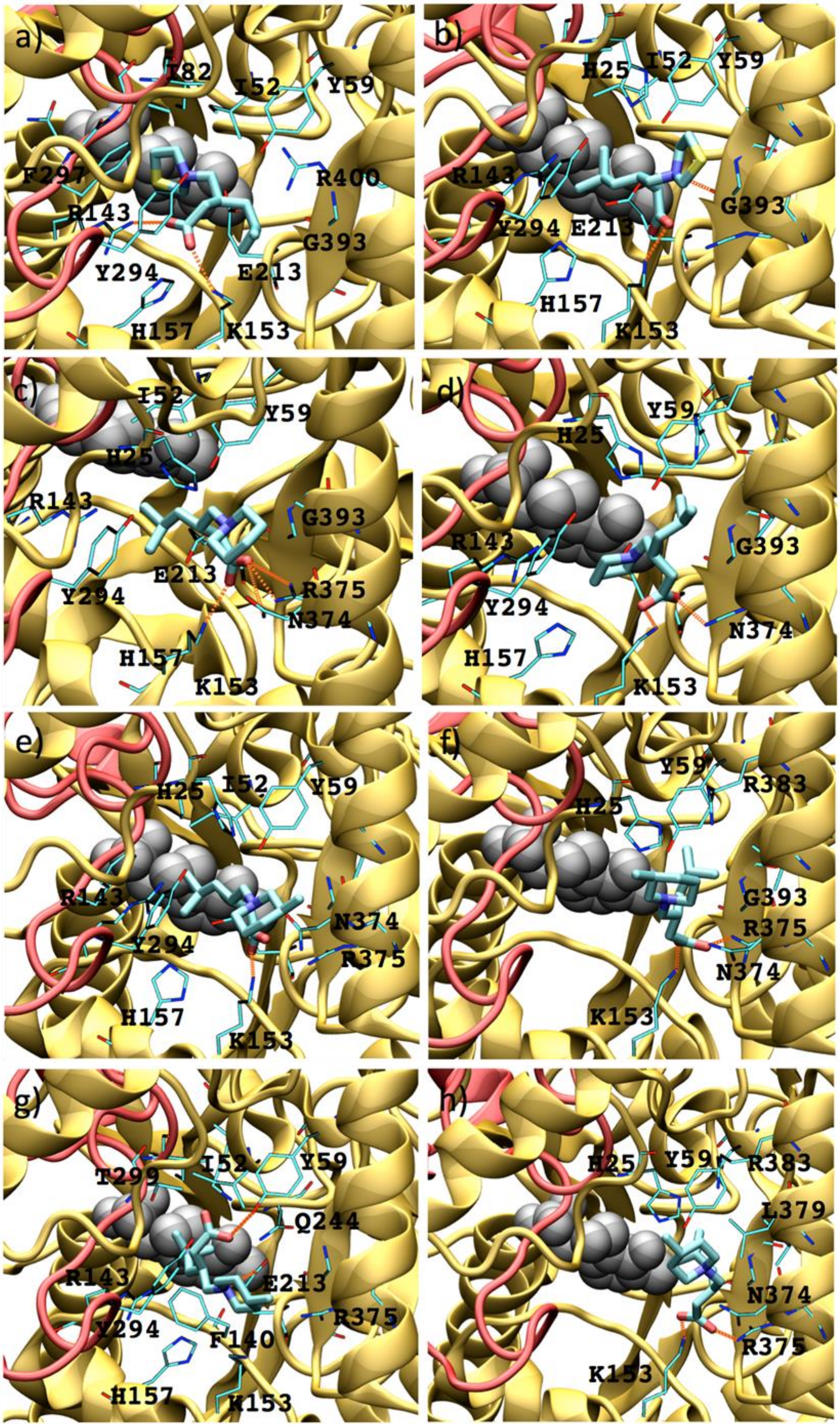
a)
T82
I52
Y59
F297
R400
R143
G393
Y294
E213
H157
K153
b)
H25
I52
Y59
R143
G393
Y294
E213
H157
K153
c)
I52
Y59
H25
R143
G393
E213
R375
Y294
N374
H157
K153
d)
Y59
H25
R143
G393
Y294
N374
H157
K153
e)
H25
I52
Y59
R143
N374
Y294
R375
H157
K153
f)
Y59
R383
H25
G393
R375
N374
K153
g)
T299
I52
Y59
Q244
R143
E213
R375
Y294
F140
H157
K153
h)
H25
Y59
R383
L379
N374
R375
K153

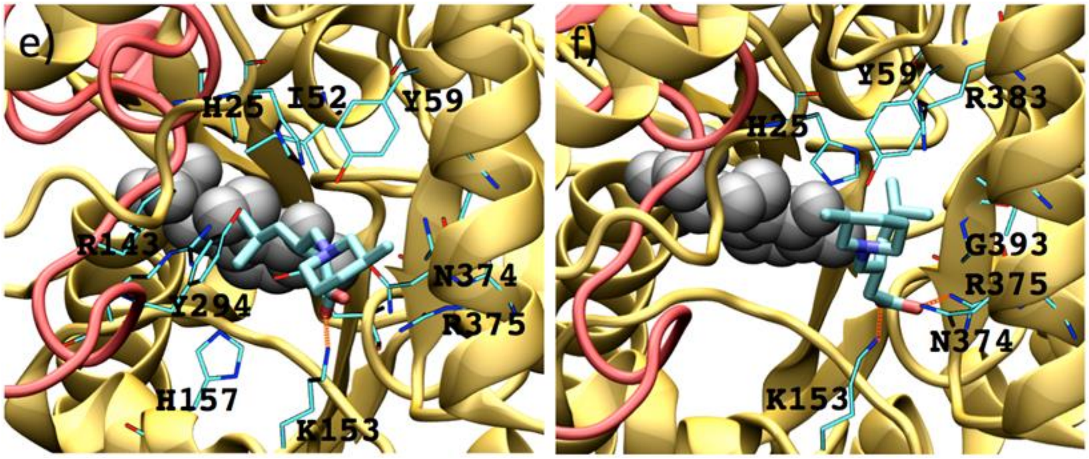

Figure 50S. Interactions between GABA analogues **8** and *Human* GABA-AT. a) (*S*)-**8a**, b) (*R*)-**8a**, c) (*S*)-**8b**, d) (*R*)-**8b**, e) (*S*)-**8c** and f) (*R*)-**8c**, g) (*S*)-**8d**, h) (*R*)-**8d**, i) (*S*)-**8e**, j) (*R*)-**8e**, k) (*S*)-**8f**, l) (*R*)-**8f**. PLP prosthetic group is showed as Van der Waals spheres and each protein chain is colored in yellow and red. Residues at 4 Å of each analogue are indicated. Hydrogen bonds are shown as orange dashed lines.

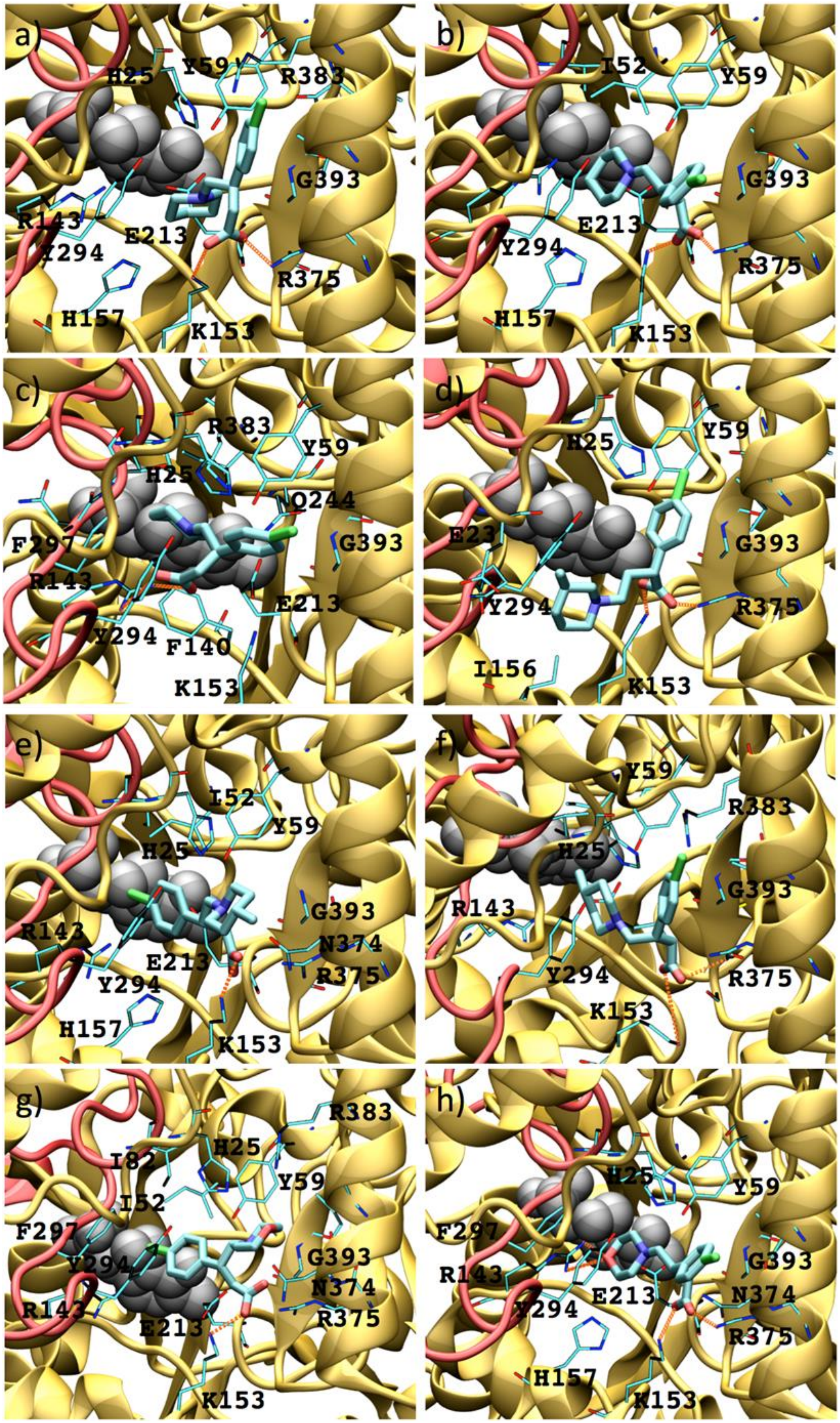
a)
H25
Y59
R383
G393
R143
E213
Y294
R375
H157
K153
b)
I52
Y59
G393
E213
Y294
R375
H157
K153
c)
R383
Y59
H25
Q244
F297
G393
R143
E213
Y294
F140
K153
d)
H25
Y59
E23
G393
Y294
R375
I156
K153
e)
I52
Y59
H25
G393
R143
N374
E213
Y294
R375
H157
K153
f)
Y59
R383
H25
G393
R143
Y294
R375
K153
g)
R383
I82
H25
Y59
I52
F297
G393
Y294
N374
R143
R375
E213
K153
h)
H25
Y59
F297
G393
R143
E213
N374
Y294
R375
H157
K153

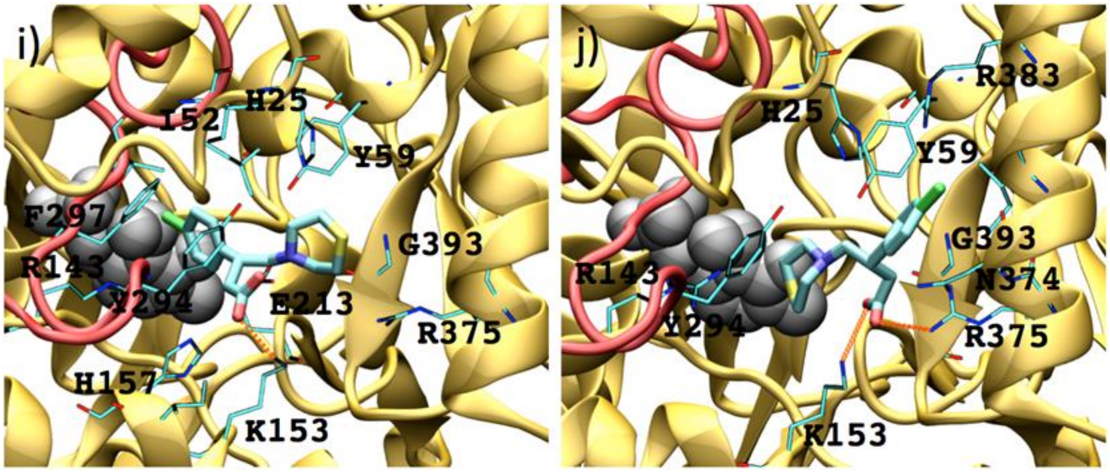

Figure 51S. Interactions between GABA analogues **9** and *Human* GABA-AT. a) (*S*)-**9b**, b) (*R*)-**9b**, c) (*S*)-**9c** and d) (*R*)-**9c**, e) (*S*)-**9d**, f) (*R*)-**9d**, g) (*S*)-**9e**, h) (*R*)-**9e**, i) (*S*)-**9f**, j) (*R*)-**9f**. PLP prosthetic group is showed as Van der Waals spheres and each protein chain is colored in yellow and red. Residues at 4 Å of each analogue are indicated. Hydrogen bonds are shown as orange dashed lines.

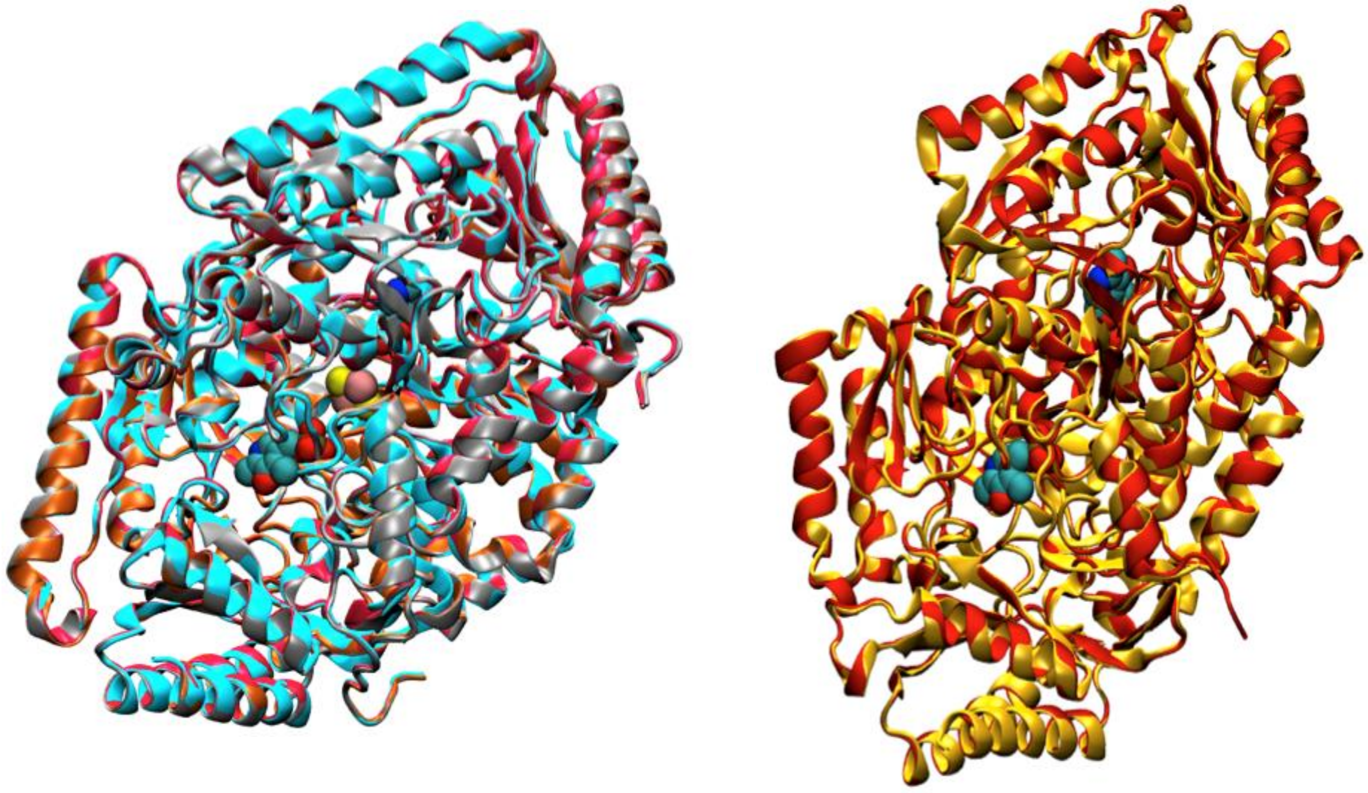

Figure 52S. Backbone structural alignment of GABA-AT structures. a) GABA-AT human model in cyan color. **1ohv**, **1ohw** and **1ohy** Sus scrofa crystal structures in red (RMSD=0.35), gray (RMSD=0.36) and orange (RMSD=0.40) color respectively. b) GABA-AT Pseudomonas fluorescens model in shiny red color, **1sf2** E. coli estructure in shiny yellow color (RMSD= 0.52). Fe_2S_2 (yellow/pink color) and PLP from human model in VDW representation.

Table 2S. Energy interactions values obtained from the docking calculations of all GABA derivatives and *human* GABA-AT model. All the values are in kcal/mol.

Ligand	MolDock Score	Electro	HBond
**7a**	-73.7827	-11.6791	-6.37482
**7b**	-90.0906	-14.3794	-2.70548
**7c**	-87.1437	-4.79249	-4.66131
**7d**	-93.3451	-16.3569	-7.2265
**7e**	-101.729	-10.9936	-4.12353
**7f**	-83.6899	-11.9784	-3.44067
(*S*)-**8a**	-112.119	-7.99459	-3.85277
(*R*)-**8a**	-97.5094	-6.93326	-3.08695
(*S*)-**8b**	-98.5854	-11.0659	-5.84991
(*R*)-**8b**	-93.2925	-12.0228	-1.80199
(*S*)-**8c**	-113.181	-7.06026	-2.5
(*R*)-**8c**	-89.7572	-13.2412	-3.34681
(*S*)-**8d**	-107.919	-2.81265	-4.36016
(*R*)-**8d**	-93.1624	-5.66396	-2.5
(*S*)-**8e**	-111.347	-2.23916	-0.215018
(*R*)-**8e**	-103.812	-14.4581	-3.64372
(*S*)-**8f**	-105.834	-13.386	-2.83697
(*R*)-**8f**	-89.8468	-13.3805	-2.49901
(*S*)-**9b**	-100.144	-8.84278	-3.14615
(*R*)-**9b**	-95.7124	-11.0181	-2.4786
(*S*)-**9c**	-105.791	-11.6063	-2.11356
(*R*)-**9c**	-91.5724	-8.52886	-3.78333
(*S*)-**9d**	-107.209	-10.6489	-2.5
(*R*)-**9d**	-98.4869	-10.3527	-3.17113
(*S*)-**9e**	-109.605	-9.38255	-2.5
(*R*)-**9e**	-112.69	-10.5368	-8.05182
(*S*)-**9f**	-122.362	-10.3354	-2.5
(*R*)-**9f**	-103.599	-9.33766	-2.4934
VPNa	-73.7153	-7.61745	0

Table 3S. Values of the experimental (Y_{Exp}), calculated (Y_{Cal}) and predicted (Y_{Pred}) percent of inhibition of the GABA derivatives. Compounds that were considered form the test validation are marked with a script symbol.

Mol	Y_{Calc1}	Y_{Pred1}	Y_{Calc2}	Y_{Pred2}	Y_{Calc3}	Y_{Pred3}	Y_{Calc4}	Y_{Pred4}	Y_{Calc5}	Y_{Pred5}	Y_{Calc6}	Y_{Pred6}	Y_{Calc7}	Y_{Pred7}	Y_{Calc8}	Y_{Pred8}	Y_{Calc9}	Y_{Pred9}	Y_{Calc10}	Y_{Pred10}	Y_{Exp}
**7a**	25.68	26	-	26.91	25.79	26.19	-	26.18	24.28	23.97	23.71	23.19	25.33	25.52	-	25.84	-	27.67	-	25.35	**24.9**
**7b**	19.65	18.12	20.76	19.59	20.43	19.15	20.19	19.06	19.9	18.33	21.19	20.34	20.47	19.07	20.67	19.4	21.29	19.88	20.11	18.62	**24.9**
**7c**	20.39	20.7	21.49	22.13	-	21.14	20.78	21.14	20.59	21.01	21.92	22.54	21.14	21.73	21.39	22.03	-	22.05	20.8	21.27	**19.4**
**7d**	-	21.45	22.55	22.42	22.17	21.92	21.65	21.28	-	21.59	22.99	23.01	-	22.12	22.42	22.25	23.16	23.29	-	21.8	**22.9**
**7e**	19.65	20.05	20.76	21.45	20.43	21.04	20.19	20.64	19.9	20.41	21.19	21.85	20.47	21.15	-	20.67	21.29	22.46	20.11	20.67	**18.3**
**7f**	28.54	29.91	-	29.8	28.51	29.96	28.67	30.88	26.79	27.01	26.08	25.8	27.87	28.87	28.54	30.86	-	30.67	27.99	29.65	**26.5**
**8a**	0.81	4.52	2.05	5.81	1.59	5.43	-	5.91	-	0.95	-1.26	1.59	2.39	6.86	1.52	5.24	1.82	5.33	1.9	5.54	**-3.5**
**8b**	0.16	-0.12	1.27	1.47	1.46	1.66	4.29	5.94	1.62	2	1.62	1.91	2.49	3.11	1.61	1.85	1.03	1.1	1.73	2.06	**0.84**
**8c**	5.87	5.85	-	6.97	7.01	7.22	8.95	9.64	6.97	7.19	7.35	7.63	7.75	8.17	7.19	7.41	6.96	7.14	-	7.11	**5.96**
**8d**	6.93	7.88	8.03	9.08	8.04	9.03	-	9.81	7.96	9	-	8.41	-	8.73	8.22	9.18	8.06	8.97	8.11	9.2	**2.64**
**8e**	-	0.16	1.27	-0.99	1.46	-0.06	4.29	3.39	1.62	-0.64	1.62	-0.09	2.49	1.08	1.61	0.17	1.03	-0.9	1.73	0.06	**6.2**
**8f**	9.05	5.27	10.31	5.81	9.54	5.85	12.78	9.08	-	8.5	-	6.51	9.89	6.39	9.48	5.49	10.4	5.94	9.61	6.31	**16.8**
**9b***	-	-	-	-	-	-	-	-	-	-	-	-	-	-	-	-	-	-	-	-	**73**
**9c**	-	26.19	27.89	25.5	27.99	12.55	27.69	20.73	28.12	31.25	28.07	52.09	28.17	35.81	-	28.34	27.9	25.78	27.98	27.46	**28**
**9d**	9.21	18.37	8.87	9.17	-	7.64	8.12	7.55	9.23	10.15	-	11.64	9.26	10.4	9.08	16.37	8.96	9.38	8.94	9.34	**8.6**
**9e**	5.14	3.56	5.21	4.03	-	4.45	5.95	5.37	6	5.48	7.43	8.27	6.16	5.54	5.63	4.41	4.99	3.68	5.71	5	**7**
**9f**	15.45	18.65	15.83	17.97	14.21	29.61	16.04	17.9	14.45	14.81	13.7	13.01	-	15.13	15.09	17.44	15.94	18.12	15.17	16.05	**14.2**
**VPNa***	-	-	-	-	-	-	-	-	-	-	-	-	-	-	-	-	-	-	-	-	**40**

* Molecules considered as outliers.
- Compounds considered for the test validation.
